# A Regio- and Stereoselective ω-Transaminase/Monoamine Oxidase Cascade for the Synthesis of Chiral 2,5-Disubstituted Pyrrolidines**

**DOI:** 10.1002/anie.201309208

**Published:** 2014-01-29

**Authors:** Elaine O'Reilly, Cesar Iglesias, Diego Ghislieri, Jennifer Hopwood, James L Galman, Richard C Lloyd, Nicholas J Turner

**Affiliations:** School of Chemistry, University of Manchester Manchester Institute of Biotechnology131 Princess Street, Manchester, M1 7DN (UK); Facultad de Química, Universidad de la República(Uruguay); Chirotech Technology Ltd410 Cambridge Science Park, Milton Road, Cambridge, CB40PE (UK)

**Keywords:** biocatalysis, cascade reactions, monoamine oxidase, pyrrolidines, transaminase

## Abstract

Biocatalytic approaches to the synthesis of optically pure chiral amines, starting from simple achiral building blocks, are highly desirable because such motifs are present in a wide variety of important natural products and pharmaceutical compounds. Herein, a novel one-pot ω-transaminase (TA)/monoamine oxidase (MAO-N) cascade process for the synthesis of chiral 2,5-disubstituted pyrrolidines is reported. The reactions proceeded with excellent enantio- and diastereoselectivity (>94 % *ee*; >98 % *de*) and can be performed on a preparative scale. This methodology exploits the complementary regio- and stereoselectivity displayed by both enzymes, which ensures that the stereogenic center established by the transaminase is not affected by the monoamine oxidase, and highlights the potential of this multienzyme cascade for the efficient synthesis of chiral building blocks.

The exquisite chemo-, regio- and stereoselectivity displayed by enzymes has led to their widespread application as catalysts for stereocontrolled organic synthesis.^1^ These properties, coupled with their ability to catalyze reactions under similar conditions, has enabled the development of elegant multienzyme cascade processes, in which the product formed by the action of the first enzyme becomes the starting material for the subsequent biotransformation.^2^ Such tandem processes alleviate the need for protecting-group manipulations and intermediate purification steps, thus providing cost-effective routes to target molecules.

Among the most synthetically useful biocatalysts for the synthesis of chiral amines are the ω-transaminase (TA) family and variants of monoamine oxidase from *Aspergillus niger* (MAO-N).^3^ TAs are capable of mediating the selective reductive amination of prochiral ketones, thereby providing the corresponding chiral amines.3a–e MAO-N catalyzes the oxygen-dependent conversion of amines into imines and is typically selective for the (*S*)-enantiomer.3f–l Variants of MAO-N have been exploited for the deracemization of primary, secondary, and tertiary amines with diverse structural motifs.3a,f–j The development of several chemoenzymatic routes3c, 4 to industrially important target molecules by employing these two enzyme classes is testament to the advances in protein engineering1a, 5 that have resulted in the development of biocatalysts with the desired substrate scope, selectivity, and stability.

2,5-Disubstituted pyrrolidines are important scaffolds in pharmaceutical compounds^6^ and natural products,^7^ and considerable efforts have been devoted to developing asymmetric routes to both *cis*- and *trans-*disubstituted derivatives that show moderate to good stereoselectivity.^8^ The lack of stereofacial bias induced by the preexisiting C2-stereocenter means that obtaining the *trans*-diastereomers through reduction of the corresponding imine in high *de* is not straightforward. Our approach (Scheme 1) features a highly selective TA-mediated reductive amination of an achiral 1,4-diketone to generate an optically active pyrroline followed by diastereoselective chemoenzymatic conversion into the corresponding pyrrolidine by MAO-N/NH_3_⋅BH_3_.

**Scheme 1:**
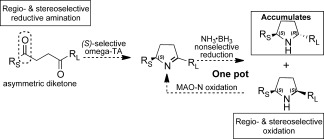
A chemoenzymatic approach for the synthesis of 2,5-disubstituted pyrrolidines by employing an ω-transaminase (TA) and a monoamine oxidase (MAO-N).

Initially, we examined the ω-TA-mediated selective monoamination of commercially available 1,4-diketone **1 a**, which bears a small methyl substituent and a large phenyl substituent (Scheme 2). The first example of the asymmetric bioamination of 1,5-diketones was recently reported with excellent regio- and stereoselectivity achieved.^9^ We found the commercially available (*S*)-selective transaminase ATA113 to be highly regioselective in mediating the reductive amination of **1 a** exclusively on the methyl ketone at a substrate concentration of 25 mm with l-alanine as the amine donor. The resulting 1,4-amino ketone (*S*)-**2 a** subsequently underwent spontaneous cyclization to provide pyrroline (*S*)-**3 a** in high yield (91 %) and excellent *ee* (>99 %). The lactate dehydrogenase (LDH)/glucose dehydrogenase (GDH) system was used to drive the equilibrium towards the product and recycle the NAD^+^ cofactor (see the Supporting Information). The (*R*)-selective transaminase ATA117 also catalyzed the regio- and stereoselective monoamination of **1 a** to afford pyrroline (*R*)-**3 a** in 65 % yield and >99 % *ee*.

**Scheme 2:**
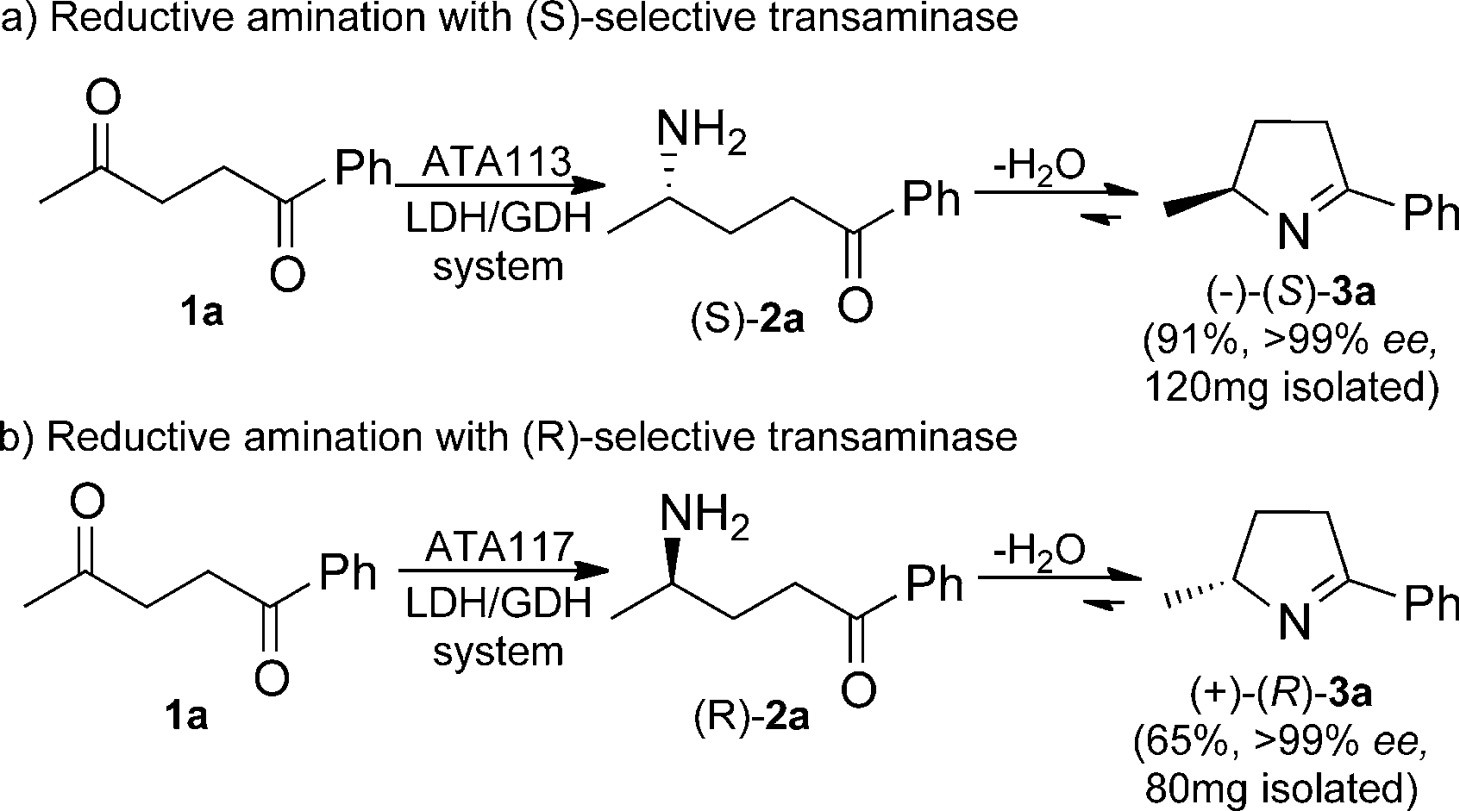
Preparative-scale (25 mm **1 a**) reductive amination of diketone **1 a** mediated by (*S*)-selective ATA113 or (*R*)-selective ATA117, followed by spontaneous cyclization.

Having established an effective means of accessing optically pure pyrrolines on a preparative scale, we subsequently explored a route for the diastereoselective synthesis of 2,5-disubstituted pyrrolidine **4 a** starting from **3 a** (Scheme 3). 2,6-Disubstituted piperidines have been prepared through a chemoenzymatic route employing an ω-transaminase followed by diastereoselective hydrogenation using Pd/C.^9^ However, the same strategy is not applicable to the diastereoselective synthesis of 2,5-disubstituted pyrrolidines owing to poor diastereoselectivity in the reduction step. We envisaged using MAO-N variants in combination with NH_3_⋅BH_3_ for the asymmetric synthesis of **4 a**. Two MAO-N variants (D5 and D9) were selected based on their known activity and excellent selectivity towards structurally related amine frameworks, including pyrrolidines and piperidines.3h,k Our strategy relies upon MAO-N variants displaying complete regio- and stereoselectivity to avoid stereorandomization of the C2-stereocenter generated by the (*S*)-selective ω-TA. Imine **3 a** is in equilibrium with the open-chain amino ketone (*S*)-**2 a** and hence optimization of the MAO-N/NH_3_⋅BH_3_ oxidation/reduction cycle was necessary in order to prevent the formation of undesired amino alcohol as a side product. Ketone reduction was minimized by lowering the concentration of the MAO-N biocatalyst during the reaction while maintaining a high concentration of NH_3_⋅BH_3_. Under these conditions, rapid reduction of pyrroline **3 a** occurred, thus ensuring that a minimal concentration of the amino ketone was present during the biotransformation.

**Scheme 3:**
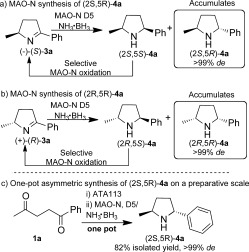
a, b) Analytical-scale synthesis of (2*S*,5*R*)- and (2*R*,5*R*)-**4 a** mediated by MAO-N D5. c) One-pot TA/MAO-N cascade for the preparative-scale asymmetric synthesis of (2*S*,5*R*)-**4 a**. Reduction of the starting diketone by NH_3_⋅BH_3_ prevented the addition of all of the reagents concurrently.

Treatment of (*S*)-**3 a** with NH_3_⋅BH_3_ afforded **4 a** initially as a mixture of diastereoisomers with a slight excess of the (2*S*,5*S*)-isomer (*de*≈10 %; Scheme 3 a). Both MAO-N variants mediated the oxidation of the (2*S*,5*S*)-**4 a** diastereoisomer exclusively and displayed complete regioselectivity for the more bulky phenyl side of the pyrrolidine. Following successive rounds of selective oxidation with the MAO-N D5 variant and nonselective reduction with NH_3_⋅BH_3_, (2*S*,5*R*)-**4 a** was isolated in greater than 99 % *de*. Despite a bias towards the formation of the *cis* diastereoisomer upon reduction with NH_3_⋅BH_3_, the combination with MAO-N yielded solely the *trans* reduction product (2*S*,5*R*)-**4 a**. The complementary regioselectivity displayed by the ω-TA and MAO-N variants circumvents epimerization of the (*S*)-C2-center and provides a method for accessing optically pure 2,5-pyrrolidines.

Having developed efficient individual biocatalytic routes for the synthesis of optically pure pyrroline **3 a** and the target chiral 2,5-pyrrolidine **4 a**, we next sought to combine the ω-TA and MAO-N biocatalysts in a one-pot cascade (Scheme >3 c). Diketone **1 a** was exposed to ATA 113 followed by MAO-N and NH_3_⋅BH_3_, and the target (2*S*,5*R*)-**4 a** was obtained in 82 % yield and >99 % *de*.

To allow access to the (2*R*,5*R*)-**4 a** diastereoisomer, the (*R*)-**3 a** enantiomer, derived from the use of ATA117, was treated with the NH_3_⋅BH_3_/MAO-N combination (Scheme 3b). Following nonselective reduction to give a mixture of (2*R*,5*S*)-**4 a** and (2*R*,5*R*)-**4 a**, both MAO-N variants mediated the selective oxidation of the (2*R*,5*S*)-isomer to provide (2*R*,5*R*)-**4 a** exclusively after successive rounds of oxidation/reduction. The stereochemistry at C2 has a minimal effect on the activity of the MAO-N enzyme and no effect on the stereoselectivity; the target (2*R*,5*R*)-**3 a** was isolated in >99 % *de*.

The generality of the TA/MAO-N cascade process was investigated by examining a series of diketones (**1 b**–**g**) using ATA113, as well as a novel transaminase (*pf*-ATA) from *Pseudogulbenkiania ferrooxidans*^10^ (Table 1). *Pf*-ATA shares 95 % sequence identity with the transaminase from *Chromobacterium violaceum* (*cv*-ATA; ATCC 12472).^11^ ATA113 mediated the reductive amination of diketones **1 b**–**g**, the products of which spontaneously cyclized to yield pyrrolines **3 b**–**g** as the sole regioisomers with excellent conversion and high *ee* values. Unsurprisingly, replacement of the small methyl group by a larger ethyl substituent resulted in a slightly reduced *ee* (entries 11 and 13). The biotransformations performed with *Pf*-ATA proceeded with reduced selectivity, with *ee* values lower than those achieved with ATA113. Interestingly, replacing the methyl substituent by an ethyl group resulted in a switch in stereoselectivity to give (*R*)-**3 f**, **g** as the predominant enantiomers (entries 12 and 14, see the Supporting Information for absolute configuration and *ee* determination). We also compared the selectivity observed with *pf*-ATA to that of the related *cv*-ATA against diketones **1 a** and **1 d**, **e** and noted comparable conversion and selectivity (see the Supporting Information). The (*R*)-selective ATA117 also mediated the reductive amination of diketones **1 a** and **1 d**, **e** in >99 % conversion and *ee* (see the Supporting Information).

**Table 1:** TA-mediated reductive amination of 1 a–g. 
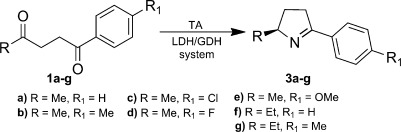

Entry	Substrate	ω-TA	Conv. [%]	*ee* [%]
1	**1 a**	ATA113	>99	>99 (*S*)
2		*P. ferrooxidans*	>99	75 (*S*)
3	**1 b**	ATA113	>99	>99 (*S*)
4		*P. ferrooxidans*	>99	>78 (*S*)
5	**1 c**	ATA113	>99	>99 (*S*)
6		*P. ferrooxidans*	>99	68 (*S*)
7	**1 d**	ATA113	>99	>99 (*S*)
8		*P. ferrooxidans*	>99	76 (*S*)
9	**1 e**	ATA113	>99	>99 (*S*)
10		*P. ferrooxidans*	>99	78 (*S*)
11	**1 f**	ATA113	60	96 (*S*)
12		*P. ferrooxidans*	>99	76 (*R*)
13	**1 g**	ATA113	>99	94 (*S*)
14		*P. ferrooxidans*	75	46 (*R*)

The efficiency of the MAO-N/NH_3_⋅BH_3_ step with the isolated pyrrolines **3 b**–**g** was next examined (Table 2). In general, the D9 variant showed higher selectivity and employing either the D5 or D9 MAO-N variants allowed access to all of the Me/Ar and Et/Ar substituted pyrrolidines in excellent *de*. We have also extended the one-pot TA/MAO-N cascade for the synthesis of (2*S*,5*R*)-**4 b**, (2*S*,5*R*)-**4 d,** and (2*S*,5*R*)-**4 e** in >99 % conversion and >99 % *de* when starting from the corresponding diketones (Table 3), thus demonstrating the generality of this one-pot approach.

**Table 2:** MAO/NH_3_⋅BH_3_-mediated asymmetric synthesis of (2*S*,5*R*)- 4 a–g 
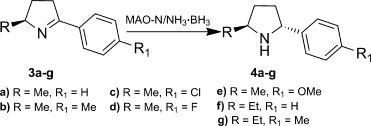

Entry	Substrate	MAO-N variant	*de* [%] (2*S*,5*R*)
1	**3 a**	D5	>99
2		D9	96
3	**3 b**	D5	88
4		D9	98
5	**3 c**	D5	>99
6		D9	>99
7	**3 d**	D5	>99
8		D9	>99
9	**3 e**	D5	68
10		D9	>99
11	**3 f**	D5	64
12		D9	>99
13	**3 g**	D5	56
14		D9	96

**Table 3:** ATA113/MAO-N one-pot cascade for the synthesis of (2*S*,5*R*)-4 a, (2*S*,5*R*)-4 b, (2*S*,5*R*)-4 d, and (2*S*,5*R*)-4 e

Ketone	ω-TA	MAO-N	Conv. [%]	*de* [%]
**1 a**^[a]^	ATA113	D5	>99	>99 (2*S*,5*R*)-**4 a**
**1 b**^[b]^	ATA113	D9	>99	>99 (2*S*,5*R*)-**4 b**
**1 d**^[b]^	ATA113	D9	>99	>99 (2*S*,5*R*)-**4 d**
**1 e**^[b]^	ATA113	D9	>99	>99 (2*S*,5*R*)-**4 e**

[a] 25 mm substrate; [b] 5 mm substrate.

In summary, the combination of two complementary biocatalysts has been demonstrated in a novel one-pot chemoenzymatic cascade for the regio- and stereoselective synthesis of a panel of 2,5-disubstituted pyrrolidines from the corresponding 1,4-diketones. The transaminase ω-TA is highly selective for the sterically less demanding methyl ketone while the monoamine oxidase MAO-N shows an overwhelming preference for the more bulky portion of the corresponding pyrrolidine. The compatibility of the two biocatalysts means that the reaction can be performed in one pot without the need for costly intermediate purification steps. The chemoenzymatic approach exploits four distinct biocatalytic operations and takes advantage of the complementary regioselectivity displayed by the ω-TA and MAO variants to establish two stereogenic centers. All of the biocatalysts described herein are commercially available^12^ and hence readily accessible for practical application.

## Supporting Information

As a service to our authors and readers, this journal provides supporting information supplied by the authors. Such materials are peer reviewed and may be re-organized for online delivery, but are not copy-edited or typeset. Technical support issues arising from supporting information (other than missing files) should be addressed to the authors.

miscellaneous_information
