# Supplementary material for: A Regio- and Stereoselective ω-Transaminase/Monoamine Oxidase Cascade for the Synthesis of Chiral 2,5-Disubstituted Pyrrolidines
Source: Angew Chem Int Ed Engl. 2014 Jan 29;53(9):2447–50. doi: 10.1002/anie.201309208 (PMC4227563; doi:10.1002/anie.201309208)
Supplement: Supplementary file 1 — miscellaneous_information [file anie0053-2447-SD1.pdf]

Angewandte Chemie
Eine Zeitschrift der Gesellschaft Deutscher Chemiker

Supporting Information
© Wiley-VCH 2014
69451 Weinheim, Germany

A Regio- and Stereoselective ω-Transaminase/Monoamine Oxidase Cascade for the Synthesis of Chiral 2,5-Disubstituted Pyrrolidines**

*Elaine O'Reilly, Cesar Iglesias, Diego Ghislieri, Jennifer Hopwood, James L. Galman, Richard C. Lloyd, and Nicholas J. Turner**

anie_201309208_sm_miscellaneous_information.pdf

Supplementary Information

Table of Contents:

General methods and materials **S2**
Identification of the aminotransferase gene and construction of the expression vector pET16b-pf-ATA **S2**
DNA sequences and translated amino acid sequences of *pf*-ATA **S2**
Expression of ω-transaminases in *E. coli* BL21(DE3) **S3**
Synthesis of 1,4-diketones **1b-g** **S4**
^{1}H-NMR data for **1b-g** **S4**
ω-TA-mediated reductive amination of 1,4-diketones **1a-g** **S5**
NMR data for **3a-g** **S6**
Preparation of (2*S*,5*S*)- and (2*S*,5*R*)-2,5-pyrrolidine standards **4a-g** **S7**
NMR data for (2*S*,5*S*)- and (2*S*,5*R*)-2,5-pyrrolidine standards **4a-g** **S8**
Analytical scale deracemisations with MAO-N variants **S9**
One-pot transaminase/MAO-N cascade for the preparative synthesis of (2*S*,5*R*)-**4a** and for the analytical scale synthesis of (2*S*,5*R*)-**4b**, (2*S*,5*R*)-**4d** and (2*S*,5*R*)-**4e** **S10**
NMR Spectra of ATA113 reductive amination products (*S*)-**3a-g** **S11**
NMR spectra of **4a-g** standards following treatment with sodium borohydride **S18**
NMR spectra of ATA113/MAO-N D9 one-pot cascade product (2*S*,5*R*)-**4a** **S25**
GC/FID Chromatograms of (*S*)-**3a-g** following ATA113 preparative-scale reactions with diketones **1a-g** **S26**
Determination of *enantiomeric excess* and assignment of absolute configuration **S28**
Determination of *diastereomeric excess* of 2,5-disubstituted pyrrolidines **4a-g** after MAO-N mediated reaction **S36**
References **S39**

General Methods and Materials

General: All biotransformations were carried out in HEPES buffer (100mM, pH 7.5) at 30 ºC. ^{1}H and ^{13}C NMR spectra were recorded on a Bruker Avance 400 spectrometer (400.1 MHz for ^{1}H and and 100.6 MHz for ^{13}C) in $CDCl_3$. The chemical shifts were recorded in ppm with the residual $CHCl_3$ signal referenced to 7.26 ppm and 77.23 ppm for ^{1}H and ^{13}C respectively. Coupling constants (*J*) are reported in Hz and refer to the apparent peak multiplicities. TLC analysis was performed using aluminium foil-backed TLC plates with 0.2 mm silica. Flash column chromatography was performed on silica gel (200-300 mesh). GC-MS spectra were recorded on a Hewlett Packard HP 6890 equipped with a HP-1MS column, a HP 5973 Mass Selective Detector and an ATLAS GL FOCUS sampling robot. GC-FID analysis was performed on Agilent 6850 equipped with a Gerstel Multipurposesampler MPS2L and a Varian CP CHIRASIL-DEX CB 25 m x 0.25 mm DF=0.25 column.

Materials: Commercially available reagents were used throughout without further purification. 1-Phenyl-1,4-pentanedione and all other reagents were purchased from Sigma Aldrich or Acros including anhydrous solvents. *Escherichia coli* BL21(DE3) were purchased from Invitrogen (Carlsbad, CA).
Pfu polymerase was purchased from Stratagene (La Jolla, CA) and dNTPs was purchased from Fermentas (Waltham, MA). Expression vector pET-16b (69662-3) was purchased from Novagen (Darmstadt, Germany). Commercially available transaminases, ATA113 and ATA117 were purchased from Codexis in the form of lyophilised cell extract. *Chromobacterium violaceum* ATA[1] (cv-ATA, ATCC 12472, NCIMB 9178) was a kind gift from Prof. Wolfgang Kroutil. *Pseudogulbenkiania ferrooxidans* (pf-ATA) was isolated from Chirotech Microbacterial culture collection (CMCC, 104818).

Identification of the aminotransferase gene and construction of the expression vector pET16b-*pf*-ATA

The coding region of the ATA gene was amplified by PCR from CMCC using forward (5'CAT-ATGCAGAAGCAACGTACGACCAGCC-3') and reverse (5'-CTCGAGCTAAGCCAGCCCGCGCGCCTT-CAG -3') primers with *NdeI* and *XhoI* restriction sites respectively. The following PCR protocol was used: 2 min denaturation at 95 °C and then 30 cycles of 15 s denaturation at 94 °C, 30 s annealing at 62 °C and 90 s elongation at 68 °C. The PCR product was cloned into ZERO Blunt TOPO PCR Cloning vector (K2830-20) following the manufacturers protocol. The DNA construct was sequenced and translated to the amino acid sequence. A blast search was performed using the NCBI database http://blast.ncbi.nlm.nih.gov/Blast.cgi confirming that the gene had a 99% sequence identity to the *P. ferrooxidans* aminotransaminase (Genbank accession no. WP_021478068). The cloning vector was then digested with *NdeI/XhoI* with the resulting 1.3 kb fragment subcloned into pET16b expression vector.

DNA sequences and translated amino acid sequences of *pf*-ATA

The aminotransferase gene from *Pseudogulbenkiania ferrooxidans* is in bold with the 10x His-tag underlined.

ATGGGC<u>CATCATCATCATCATCATCATCATCATCAC</u>AGCAGCGGCCATATCGAAGGTCGT
CAT**ATGCAGAAGCAACGTACGACCAGCCAATGGCGCGAACTGGATGCCGCCCATCA**
CCTGCATCCGTTTACCGATACCGCATCGCTGAACCAGGCGGGCGCGCGCGTGATGA
CGCGCGGAGAAGGCATCTATCTGTGGGATTCGGACGGCAACAAGATCATCGACGGC

**ATGGCCGGCCTATGGTGTGTGAACGTCGGCTACGGCCGCAAGGATTTCGCCGAGGT
GGCGCGCCGCCAGATGGAAGAGCTGCCGTTCTACAACACCTTCTTCAAGACCACGC
ATCCGGCGGTGGTCGAACTGTCCCGCCTGTTGGCCGAAGTGACGCCAGCCGGCTTC
GACCACGTGTTTTACACCAACTCAGGCTCGGAGTCGGTGGACACCATGATCCGCAT
GGTGCGCCGCTACTGGGACGTGCAGGGCAAGCCGGAGAAGAAGACGCTGATCGGC
CGTTGGAACGGCTATCACGGCTCCACCATCGGCGGCGCCAGTCTGGGCGGCATGAA
GTACATGCATGAGCAGGGCGATCTGCCGATTCCGGGCATGGCCCATATCGAGCAGC
CGTGGTGGTACAAGCACGGCAAGGACATGACGCCGGACGAATTCGGCGTGGTGGC
CGCGCGCTGGCTGGACGAGAAGATCCAGGAGATCGGCGCCGACAAGGTGGCGGCT
TTTGTCGCCGAACCCATCCAGGGCGCTGGCGGGGTGATCATACCGCCAGCCACCTA
CTGGCCGGAAATCGAGCGCATCTGCCGCAAGCACGATGTGTTGATTGTGGCGGACG
AAGTGATCTGCGGTTTCGGCCGCACCGGCGAATGGTTCGGCCATCAGCATTTCGGC
TTCCAGCCTGACCTGTTCACCGCGGCTAAGGGCTTGTCGTCGGGCTACTTGCCGAT
CGGCGCGGTGTTCGTCGGCAAGCGCGTGGCGGAAGGCCTGATCGCCGGCGGCGAC
TTCAACCACGGCTTCACCTATTCCGGCCATCCGGTCTGCGCCGCGGTGGCGCACGC
CAATGTGGTGGCGCTGCGAGACGAGGGCATCGTCCAGCGCGTCAAGGACGACATC
GGTCCTTACATGCAGAAGCGTTGGCGCGAGACCTTCAGTCAGTTCGAGCACGTGGA
CGACGTGCGCGGCGTCGGCCTGATCCAAGCCTTCACGCTGGTGAAGAACAAGGCG
AAGCGCGAGCTGTTCCCCGATTTCGGCGAGGTCGGCACGCTATGCCGCGACATCTT
CTTCCGCAACAACCTGATCATGCGCGCTTGCGGCGACCATATCGTATGTTCGCCGC
CGCTGGTGATGACGCGAGCCGAAGTGGATGAAATGCTGGGGGTGGCGGCGCGTTG
CCTAGCCGAGTTCGAGCAGGCGCTGAAGGCGCGCGGGCTGGCTTAA**

MG<u>HHHHHHHHHH</u>SSGHIEGRH**MQKQRTTSQWRELDAAHHLHPFTDTASLNQAGARVM
TRGEGIYLWDSDGNKIIDGMAGLWCVNVGYGRKDFAEVARRQMEELPFYNTFFKTTH
PAVVELSRLLAEVTPAGFDHVFYTNSGSESVDTMIRMVRRYWDVQGKPEKKTLIGRWN
GYHGSTIGGASLGGMKYMHEQGDLPIPGMAHIEQPWWYKHGKDMTPDEFGVVAARW
LDEKIQEIGADKVAAFVAEPIQGAGGVIIPPATYWPEIERICRKHDVLIVADEVICGFGRT
GEWFGHQHFGFQPDLFTAAKGLSSGYLPIGAVFVGKRVAEGLIAGGDFNHGFTYSGHP
VCAAVAHANVVALRDEGIVQRVKDDIGPYMQKRWRETFSQFEHVDDVRGVGLIQAFTL
VKNKAKRELFPDFGEVGTLCRDIFFRNNLIMRACGDHIVCSPPLVMTRAEVDEMLGVA
ARCLAEFEQALKARGLA.**

Expression of ω-transaminases in *E. coli* BL21(DE3)

E. coli BL21 (DE3) cells were transformed with pET16b-*pf*-ATA or pET21a-*cv*-ATA. An overnight cell culture was used to inoculate a 500 mL LB medium supplemented with 100 µg/mL Ampicillin and incubated at 37 ºC at 250 rpm until an OD_{600} of 0.5-0.6 was reached. The recombinant protein expression was induced by adding IPTG (2 mM) and incubated at 18°C for a further 16h. The cells were harvested by centrifugation at 4 °C, and resuspended in 20 mL lysis buffer containing 100mM potassium phosphate buffer pH 7.0, with 0.1 mM PLP. The cell pellets were lysed in an iced bath by ultra-sonication by Soniprep 150 (10 cycles of 20 s on/20 s off.). The sonicated suspension was centrifuged at 20,000 rpm for 20 min at 4 °C. The supernatant (soluble protein) was collected and used as 'clarified cell extract'.

Synthesis of 1,4-diketones 1b-g

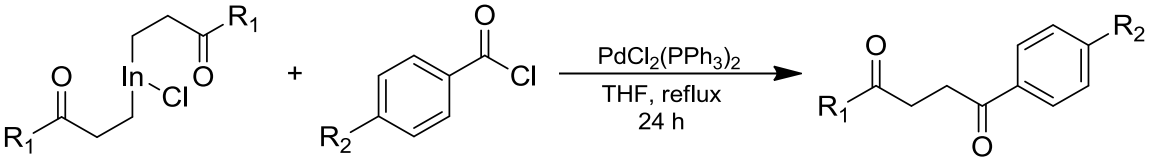

1b-g

1,4-diketones **1b-g** were synthesised following a procedure reported by Shen *et. al.*[2]

1-(p-tolyl)pentane-1,4-dione (1b)

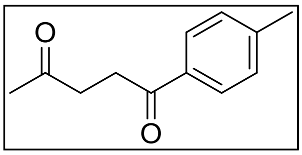

^{1}H NMR (400 MHz, $CDCl_3$) δ 7.87 – 7.83 (m, 2H), 7.25 – 7.20 (m, 2H), 3.25-3.21 (m, 2H), 2.85 (t, *J* = 6.3 Hz, 2H), 2.38 (s, 3H), 2.23 (s, 3H); ^{13}C NMR (101 MHz, $CDCl_3$) δ 207.7, 198.4, 144.2, 134.4, 129.5, 128.4, 37.3, 32.5, 30.4, 21.9.

1-(4-chlorophenyl)pentane-1,4-dione (1c)

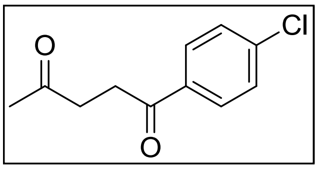

^{1}H NMR (400 MHz, $CDCl_3$) δ 7.94 – 7.90 (m, 2H), 7.46 – 7.41 (m, 2H), 3.26-3.21 (m, 2H), 2.91-2.87 (m, 2H), 2.26 (s, 3H); ^{13}C NMR (101 MHz, $CDCl_3$) δ 207.4, 197.5, 139.8, 135.2, 129.7, 129.1, 37.2, 32.6, 30.3.

1-(4-fluorophenyl)pentane-1,4-dione (1d)

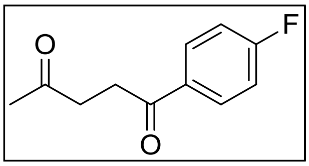

^{1}H NMR (400 MHz, $CDCl_3$) δ 8.03 – 7.96 (m, 2H), 7.16 – 7.08 (m, 2H), 3.25-3.22 (m, 2H), 2.88 (t, *J* = 6.3, 2H), 2.25 (s, 3H); ^{13}C NMR (101 MHz, $CDCl_3$) δ 207.4, 197.1, 166.0 (d, JC-F = 254.7 Hz), 133.3 (d, *J*C-F = 3Hz), 130.9 (d, *J*C-F = 9.4 Hz), 115.9 (d, *J*C-F = 21.9 Hz), 37.2, 32.5, 30.3.

1-(4-methoxyphenyl)pentane-1,4-dione (1e)

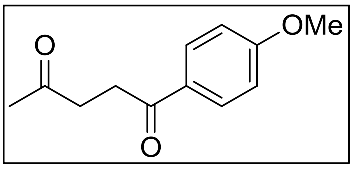

^{1}H NMR (400 MHz, $CDCl_3$) δ 7.98 – 7.92 (m, 2H), 6.96 – 6.89 (m, 2H), 3.86 (s, 3H), 3.25-3.20 (m, 2H), 2.86 (t, *J* = 6.3 Hz, 2H), 2.25 (s, 3H); ^{13}C NMR (101 MHz, $CDCl_3$) δ 207.8, 197.2, 163.7, 130.5, 129.9, 113.9, 55.7, 37.3, 32.3, 30.3.

1-phenylhexane-1,4-dione (1f)

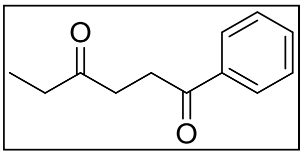

^{1}H NMR (400 MHz, $CDCl_3$) δ 8.01 – 7.94 (m, 2H), 7.58 – 7.53 (m, 1H), 7.49 – 7.42 (m, 2H), 3.31-3.36 (m, 2H), 2.85 (t, *J* = 6.2 Hz, 2H), 2.56 (q, *J* = 7.3 Hz, 2H), 1.09 (t, *J* = 7.3 Hz, 3H);^{13}C NMR (101 MHz, $CDCl_3$) δ 210.3, 198.9, 136.9, 133.3, 128.8, 128.2, 36.3, 36.0, 32.6, 8.0.

1-(p-tolyl)hexane-1,4-dione (1g)

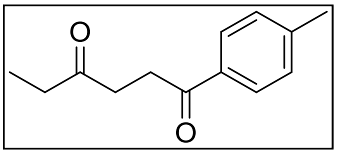

^{1}H NMR (400 MHz, $CDCl_3$) δ 7.90 (d, *J* = 8.2 Hz, 2H), 7.29 – 7.24 (m,2H), 3.31 – 3.24 (m, 2H), 2.86 (t, *J* = 6.3 Hz, 2H), 2.58 (q, *J* = 7.3 Hz, 2H), 2.42 (s, 3H), 1.11 (t, *J* = 7.3 Hz, 3H); ^{13}C NMR (101 MHz, $CDCl_3$) δ 210.5, 198.6, 144.1, 134.4, 129.5, 128.4, 36.3, 36.0, 32.5, 21.9, 8.1.

ω-TA-mediated reductive amination of 1,4-diketones 1a-g

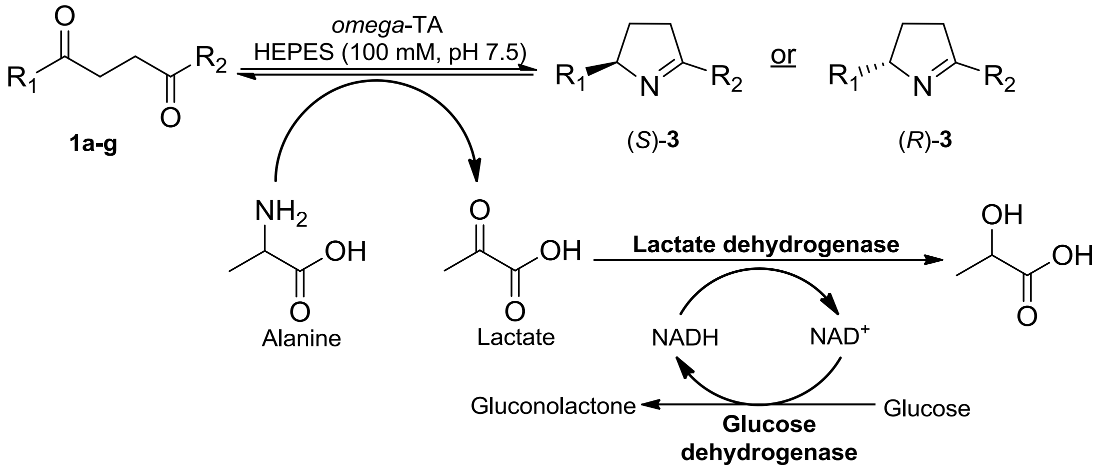

Analytical scale: Commercially available ATA113 or ATA117 (2.5 mg/mL) was rehydrated in HEPES buffer (2mL, 100 mM, pH 7.5) containing PLP (2.02 mM), NAD^{+} (1.5 mM), glucose (10 mg/mL, 55.5 mM), GDH (50 U), LDH (113 U) L-alanine (for ATA113, *pf*-ATA and *cv*-ATA) or D-alanine (for ATA117) (45 mg/mL, 500 mM). *pf*-ATA and *cv*-ATA were expressed as described above and 100 uL/mL of 'clarified cell extract' used. The pH of the mixture was adjusted to 7.5. The substrate (5 mM from a 200 mM stock in DMSO) was added and the mixture incubated at 30 ºC, 250 rpm in a shaking incubator. The reaction was monitored by GC-MS and GC-FID after 16 and 24 h following extraction of the basified* (pH 12 adjusted with 5 M NaOH) solution (100 uL) with EtOAc (1 x 300 uL).

**It should be noted that basification of reactions containing unreacted diketone leads to an intramolecular aldol reaction followed by dehydration of the resulting aldol product. The resulting α,β-unsaturated diketone can be detected in the GC-MS.*

Preparative scale: Commercially available ATA113 or ATA117 (2.5 mg/mL) was rehydrated in HEPES buffer (50 mL, 100 mM, pH 7.5) containing PLP (2.02 mM), NAD^{+} (1.5 mM), glucose (10 mg/mL, 55.5 mM), GDH

(50 U), LDH (113 U), L-alanine for ATA113 (45 mg/mL, 500 mM) or D-alanine for ATA117 (45 mg/mL, 500 mM). The pH of the mixture was adjusted to pH 7.5. Substrate **1a** (25 mM from a 1 M stock in DMSO) was added and the mixture incubated at 30 ºC, 250 rpm for 48h and monitored by GC-MS and GC-FID as detailed above. Upon completion of the reaction, the solution was centrifuged at 6000 rpm for 10 minutes. The pH of the supernatant was adjusted to 2 extracted with EtOAc (1 x 150 mL) to remove remaining diketone starting material. The pH of the solution was then adjusted to 12 and extracted with EtOAc (3 x 150 mL). The solvent was removed *in vacuo,* dissolved in Et_2O and washed with basic (pH 12) water to remove residual DMSO. The organic layer was dried over $MgSO_4$, filtered and the solvent was removed *in vacuo*. The resulting pyrrolines were filtered through a short pad of silica and eluted in cyclohexane/EtOAc (90/10). 120mg of (-)-(*S*)-**3a** (91% yield, >99% *ee*) and 80mg of (+)-(*R*)-**3a** (65% yield, >99% *ee*) was isolated.

NMR Data for Pyrrolines (*S*)-3a-g Isolated Following Preparative-scale Reductive Amination with ATA113

(*S*)-5-Methyl-2-phenyl-1-pyrroline (3a)

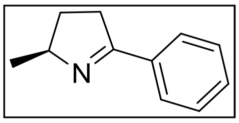

Light yellow oil (230 mg, 91% yield, >99% *ee*)^{1}H NMR (400 MHz, $CDCl_3$) δ 7.90 – 7.80 (m, 2H), 7.47 – 7.35 (m, 3H), 4.36 – 4.25 (m, 1H), 3.08 (dddd, *J* = 17.0, 9.9, 4.8, 2.1 Hz, 1H), 2.91 (dddd, *J* = 17.0, 9.6, 7.7, 1.8 Hz, 1H), 2.26 (dddd, *J* = 12.6, 9.8, 7.7, 4.8 Hz, 1H), 1.57 (dddd, *J* = 12.6, 9.9, 7.7, 6.7 Hz, 1H), 1.38 (d, *J* = 6.8 Hz, 3H); ^{13}C NMR (101 MHz, $CDCl_3$) δ 172.4, 134.5, 130.8, 128.7, 128.0, 68.4, 35.4, 30.7, 22.3; $[\alpha]_D^{20}$ = -110.5 (*c* = 2.0, $CHCl_3$, lit ref. 3).

(*R*)-5-Methyl-2-phenyl-1-pyrroline (3a)

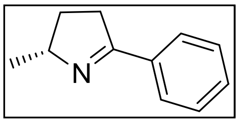

Light yellow oil (155 mg, 65% yield, >99% *ee*); $[\alpha]_D^{20}$ = +106.2 (*c* = 1.5, $CHCl_3$, lit ref. 3).

(*S*)-5-Methyl-2-(4-methylphenyl)-1-pyrroline (3b)

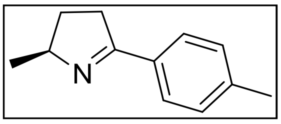

Yellow oil (223 mg, 86% yield).^{1}H NMR (400 MHz, $CDCl_3$) δ 7.73 (d, *J* = 8.1 Hz, 2H), 7.20 (d, *J* = 8.1 Hz, 2H), 4.32 – 4.21 (m, 1H), 3.04 (dddd, *J* = 17.2, 9.9, 4.8, 2.1 Hz, 1H), 2.86 (dddd, J = 17.2, 9.7, 7.9, 2.0 1H), 2.37 (s, 3H), 2.23 (dddd, *J* = 12.6, 9.8, 7.6, 4.8 Hz, 1H), 1.54 (dddd, *J* = 12.6, 9.8, 7.9, 6.7 Hz, 1H), 1.36 (d, *J* = 6.8 Hz, 3H);^{13}C NMR (101 MHz, $CDCl_3$) δ 171.9, 140.7, 132.1, 129.3, 127.8, 68.5, 35.4, 30.8, 22.4, 21.6; $[\alpha]_D^{20}$ = -77.4 (*c* = 0.8, $CHCl_3$, lit ref. 3).

(*S*)-5-Methyl-2-(4-chlorophenyl)-1-pyrroline (3c)

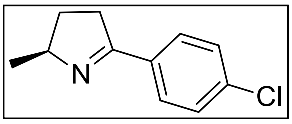

Yellow oil (243 mg, 84% yield).^{1}H NMR (400 MHz, $CDCl_3$) δ 7.79 (d, *J* = 8.5 Hz, 2H), 7.41 – 7.34 (m, 2H), 4.36 – 4.22 (m, 1H), 3.04 (dddd, *J* = 17.0, 9.9, 4.7, 2.1 Hz, 1H), 2.87 (dddd, *J* = 17.0, 9.7, 7.7, 1.8 Hz, 1H), 2.27 (dddd, *J* = 12.6, 9.9, 7.7, 4.7 Hz, 1H), 1.66 – 1.50 (m, 1H), 1.37 (d, *J* = 6.8 Hz, 3H); ^{13}C NMR (101 MHz, $CDCl_3$) δ 171.0, 136.5, 133.3, 129.2, 128.8, 68.7, 35.4, 30.9, 22.3.

(*S*)-5-Methyl-2-(4-fluorophenyl)-1-pyrroline (3d)

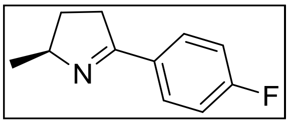

Yellow oil (215 mg, 81% yield).^{1}H NMR (400 MHz, $CDCl_3$) δ 7.87 – 7.79 (m, 2H), 7.12 – 7.03 (m, 2H), 4.32 – 4.21 (m, 1H), 3.03 (dddd, *J* = 17.0, 9.8, 4.7, 2.1 Hz, 1H), 2.86 (dddd, *J* = 17.0, 9.6, 7.6, 1.6, 1H), 2.25 (dddd, *J* = 12.5, 9.8, 7.6, 4.7 Hz, 1H), 1.61 – 1.51 (m, 1H), 1.36 (d, *J* = 6.8 Hz, 3H); ^{13}C NMR (101 MHz, $CDCl_3$) δ 170.9, 164.3 (d, *J* = 248.7 Hz),131.2 (d, *J* = 3Hz,), 130.0 (d, *J* = 8.6Hz), 115.6 (d, *J* = 21.7 Hz), 68.6, 35.5, 30.9, 22.3.

(*S*)-5-Methyl-2-(4-methoxyphenyl)-1-pyrroline (3e)

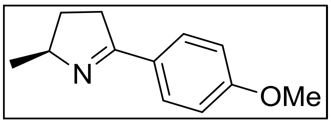

Yellow oil (250 mg, 88% yield).^{1}H NMR (400 MHz, $CDCl_3$) δ 7.83 – 7.76 (m, 2H), 6.94 – 6.87 (m, 2H), 4.31 – 4.20 (m, 1H), 3.84 (s, 3H), 3.03 (dddd, *J* = 17.0, 9.8, 4.7, 2.0 Hz, 1H), 2.85 (dddd, J = 17.0, 9.8, 7.6, 1.6, 1H), 2.23 (dddd, *J* = 12.5, 9.8, 7.6, 4.8 Hz, 1H), 1.59 – 1.48 (m, 1H), 1.35 (d, *J* = 6.8 Hz, 3H); ^{13}C NMR (101 MHz, $CDCl_3$) δ 171.4, 161.6, 129.5, 113.9, 68.3, 55.5, 35.3, 30.9, 22.4.

(*S*)-5-Ethyl-2-phenyl-1-pyrroline (3f)

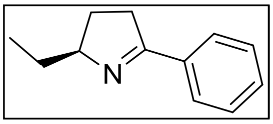

Yellow oil (130 mg, 50% yield).^{1}H NMR (400 MHz, $CDCl_3$) δ 7.88 - 7.83 (m, 2H), 7.46 – 7.37 (m, 3H), 4.19 – 4.08 (m, 1H), 3.02 (dddd, *J* = 17.0, 10.0, 4.9, 2.2 Hz, 1H), 2.89 (dddd, *J* = 17.0, 9.7, 7.6, 1.8 Hz, 1H), 2.20 (dddd, *J* = 12.8, 9.7, 7.8, 4.9 Hz, 1H), 1.95 – 1.82 (m, 1H), 1.62 (dddd, J = 12.8, 10.0, 7.6, 6.8 Hz, 1H), 1.59-1.48 (m, 1H), 1.02 (t, *J* = 7.4 Hz, 3H);^{13}C NMR (101 MHz, $CDCl_3$) δ 172.0, 134.7, 130.3, 128.4, 127.7, 74.6, 35.0, 29.4, 28.0, 10.9; $[\alpha]_D^{20}$ = -67.6 (*c* = 0.5, $CHCl_3$, lit ref. 3).

(*S*)-5-Ethyl-2-(4-methylphenyl)-1-pyrroline (3g)

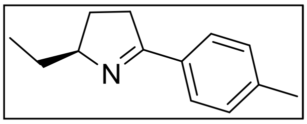

Yellow oil (225 mg, 80% yield).^{1}H NMR (400 MHz, $CDCl_3$) δ 7.74 (d, *J* = 8.0 Hz, 2H), 7.20 (d, *J* = 8.0 Hz, 2H), 4.17 – 4.07 (m, 1H), 3.00 (dddd, *J* = 17.0, 10.0, 4.9, 2.2 Hz, 1H), 2.86 (dddd, *J* = 17.0, 9.6, 7.6, 2.0Hz, 1H), 2.38 (s, 3H), 2.18 (dddd, *J* = 12.7, 10.0, 7.8, 4.9 Hz, 1H), 1.94 – 1.82 (m, 1H), 1.60 (dddd, J = 12.7, 10.0, 7.6, 6.8 1H), 1.58 – 1.47 (m, 1H), 1.01 (t, *J* = 7.4 Hz, 3H); ^{13}C NMR (101 MHz, $CDCl_3$) δ 172.1, 140.7, 132.1, 129.3, 127.9, 74.6, 35.2, 29.6, 28.1, 21.7, 11.1.

Preparation of (2*S*,5*S*)- and (2*S*,5*R*)-2,5-pyrrolidines as standards

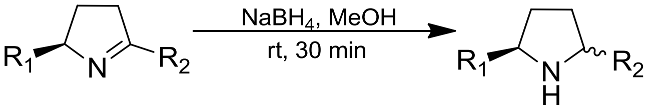

(-)-(*S*)-3a-g

mixture of (2*S*,5*S*)- and (2*S*,5*R*)-**4**

Pyrrolines **3a-g** (0.625 mmol) were dissolved in MeOH (7 mL) and $NaBH_4$ (95 mg, 2.5 mmol) was added at 0 ºC. The solution was stirred at room temperature for 30 minutes after which 7 mL of H_2O was added and the organic solvent was removed *in vacuo*. The pH of the solution was adjusted to 12 and extracted with EtOAc (2 x

10 mL). The organic fractions were combined and dried over $MgSO_4$, filtered and the solvent was evaporated *in vacuo*. The resulting amines were not purified further.

(*S*)-2-methyl-5-phenylpyrrolidine

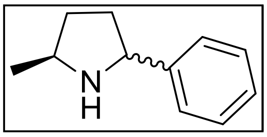

^{1}H NMR (400 MHz, $CDCl_3$) δ 7.44 – 7.15 (m, 10H), 4.37 (t, *J* = 7.6 Hz, 1H), 4.16 (t, *J* = 7.9 Hz, 1H), 3.57 – 3.46 (m, 1H), 3.36 – 3.25 (m, 1H), 2.67 (br. s, 2H), 2.34 – 2.24 (m, 1H), 2.23 – 2.05 (m, 2H), 2.05 – 1.92 (m, 1H), 1.85 – 1.70 (m, 2H), 1.52 – 1.39 (m, 2H), 1.25 (d, *J* = 6.2 Hz, 3H), 1.21 (d, *J* = 6.2 Hz, 3H); ^{13}C NMR (101 MHz, $CDCl_3$) δ 145.6, 144.7, 128.6, 128.5, 127.0, 126.9, 126.8, 126.6, 63.4, 61.8, 55.0, 54.4, 35.6, 34.9, 34.2, 33.7, 22.3, 21.7.

(S)-2-methyl-5-(p-tolyl)pyrrolidine

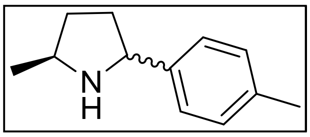

^{1}H NMR (400 MHz, $CDCl_3$) δ 7.29 – 7.10 (m, 8H), 4.33 (t, *J* = 7.6 Hz, 1H), 4.11 (t, *J* = 7.9 Hz, 1H), 3.57 – 3.47 (m, 1H), 3.34 – 3.23 (m, 1H), 2.34 – 2.21 (m, 9H), 2.20 – 2.04 (m, 2H), 1.98 (ddd, *J* = 13.0, 10.6, 6.6 Hz, 1H), 1.75 (m, 2H), 1.51 – 1.38 (m, 2H), 1.24 (d, *J* = 6.2 Hz, 3H), 1.20 (d, *J* = 6.3 Hz, 3H); ^{13}C NMR (101 MHz, $CDCl_3$) δ 142.6, 141.7, 136.6, 136.4, 129.3, 129.2, 126.8, 126.5, 63.0, 61.5, 55.0, 54.3, 35.6, 34.9, 34.3, 33.8, 22.4, 21.7, 21.3, 21.2.

(S)-2-(4-methoxyphenyl)-5-methylpyrrolidine

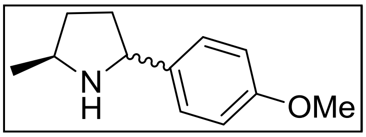

^{1}H NMR (400 MHz, $CDCl_3$) δ 7.32 – 7.23 (m, 4H), 6.88 – 6.82 (m, 4H), 4.33 – 4.28 (m, 1H), 4.10 (t, *J* = 8.0 Hz, 1H), 3.79 (s, 6H), 3.56 – 3.46 (m, 1H), 3.33 – 3.22 (m, 1H), 2.62 (br. s, 2H), 2.28 – 2.19 (m, 1H), 2.18 – 2.05 (m, 2H), 2.03 – 1.93 (m, 1H), 1.82 – 1.68 (m, 2H), 1.50 – 1.39 (m, 2H), 1.24 (d, *J* = 6.2 Hz, 3H), 1.20 (d, *J* = 6.3 Hz, 3H); ^{13}C NMR (101 MHz, $CDCl_3$) δ 158.7, 158.7, 137.3, 136.6, 127.9, 127.8, 114.0, 113.9, 62.7, 62.0, 55.5 (2 x C), 54.9, 54.2, 35.4, 34.8, 34.1, 33.7, 22.2, 21.7.

(S)-2-(4-chlorophenyl)-5-methylpyrrolidine

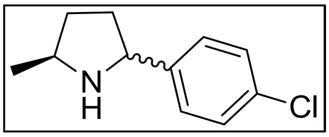

^{1}H NMR (400 MHz, $CDCl_3$) δ 7.34 – 7.21 (m, 8H), 4.33 (t, J=7.6 Hz, 1H), 4.12 (t, *J* = 7.9 Hz, 1H), 3.53 – 3.43 (m, 1H), 3.34 – 3.25 (m, 1H), 2.33 – 2.22 (m, 1H), 2.20 – 2.02 (m, 4H), 1.97 (dddd, *J* = 12.7, 8.7, 7.1, 5.9 Hz, 1H), 1.75 – 1.60 (m, 2H), 1.50 – 1.36 (m, 2H), 1.23 (d, *J* = 6.2 Hz, 3H), 1.21 (d, *J* = 6.3 Hz, 3H); ^{13}C NMR (101 MHz, $CDCl_3$) δ 144.5, 143.7, 132.5, 132.4, 128.6, 128.6, 128.2, 128.0, 62.3, 61.1, 54.8, 54.4, 35.8, 35.0, 34.4, 33.5, 22.3, 21.9.

(S)-2-(4-fluorophenyl)-5-methylpyrrolidine

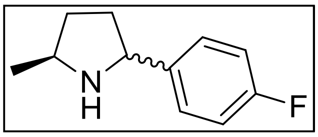

^{1}H NMR (400 MHz, $CDCl_3$) δ 7.37 – 7.27 (m, 4H), 7.02 – 6.94 (m, 4H), 4.33 (t, *J* = 7.6 Hz, 1H), 4.11 (t, 8 Hz, 1H), 3.53 – 3.43 (m, 1H), 3.34 – 3.23 (m, 1H), 2.31 – 2.20 (m, 1H), 2.20 – 2.03 (m,

2H), 1.97 (dddd, J = 12.7, 8.7, 7.1, 5.9 Hz, 1H), 1.78 – 1.62 (m, 2H), 1.50 – 1.35 (m, 2H), 1.23 (d, J = 6.2 Hz, 3H), 1.20 (d, J = 6.3 Hz, 3H); ^{13}C NMR (101 MHz, $CDCl_3$) δ 163.1 (d, J = 8.6 Hz), 160.7 (d, J = 8.5 Hz), 141.7 (d, J = 3 Hz), 140.7 (d, J = 3 Hz), 128.3 (d, J = 7.8 Hz), 128.0 (d, J = 7.9 Hz), 115.2 (d, J = 21 Hz), 115.2 (d, J = 21 Hz), 62.4, 61.1, 54.8, 54.3, 35.9, 35.0, 34.4, 33.5, 22.4, 21.9.

(S)-2-ethyl-5-phenylpyrrolidine

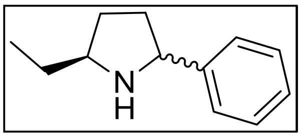

^{1}H NMR (400 MHz, $CDCl_3$) δ 7.41 – 7.28 (m, 8H), 7.25 – 7.19 (m, 2H), 4.31 (dd, J = 8.0, 7.0 Hz, 1H), 4.18 – 4.11 (t, 4.13, J =8Hz, 1H), 3.35 – 3.25 (m, 1H), 3.15 – 3.03 (m, 1H), 2.30 – 2.21 (m, 4H), 2.20 – 2.03 (m, 1H), 1.97 (m, 2H), 1.83 – 1.63 (m, 1H), 1.63 – 1.41 (m, 6H), 0.97 (t, J = 8.0 Hz, 3H), 0.95 (t, J = 8.0 Hz, 3H); ^{13}C NMR (101 MHz, $CDCl_3$) δ 146.0, 145.1, 128.6, 129.0, 127.0, 126.9, 126.9, 126.6, 62.8, 61.7, 61.2, 60.7, 35.4, 34.0, 32.7, 31.4, 30.2, 29.8, 11.8, 11.7.

(S)-2-ethyl-5-(p-tolyl)pyrrolidine

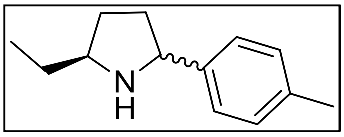

^{1}H NMR (400 MHz, $CDCl_3$) δ 7.26 (m, 4H), 7.13 (d, J = 7.9 Hz, 4H), 4.32 – 4.24 (m, 1H), 4.12 (t, J = 7.9 Hz, 1H), 3.35 – 3.25 (m, 1H), 3.15 – 3.05 (m, 1H), 2.83 (br. s, 2H), 2.33 (s, 6H), 2.27 – 2.19 (m, 1H), 2.18 – 2.06 (m, 2H), 2.02 – 1.92 (m, 1H), 1.83 – 1.65 (m, 2H), 1.65 – 1.40 (m, 6H), 0.97 (t, J = 8.0 Hz, 3H), 0.93 (t, J = 8.0 Hz, 3H); ^{13}C NMR (101 MHz, $CDCl_3$) δ 142.2, 141.5, 136.6, 129.3, 129.2, 126.8, 126.6, 62.6, 61.5, 61.2, 60.7, 35.1, 33.9, 32.5, 31.4, 29.9, 29.6, 21.3, 11.8, 11.7.

Analytical scale deracemisations with MAO-N variants

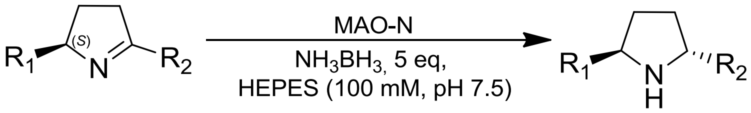

(*S*)-3a-g **(2*S*,5*R*)-4a-g**

The MAO-N genes corresponding to the D5 and D9 variants were expressed as previously reported.[4] Cells expressing the MAO-N D5 or D9 variant (50 mg/mL) were re-suspended in HEPES buffer (2mL, 100 mM, pH 7.5). The substrate (5 mM of a 200 mM stock in DMSO) was added along with NH_3BH_3 (25 mM, 5eq) and the mixture was incubated at 30 ºC, 250 rpm in a shaking incubator for 24-48h. The reaction was monitored by GC-MS and GC-FID over time, followed by extraction of the basified (pH 12 adjusted with 5 M NaOH) solution (100 uL) with EtOAc (1 x 300 uL), providing (2*S*,5*R*)-**4a-g**. The same procedure was followed for the (+)-(*R*)-**3a** enantiomer providing the (2*R*,5*R*)-**4a**.

One-pot transaminase/MAO-N cascade for the preparative scale synthesis of (2*S*,5*R*)-4a

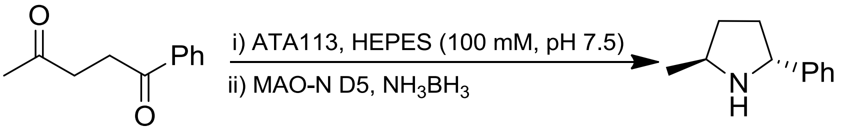

ATA113 (125 mg) was rehydrated in HEPES buffer (50 mL, 100 mM, pH 7.5) containing PLP (2.02 mM), NAD^+ (1.5 mM), glucose (500 mg, 55.5 mM), GDH (50 U), LDH (113 U), L-alanine (4.5 g, 500 mM). The pH of the mixture was adjusted to 7.5. Diketone **1a** (220 mg, 1.25 mmol, 25 mM) in DMSO (1.25 mL from a 1 M stock in DMSO) was added and the mixture incubated at 30 ºC and 250 rpm in a shaking incubator for 24h. The reaction was monitored by GC-MS and GC-FID as detailed above and upon completion of the reaction, wet cells expressing the MAO-N D5 variant (50 mg/mL) were added directly along with NH_3BH_3 (25 mM, 5eq) and the mixture was incubated at 30 ºC, 250 rpm for 24h following which, the solution was centrifuged at 6000 rpm for 10 minutes. The supernatant was adjusted to pH 2 and extracted with EtOAc (1 x 150 mL) to remove any remaining diketone starting material. The solution was then adjusted to pH 12 and extracted with EtOAc (3 x 150 mL). The solvent was removed *in vacuo*, dissolved in Et_2O and washed with basified (pH 2) water to remove residual DMSO. The organic layer was dried over $MgSO_4$, filtered and the solvent was removed *in vacuo*. The resulting pyrrolidine was filtered through a short pad of silica and eluted in cyclohexane/EtOAc (90/10) and isolated as a clear oil (82% yield, >99% *ee*). ^{1}H NMR (400 MHz, $CDCl_3$) δ 7.43 – 7.28 (m, 4H), 7.23 (m, 1H), 4.38 (t, *J*=7.6, 1H), 3.55 (m, 1H), 2.35 – 2.22 (m, 2H), 2.12 (m, 1H), 1.88 – 1.75 (m, 1H), 1.23 (d, *J* = 6.3 Hz, 3H).

The one-pot procedure was also carried out with diketones **1b**, **1d** and **1e** (with MAO-N D9 variant) on an analytical scale (2 mL, 5mM substrate) providing (2*S*,5*R*)-**4b**, (2*S*,5*R*)-**4d** and (2*S*,5*R*)-**4e** in >99% conversion and >99% *ee*.

NMR Spectra of ATA113 Reductive Amination Products (*S*)-3a-g

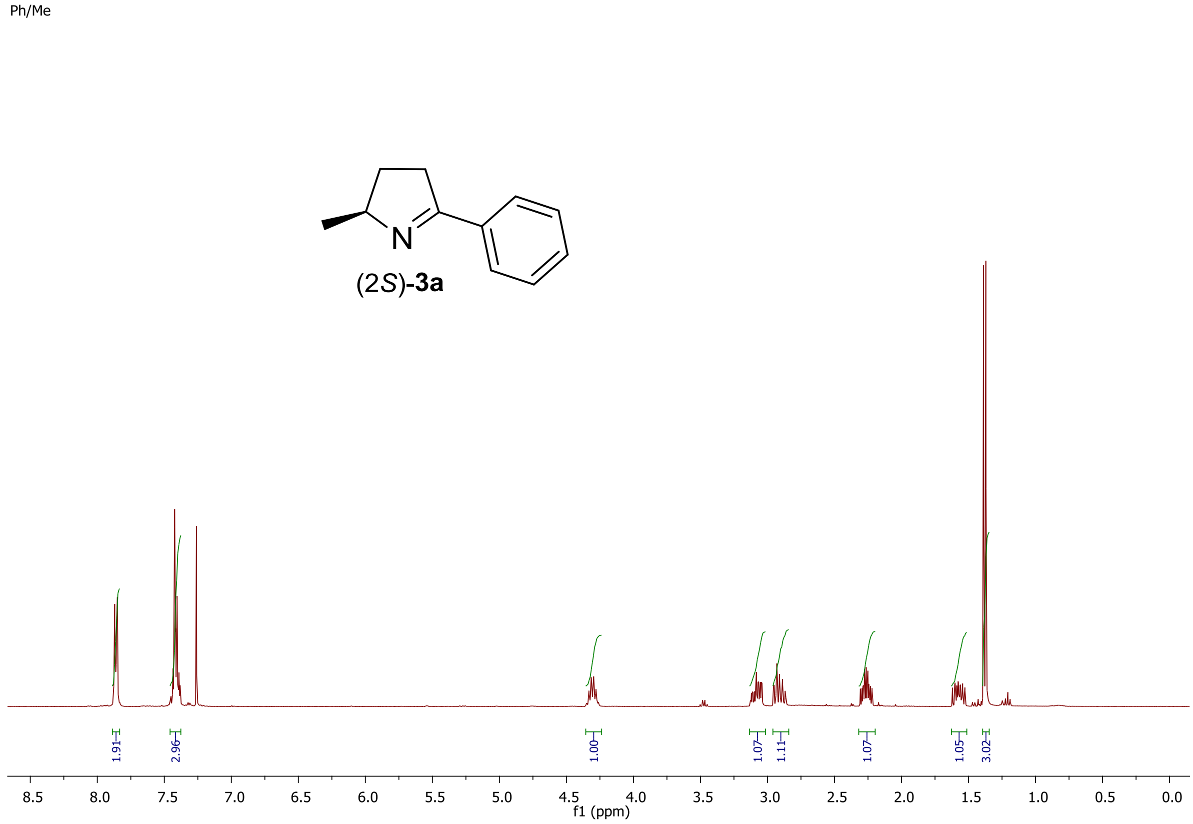

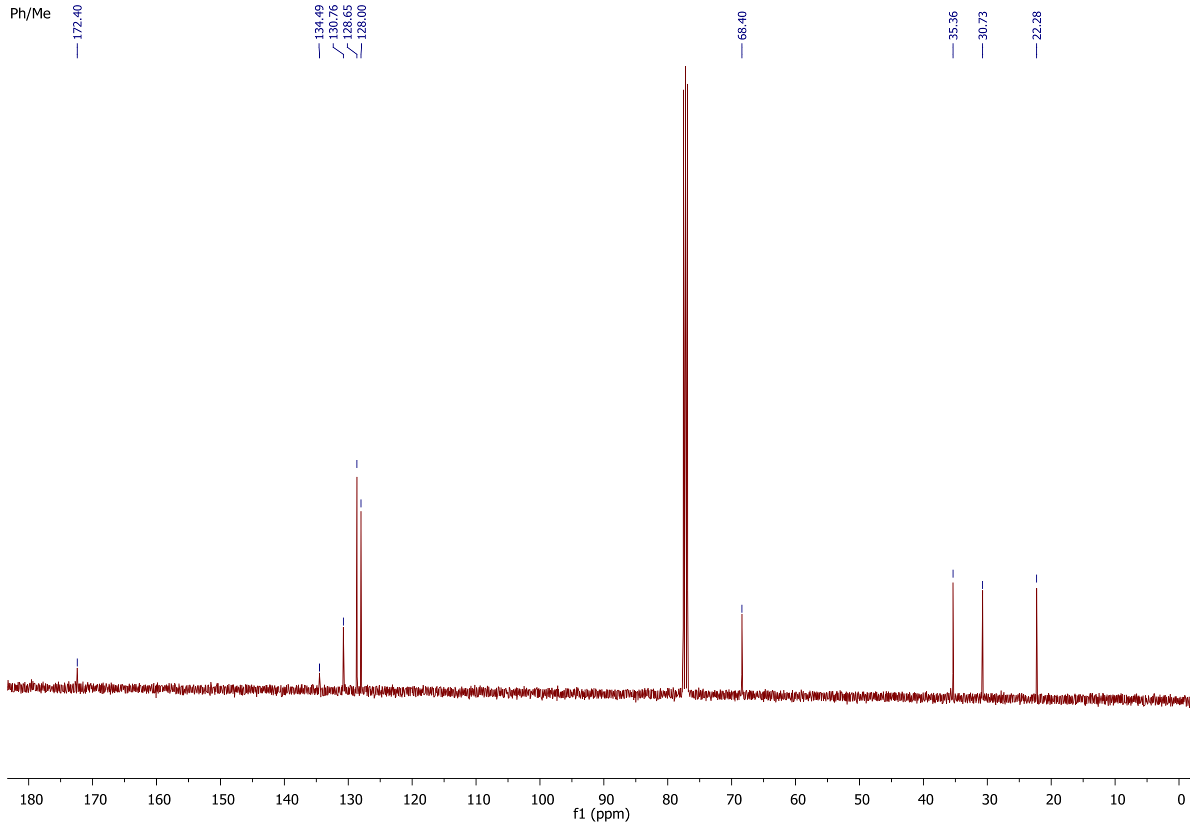

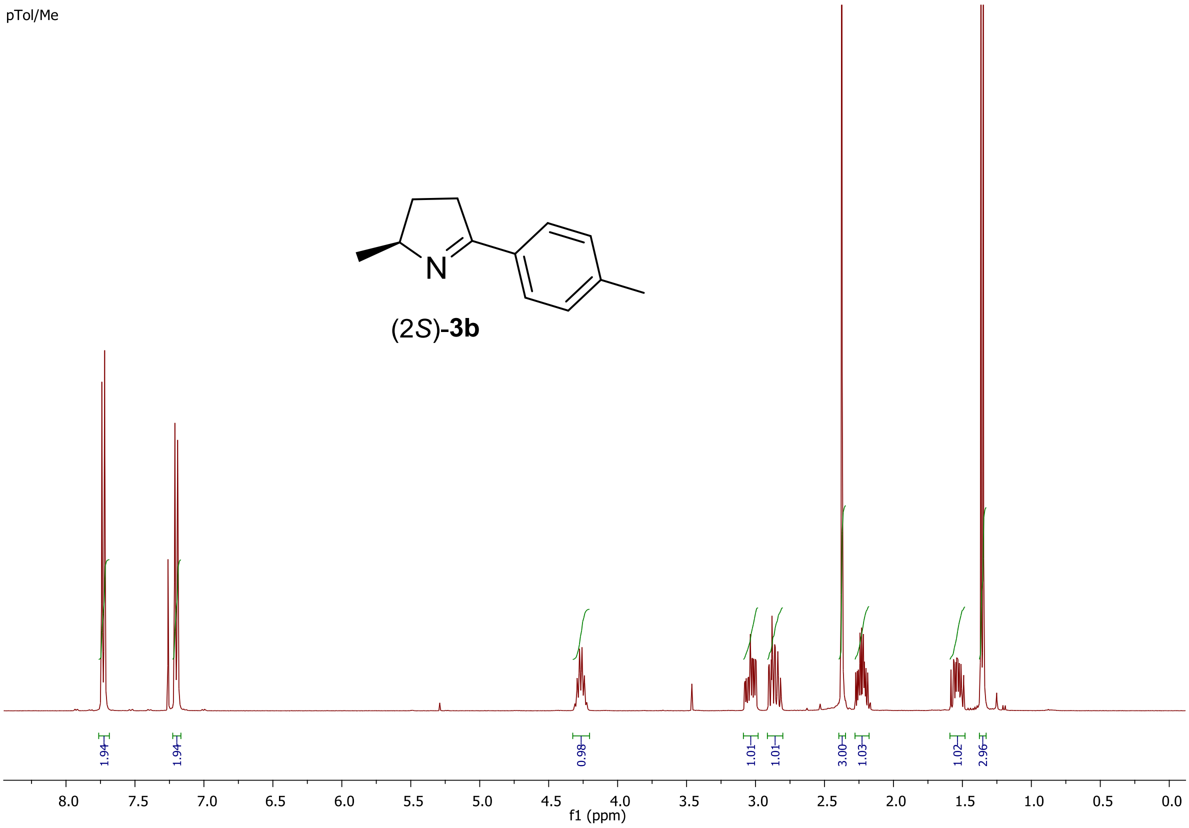
pTol/Me
N
(2S)-3b
1.94
1.94
0.98
1.01
1.01
3.00
1.03
1.02
2.96
8.0 7.5 7.0 6.5 6.0 5.5 5.0 4.5 4.0 3.5 3.0 2.5 2.0 1.5 1.0 0.5 0.0
f1 (ppm)

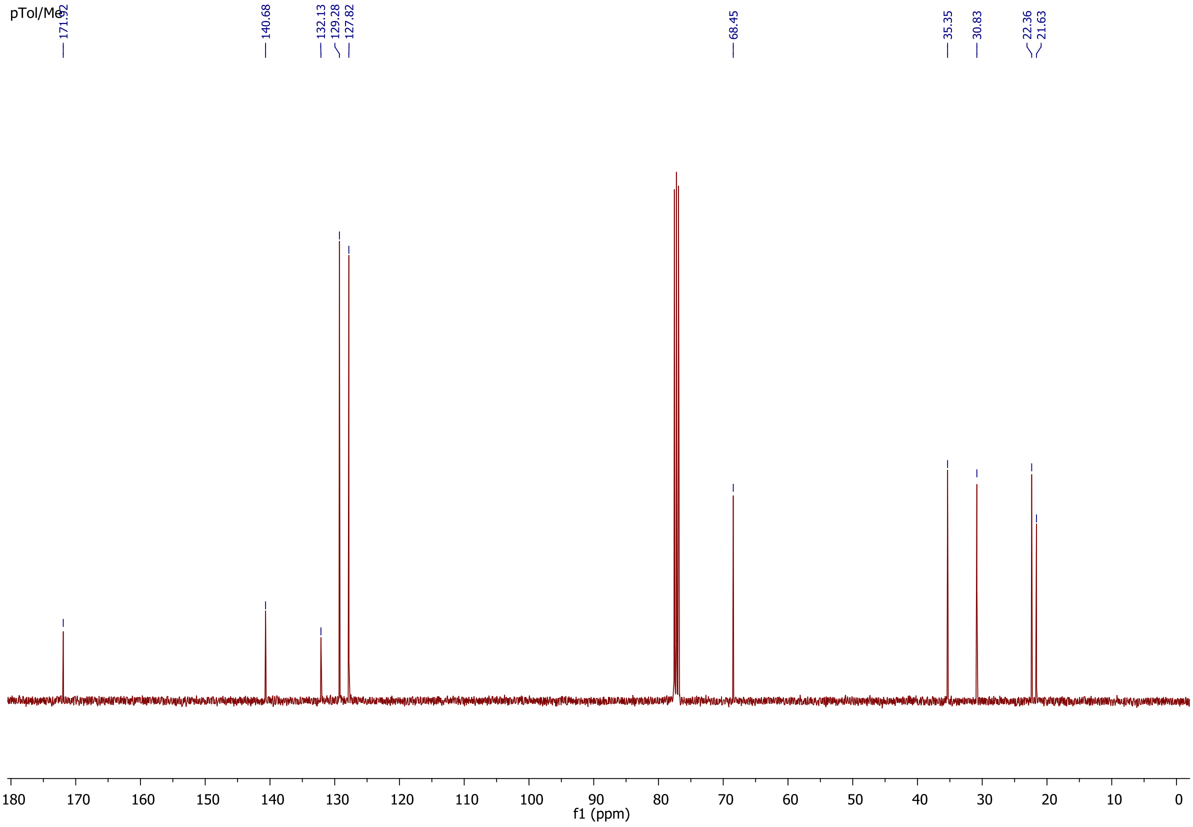
pTol/Me
171.92
140.68
132.13
129.28
127.82
68.45
35.35
30.83
22.36
21.63
180 170 160 150 140 130 120 110 100 90 80 70 60 50 40 30 20 10 0
f1 (ppm)

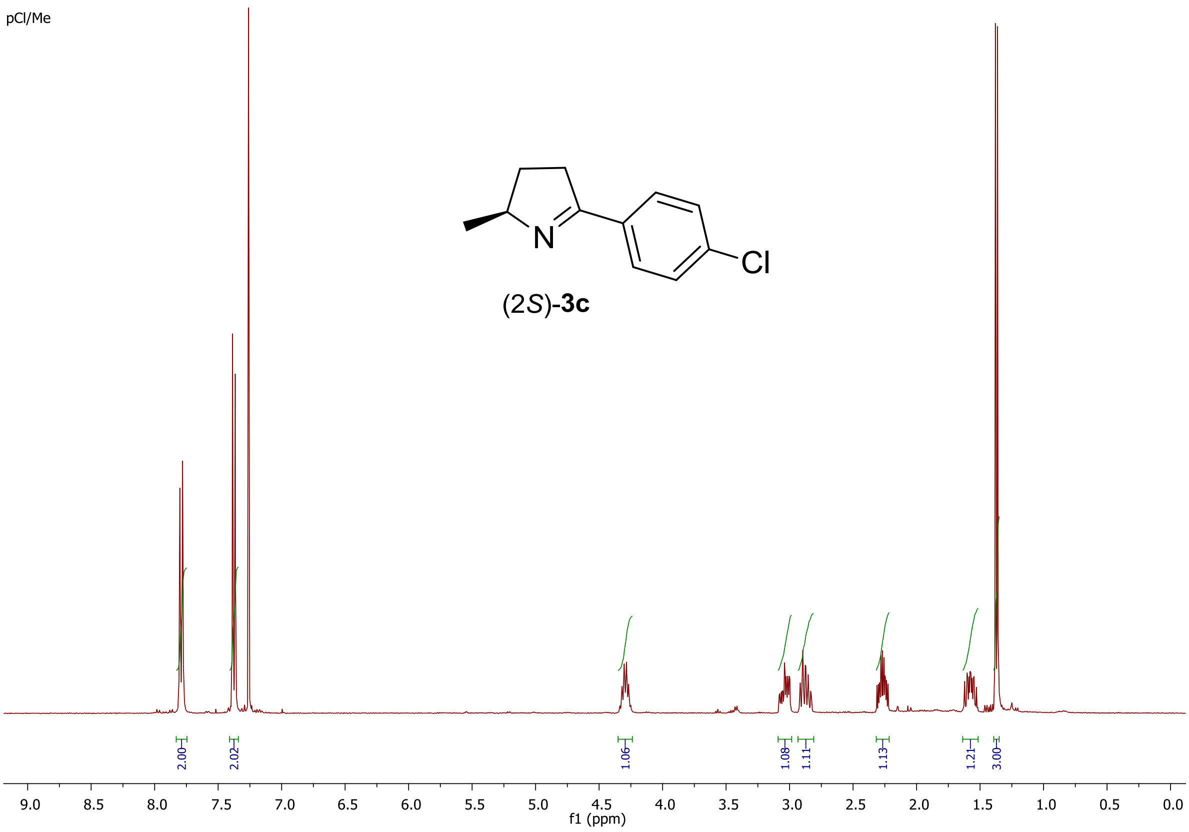
pCl/Me
N
Cl
(2S)-3c
2.00
2.02
1.06
1.08
1.11
1.13
1.21
3.00
f1 (ppm)

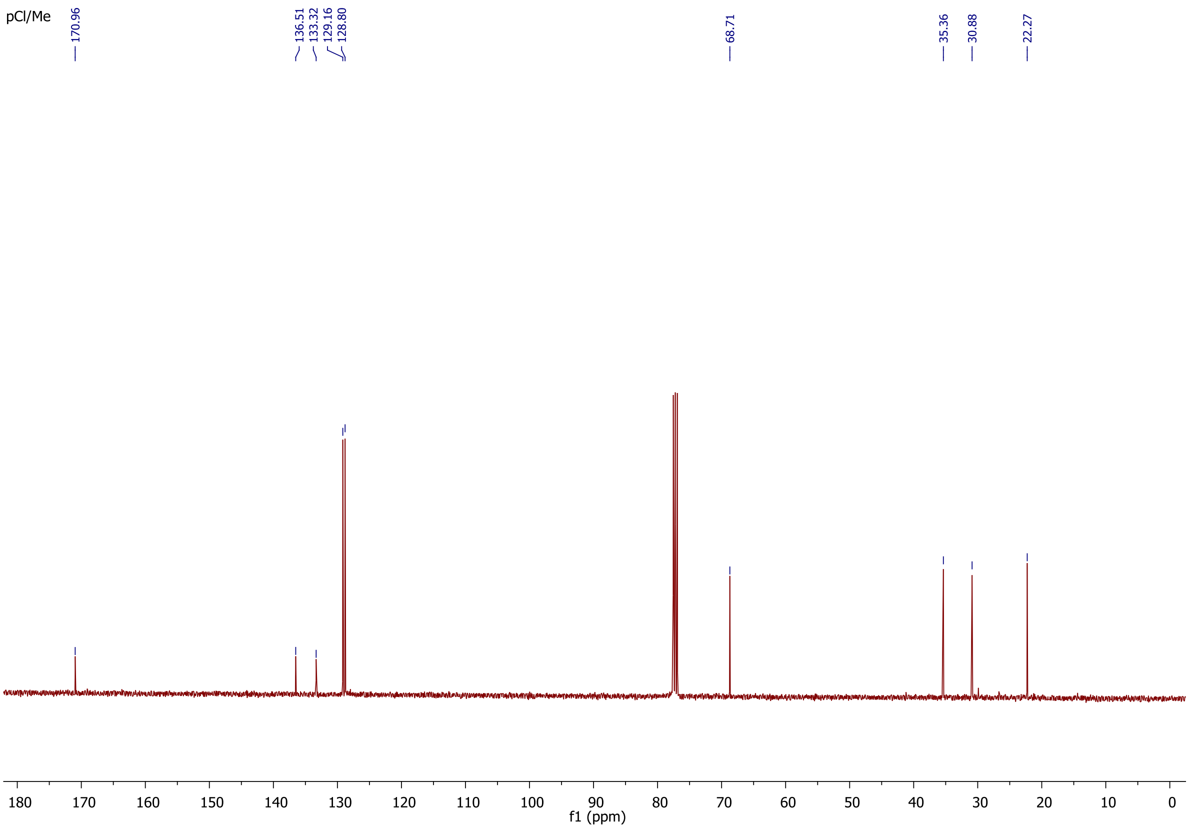
pCl/Me
170.96
136.51
133.32
129.16
128.80
68.71
35.36
30.88
22.27
f1 (ppm)

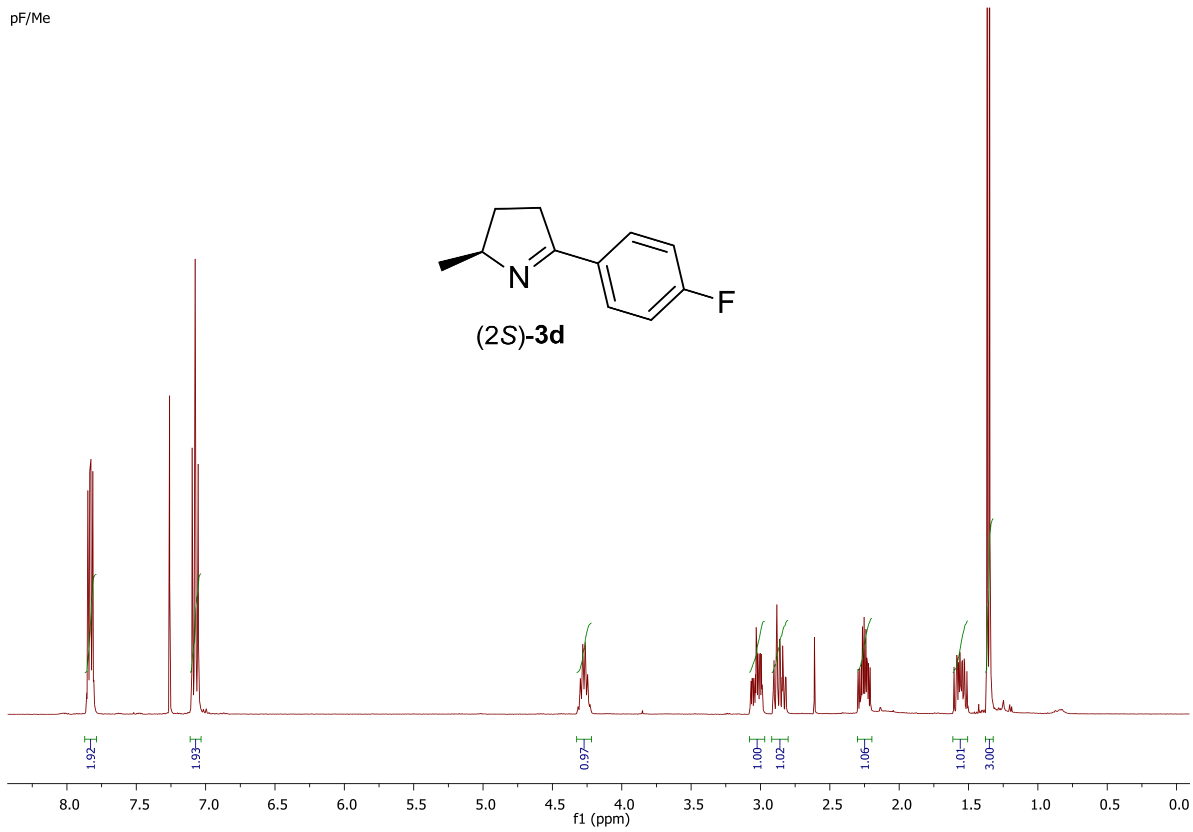
pF/Me
(2*S*)-**3d**
N
F
1.92
1.93
0.97
1.00
1.02
1.06
1.01
3.00
8.0
7.5
7.0
6.5
6.0
5.5
5.0
4.5
4.0
3.5
3.0
2.5
2.0
1.5
1.0
0.5
0.0
f1 (ppm)

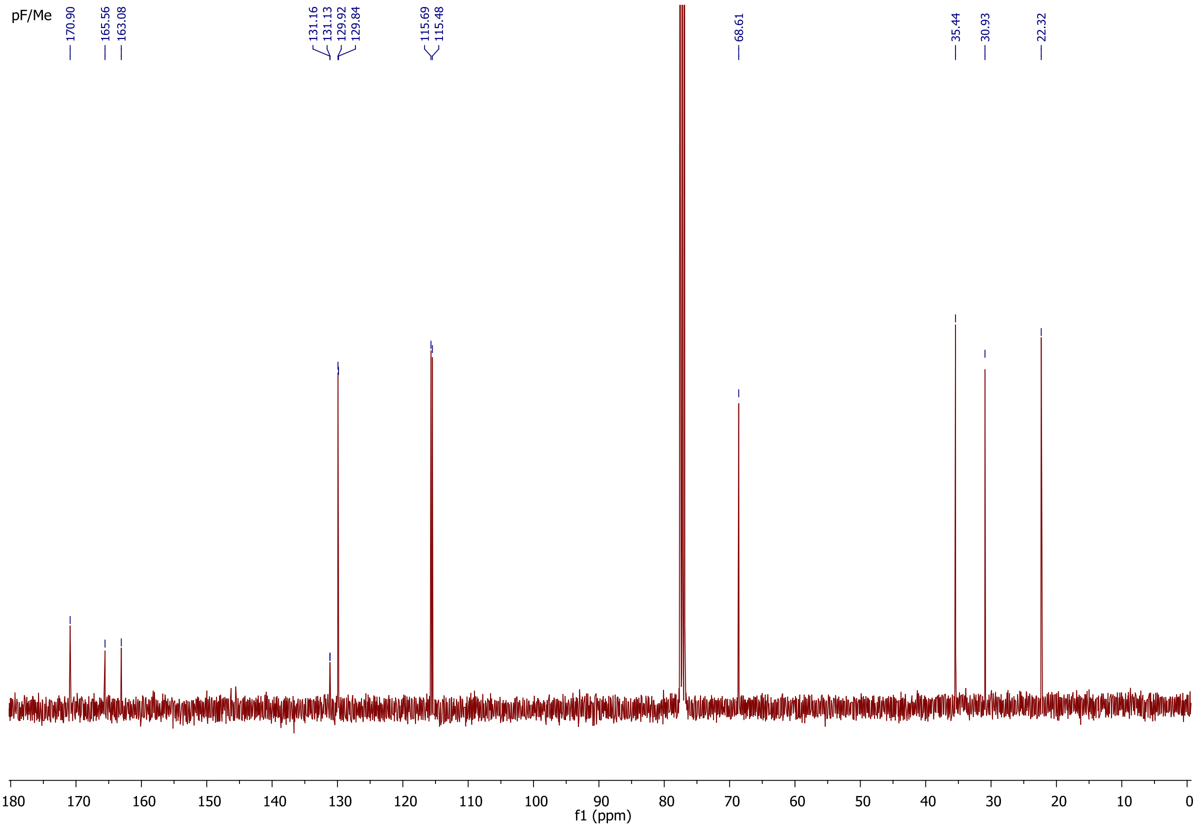
pF/Me
170.90
165.56
163.08
131.16
131.13
129.92
129.84
115.69
115.48
68.61
35.44
30.93
22.32
180
170
160
150
140
130
120
110
100
90
80
70
60
50
40
30
20
10
0
f1 (ppm)

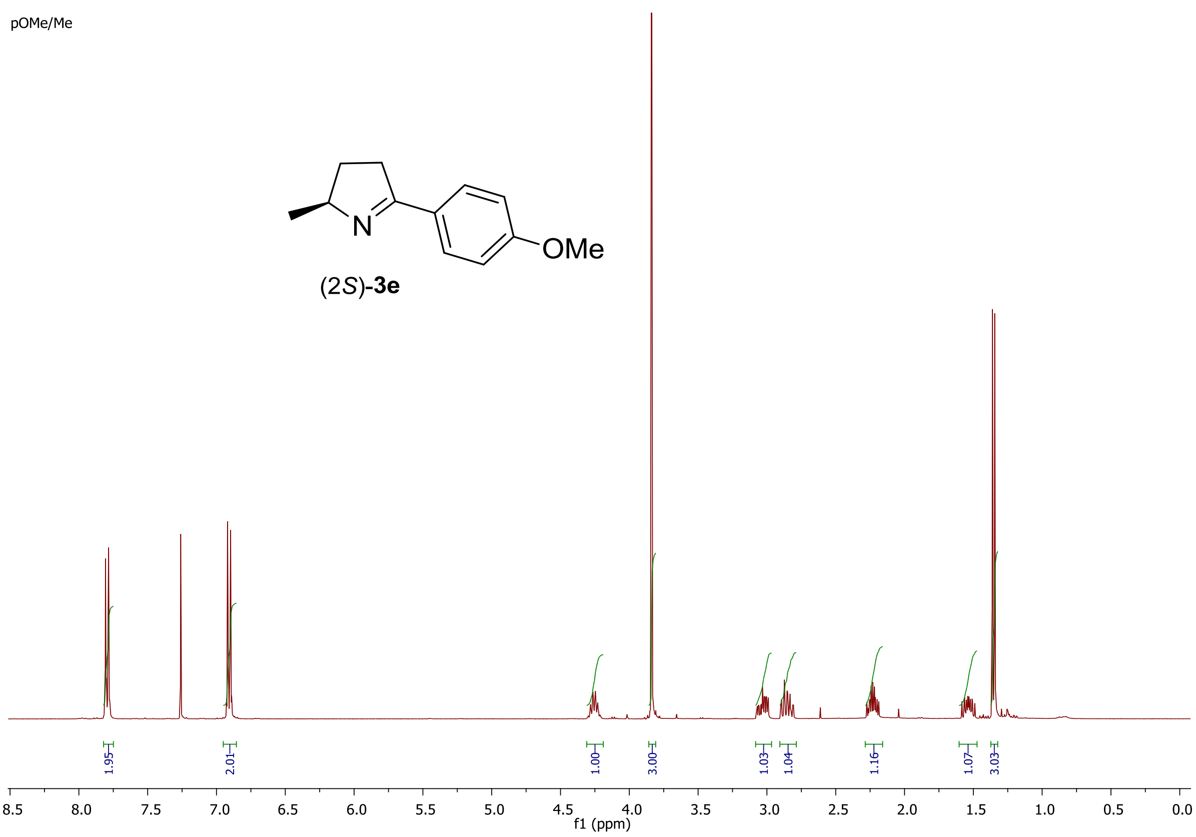
pOMe/Me
N
OMe
(2S)-3e
1.95
2.01
1.00
3.00
1.03
1.04
1.16
1.07
3.03
8.5 8.0 7.5 7.0 6.5 6.0 5.5 5.0 4.5 4.0 3.5 3.0 2.5 2.0 1.5 1.0 0.5 0.0
f1 (ppm)

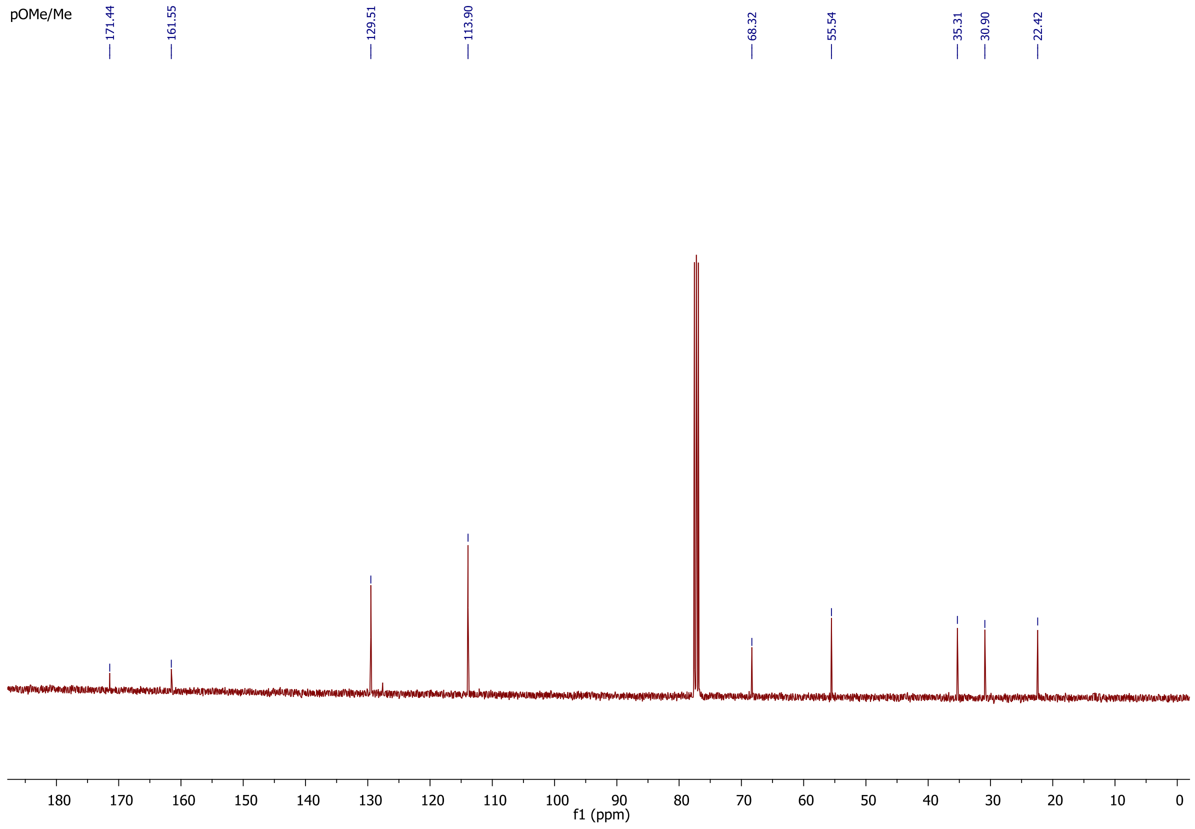
pOMe/Me
171.44
161.55
129.51
113.90
68.32
55.54
35.31
30.90
22.42
180 170 160 150 140 130 120 110 100 90 80 70 60 50 40 30 20 10 0
f1 (ppm)

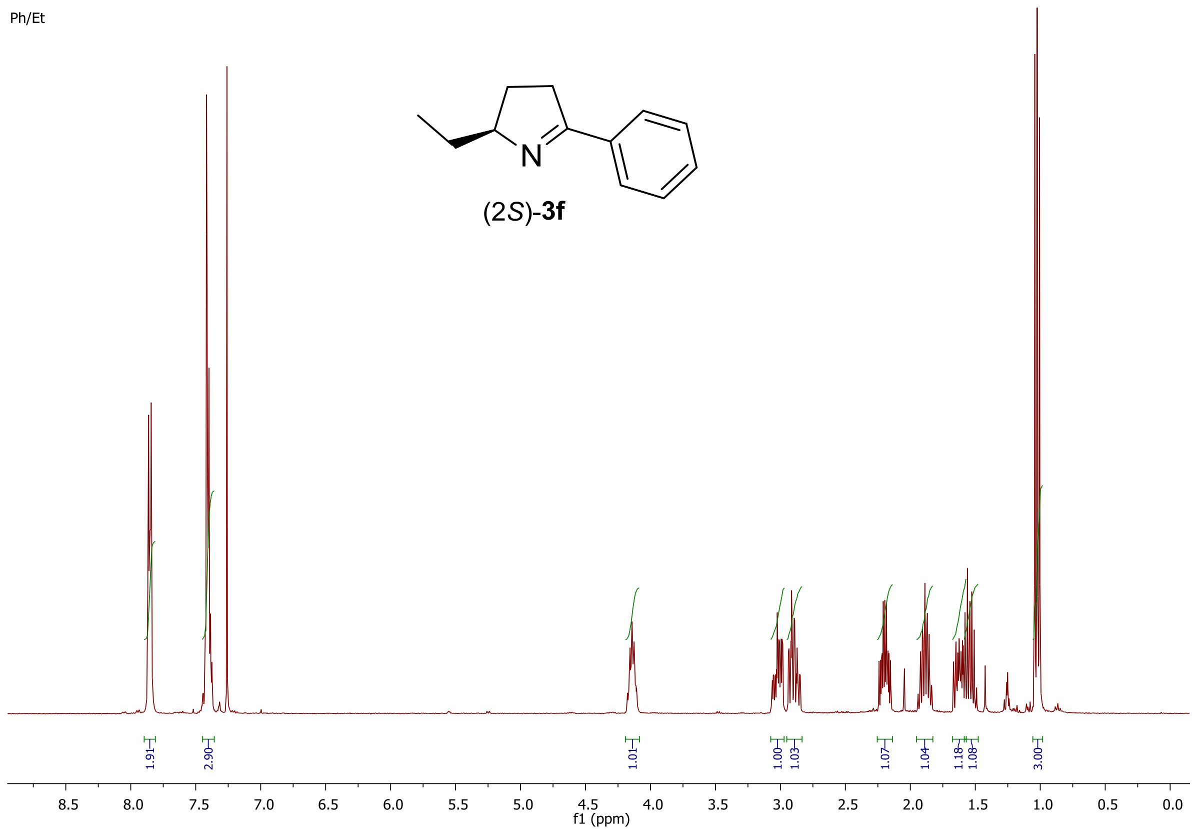
Ph/Et
N
(2*S*)-**3f**
1.91
2.90
1.01
1.00
1.03
1.07
1.04
1.18
1.08
3.00
8.5 8.0 7.5 7.0 6.5 6.0 5.5 5.0 4.5 4.0 3.5 3.0 2.5 2.0 1.5 1.0 0.5 0.0
f1 (ppm)

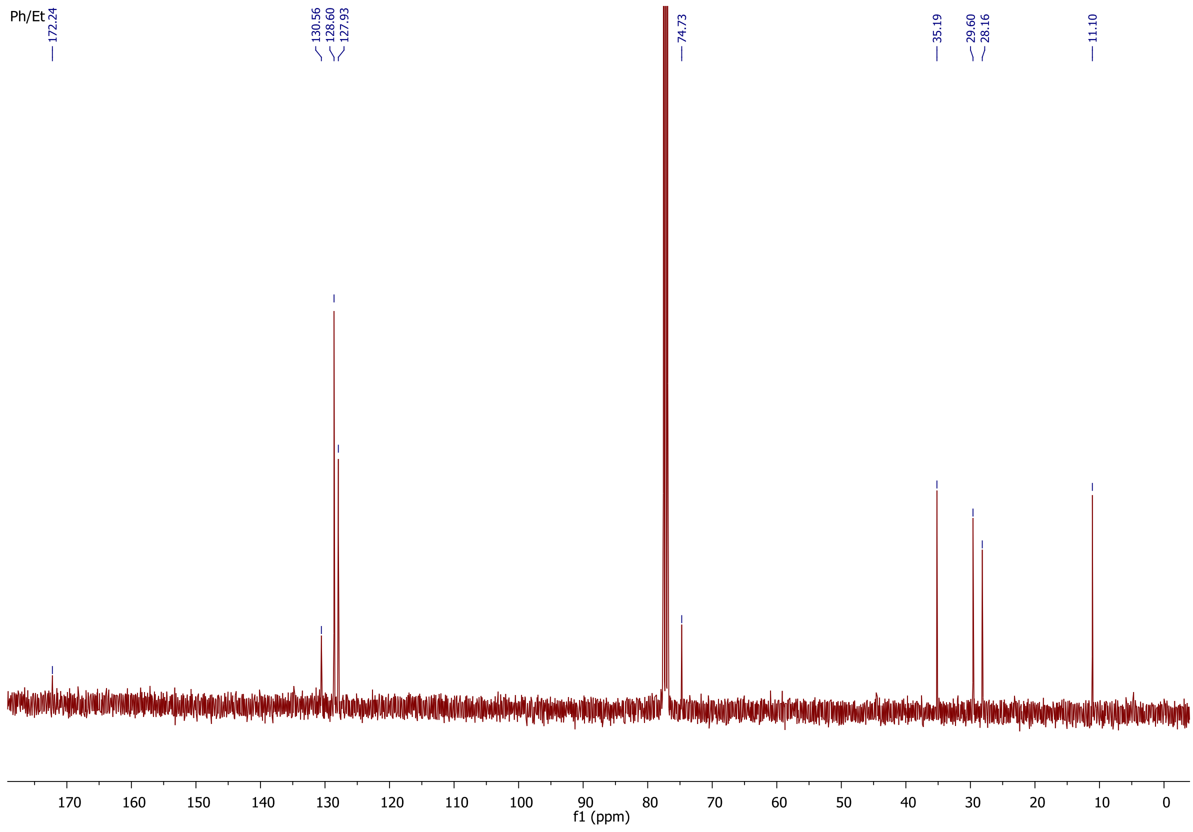
Ph/Et
172.24
130.56
128.60
127.93
74.73
35.19
29.60
28.16
11.10
170 160 150 140 130 120 110 100 90 80 70 60 50 40 30 20 10 0
f1 (ppm)

pTol/Et

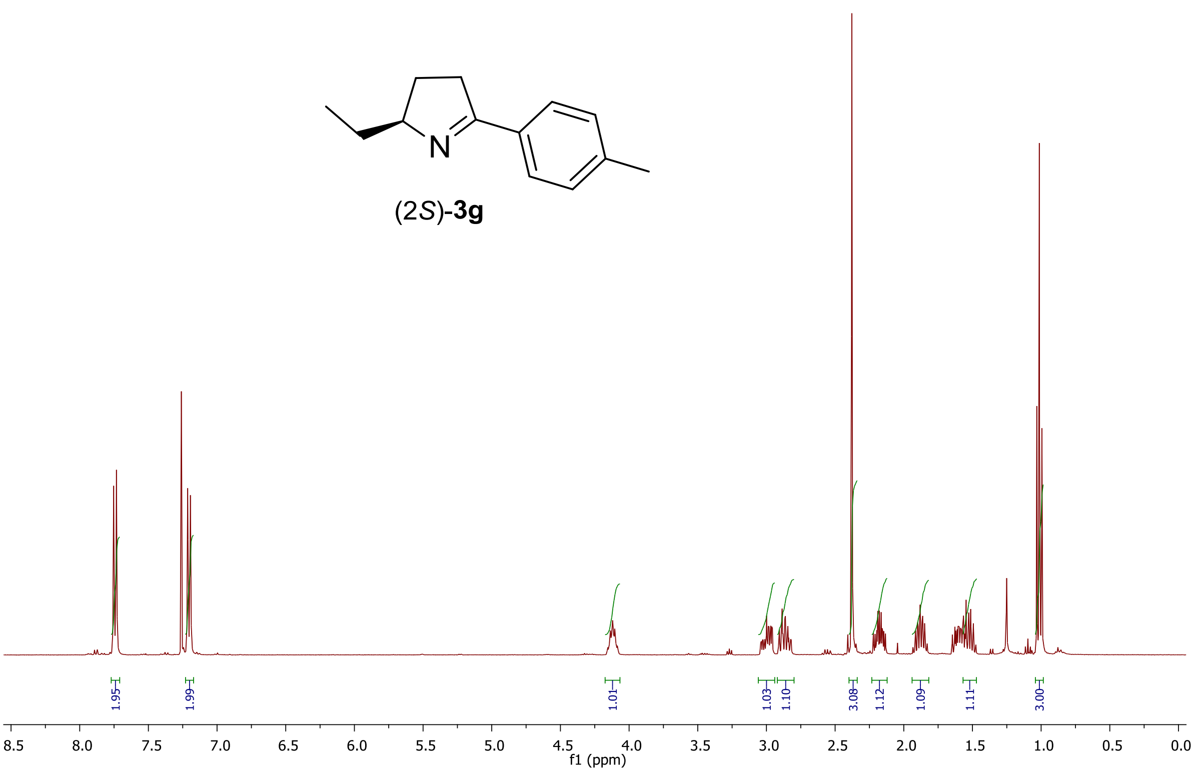

N
(2*S*)-**3g**
1.95
1.99
1.01
1.03
1.10
3.08
1.12
1.09
1.11
3.00
8.5 8.0 7.5 7.0 6.5 6.0 5.5 5.0 4.5 4.0 3.5 3.0 2.5 2.0 1.5 1.0 0.5 0.0
f1 (ppm)

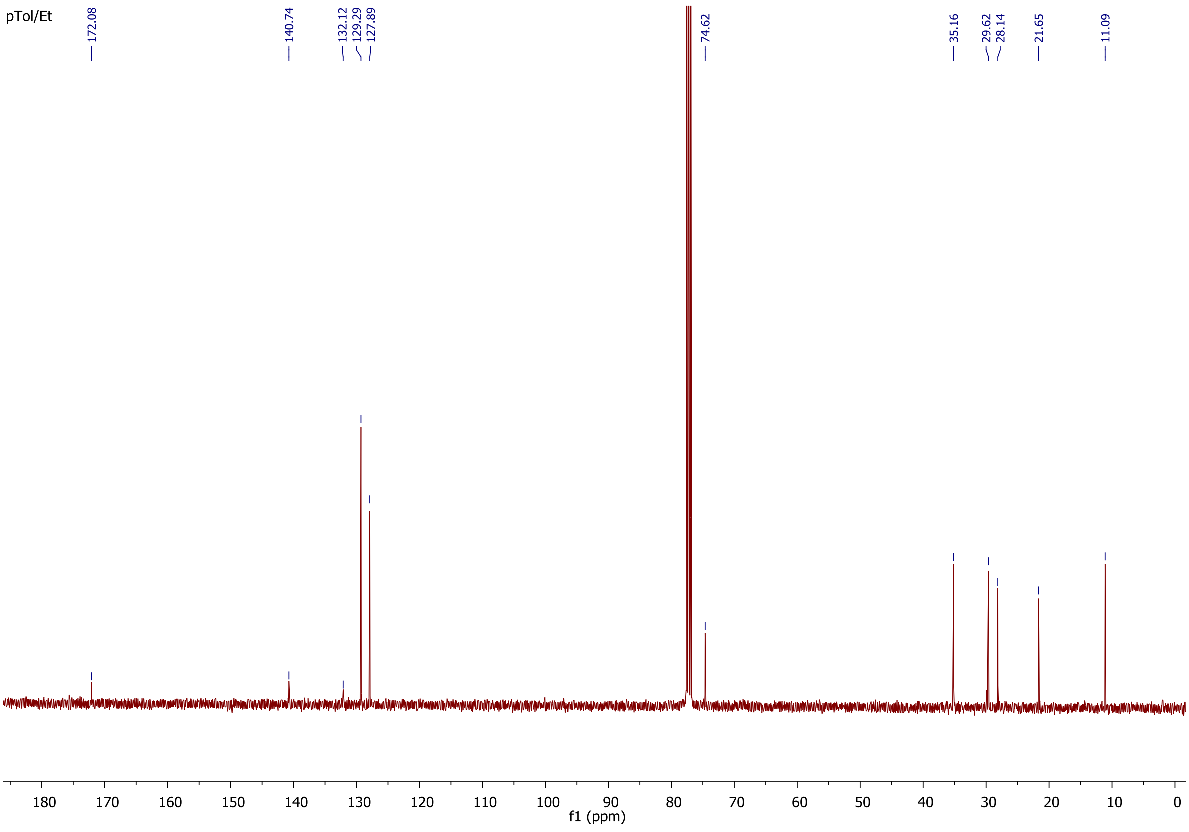

pTol/Et
172.08
140.74
132.12
129.29
127.89
74.62
35.16
29.62
28.14
21.65
11.09
180 170 160 150 140 130 120 110 100 90 80 70 60 50 40 30 20 10 0
f1 (ppm)

NMR Spectra of 4a-g Standards Following Treatment with Sodium Borohydride

S Ph/Me pyrrolodine

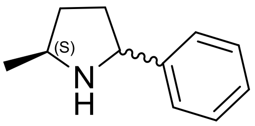

4a

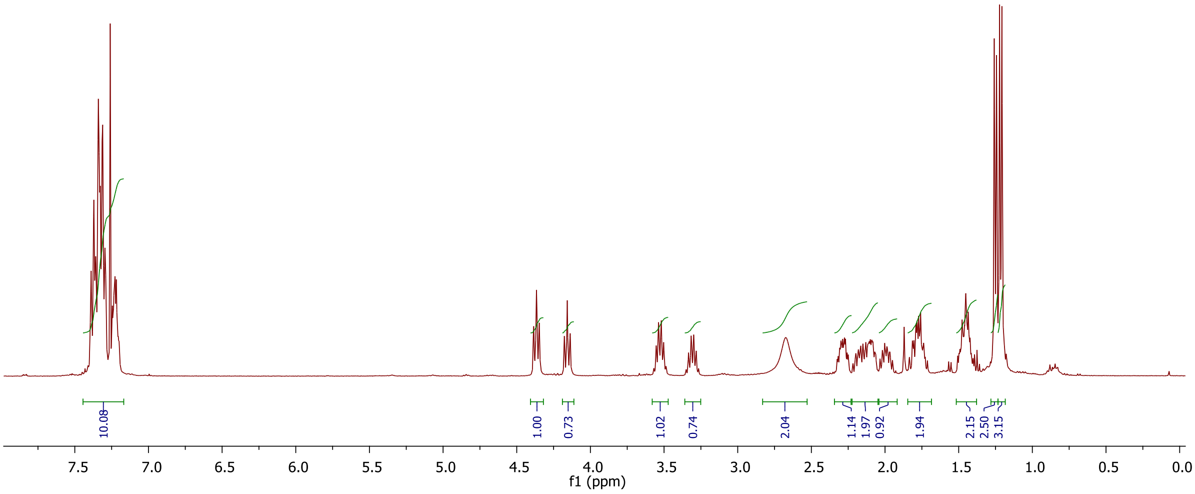

S Ph/Me pyrrolodine

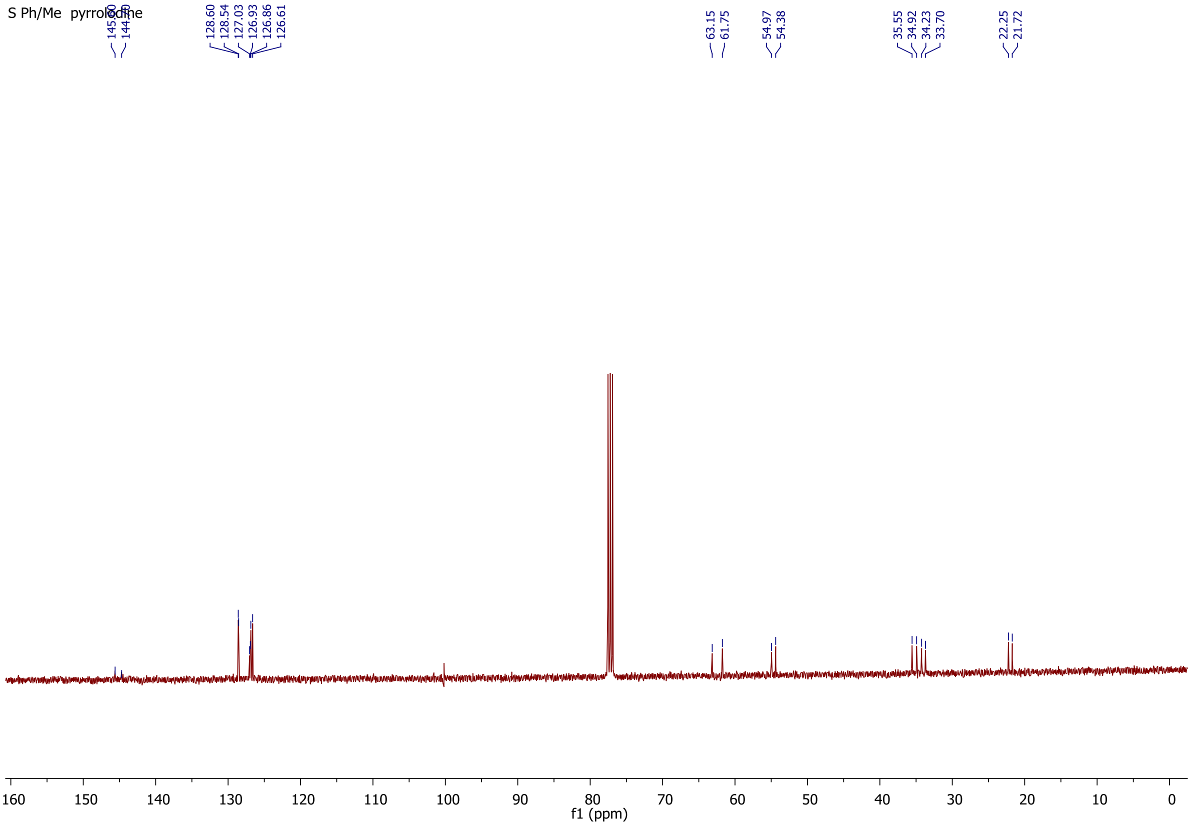

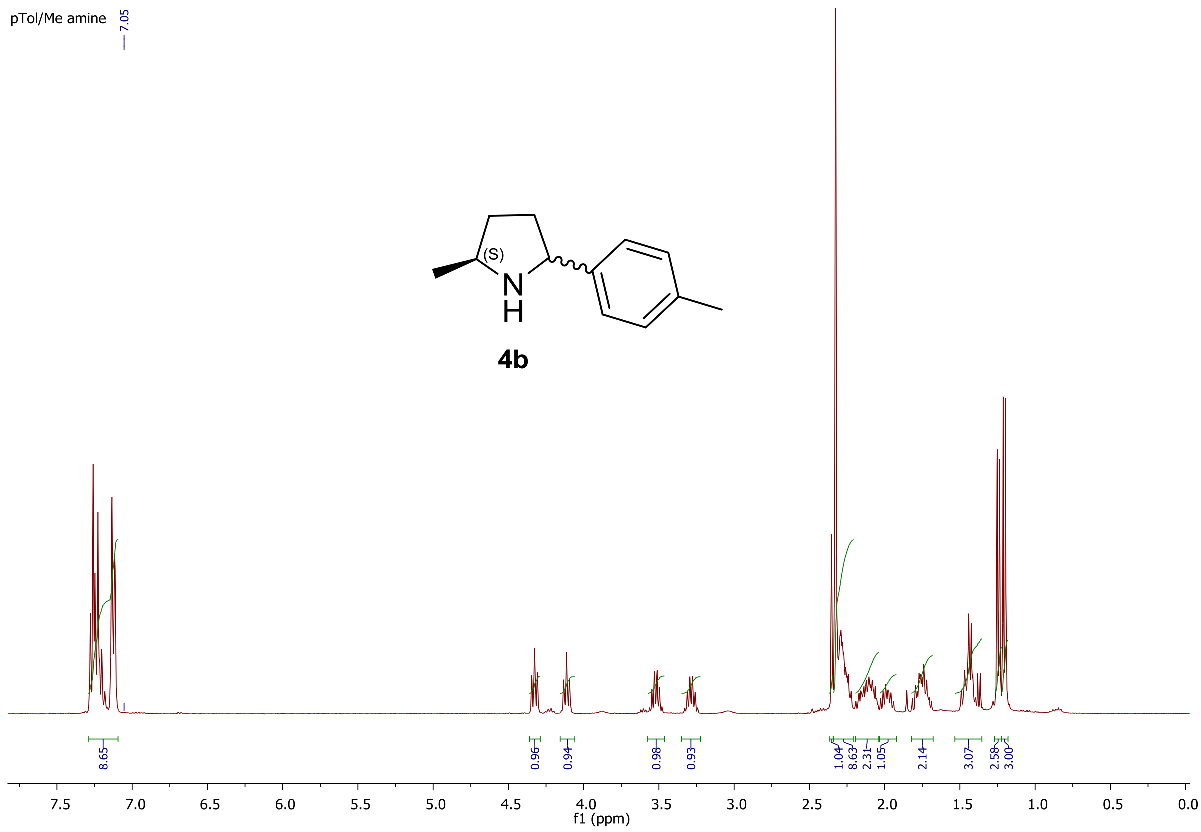
pTol/Me amine
7.05
(S)
N
H
4b
8.65
0.96
0.94
0.98
0.93
1.04
8.63
2.31
1.05
2.14
3.07
2.58
3.00
f1 (ppm)

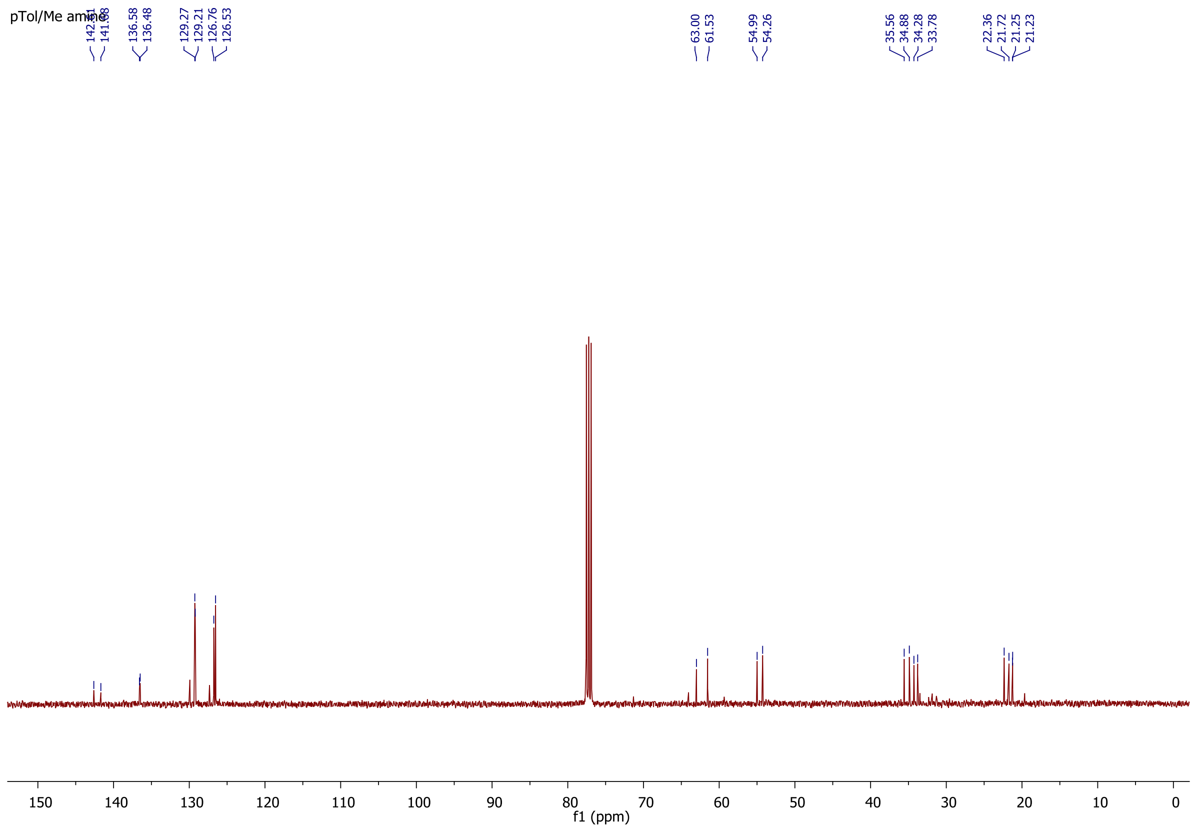
pTol/Me amine
136.58
136.48
129.27
129.21
126.76
126.53
63.00
61.53
54.99
54.26
35.56
34.88
34.28
33.78
22.36
21.72
21.25
21.23
f1 (ppm)

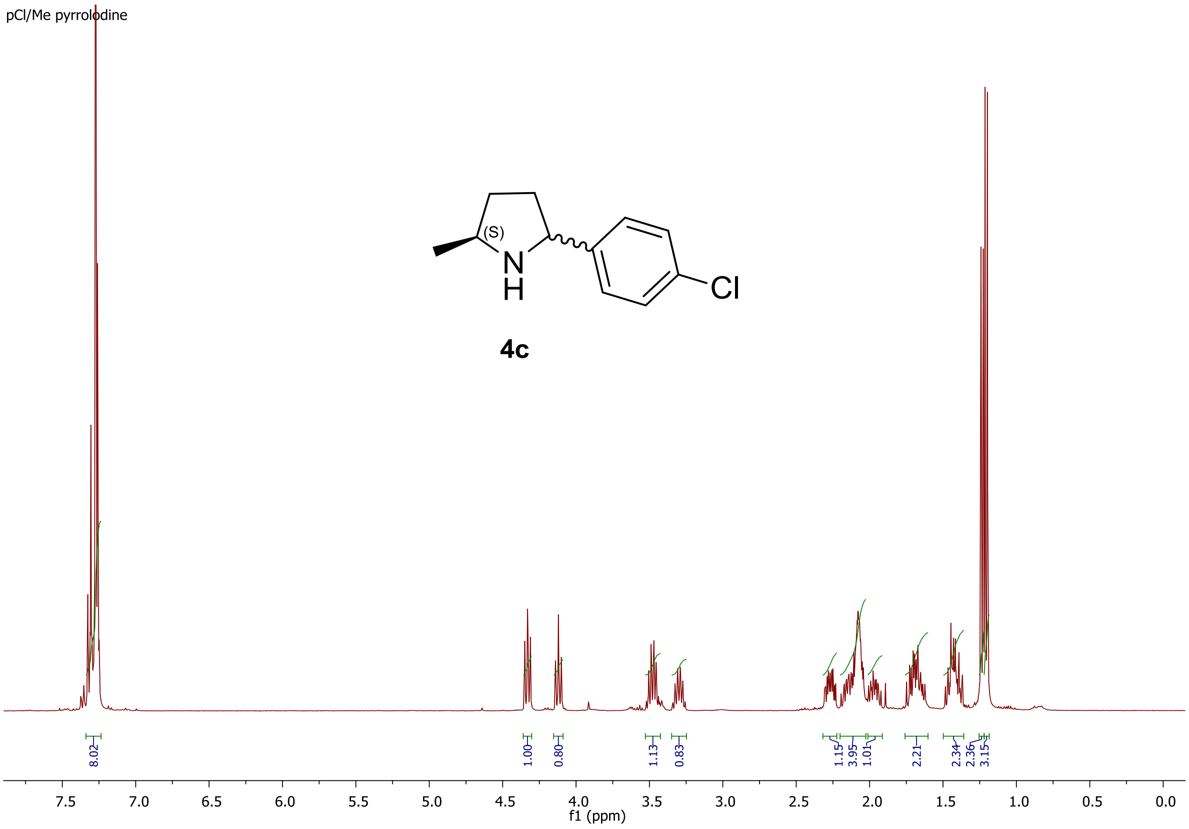
pCl/Me pyrrolodine
(S)
N
H
Cl
8.02
1.00
0.80
1.13
0.83
1.15
3.95
1.01
2.21
2.34
2.36
3.15
7.5 7.0 6.5 6.0 5.5 5.0 4.5 4.0 3.5 3.0 2.5 2.0 1.5 1.0 0.5 0.0
f1 (ppm)

4c

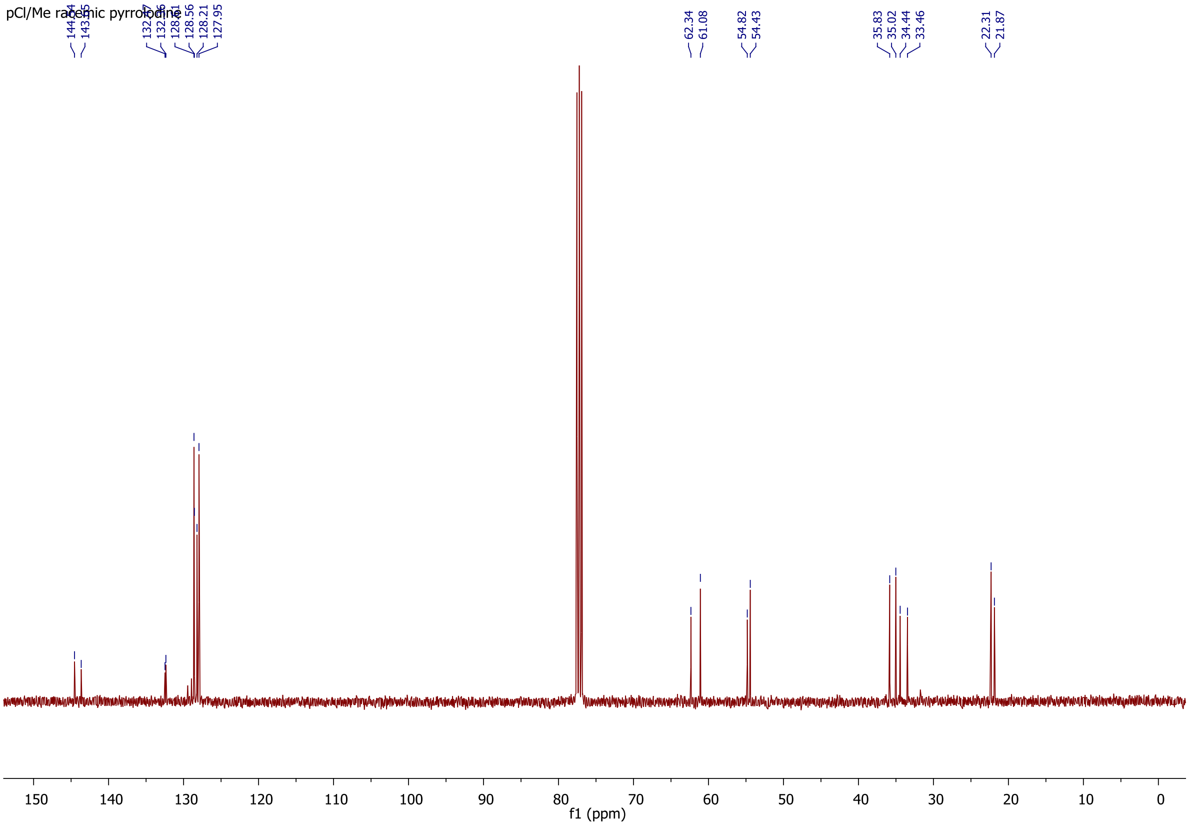
pCl/Me racemic pyrrolodine
128.56
128.21
127.95
62.34
61.08
54.82
54.43
35.83
35.02
34.44
33.46
22.31
21.87
150 140 130 120 110 100 90 80 70 60 50 40 30 20 10 0
f1 (ppm)

pF/Me

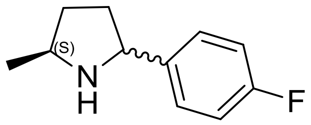

4d

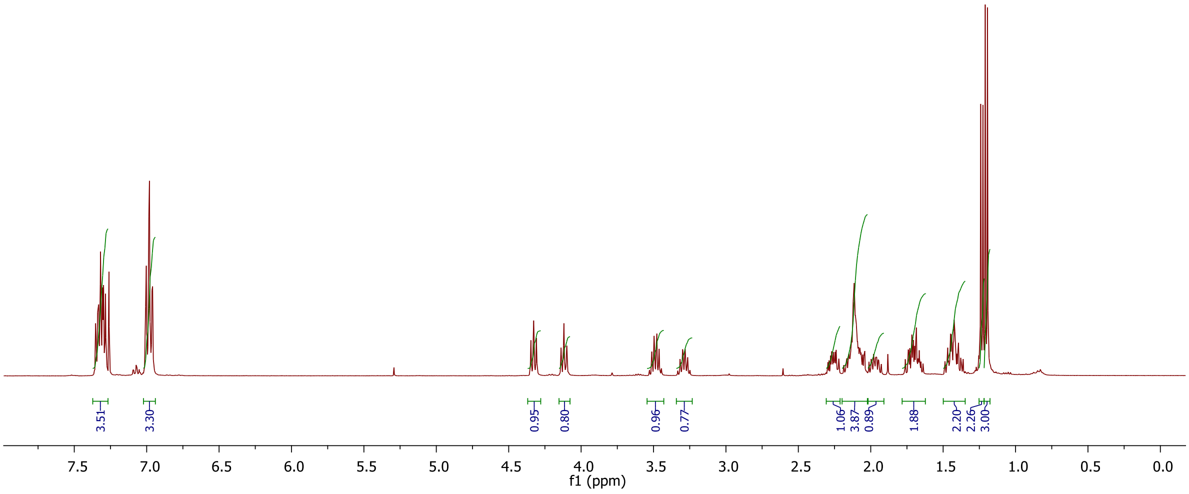

pF/Me pyrrolidine

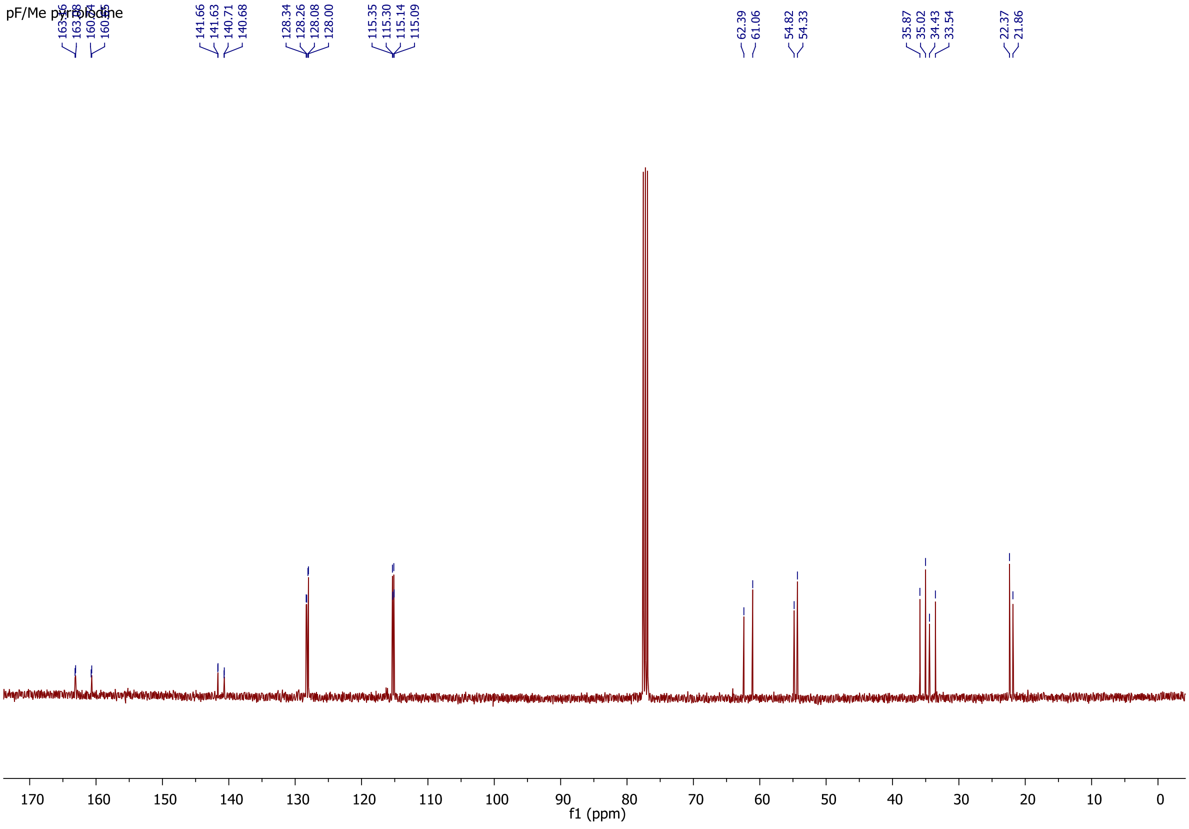

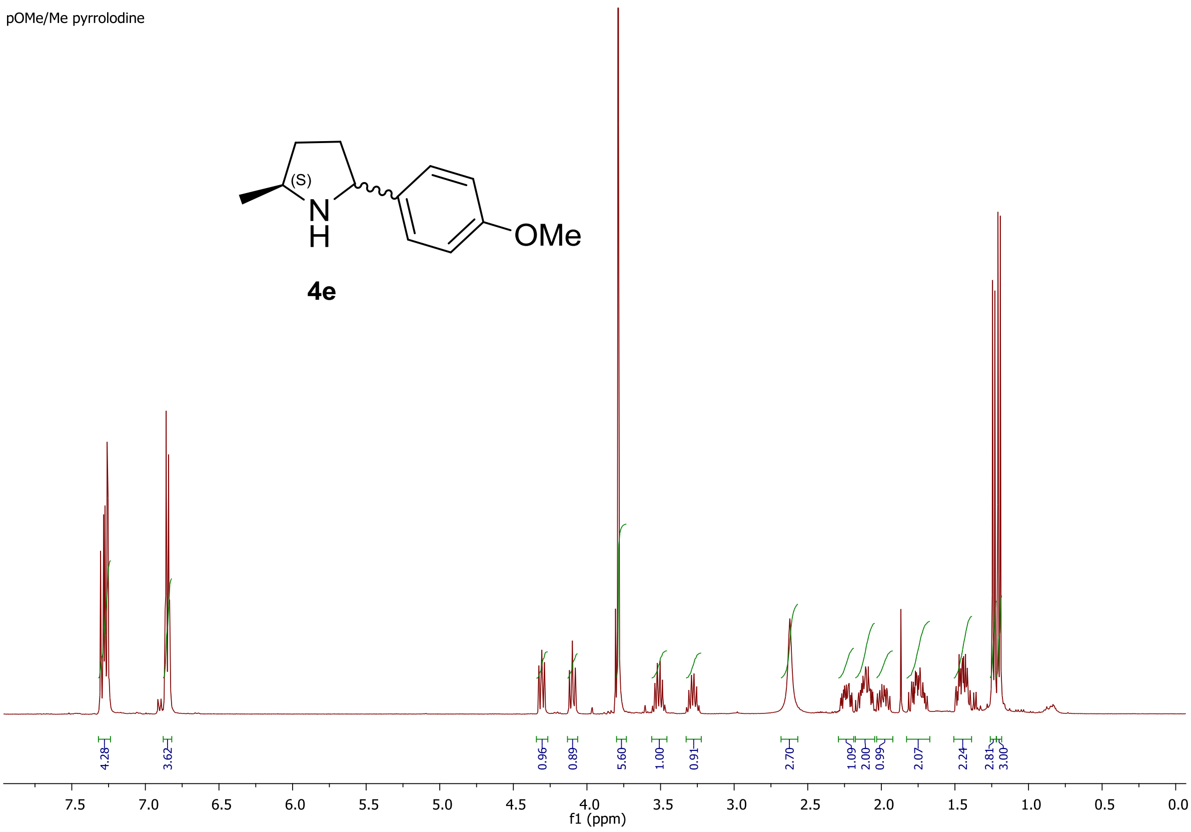
pOMe/Me pyrrolodine
(S)
N
H
OMe
4e
4.28
3.62
0.96
0.89
5.60
1.00
0.91
2.70
1.09
2.00
0.99
2.07
2.24
2.81
3.00
7.5 7.0 6.5 6.0 5.5 5.0 4.5 4.0 3.5 3.0 2.5 2.0 1.5 1.0 0.5 0.0
f1 (ppm)

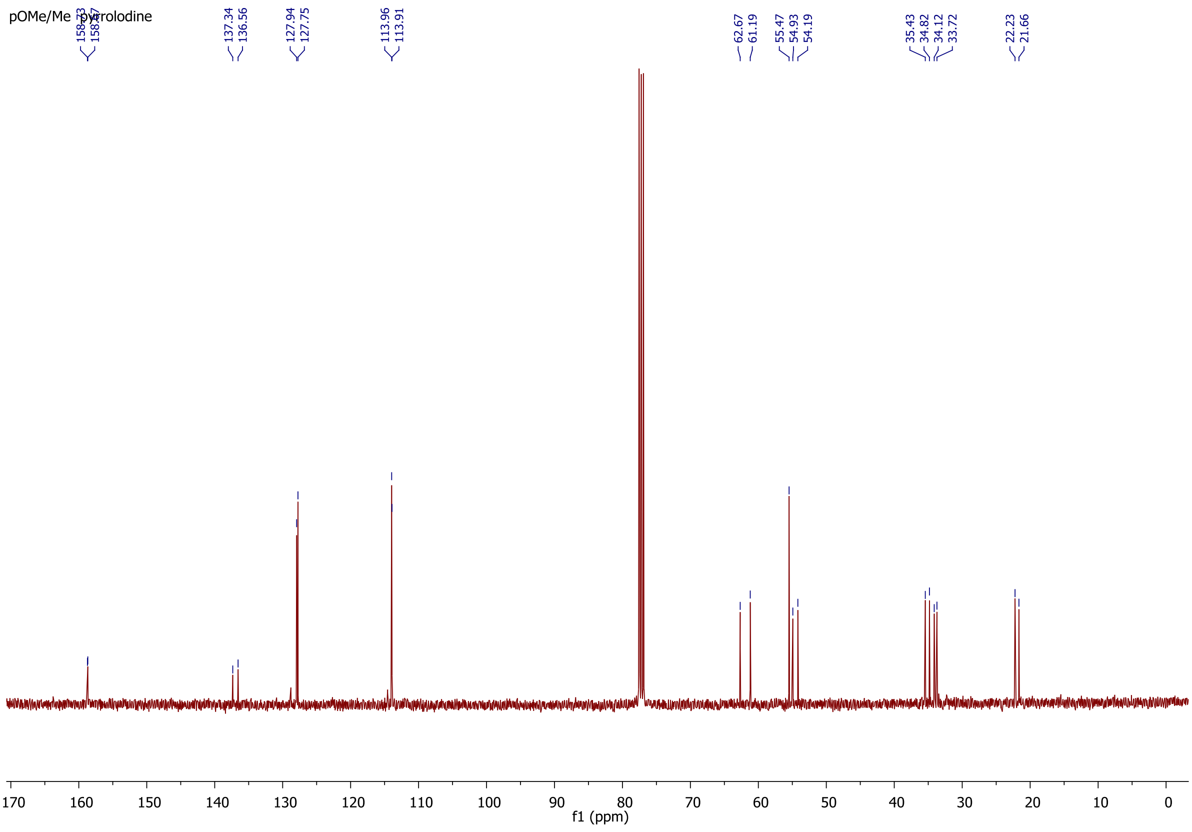
pOMe/Me pyrrolodine
137.34
136.56
127.94
127.75
113.96
113.91
62.67
61.19
55.47
54.93
54.19
35.43
34.82
34.12
33.72
22.23
21.66
170 160 150 140 130 120 110 100 90 80 70 60 50 40 30 20 10 0
f1 (ppm)

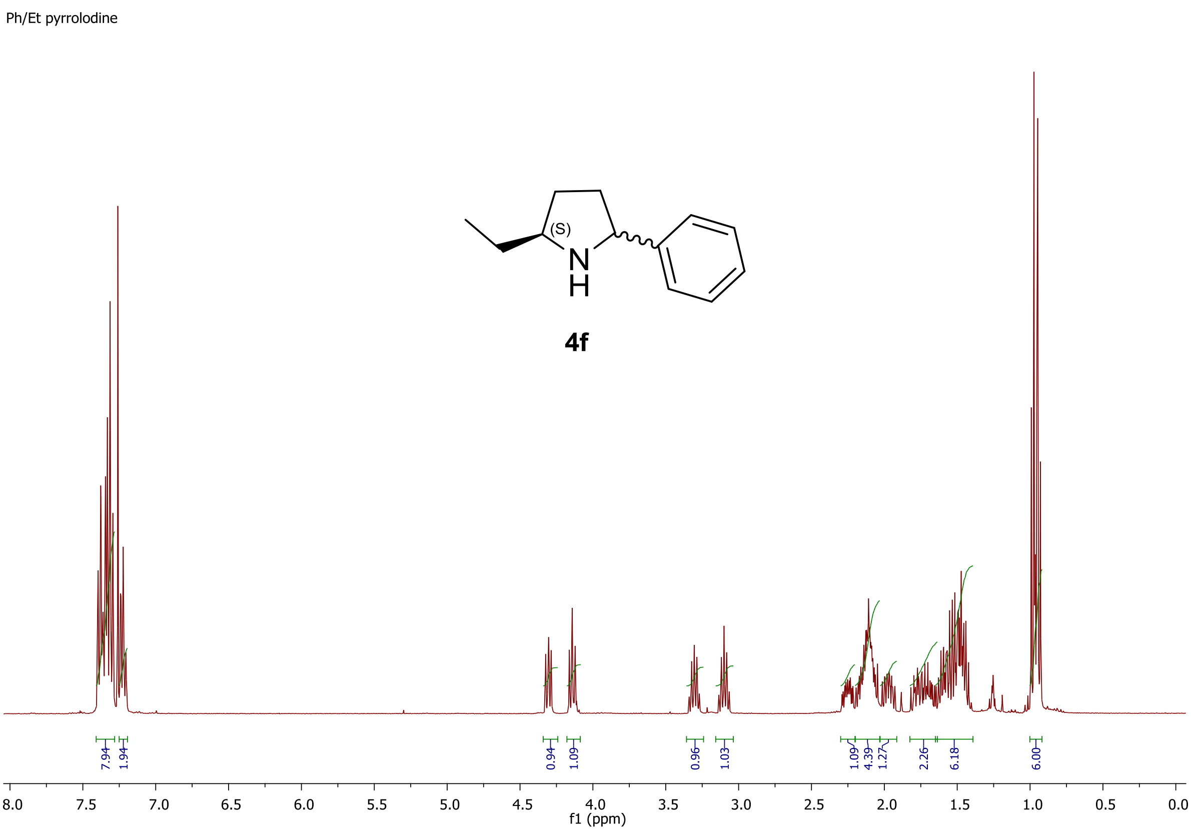
Ph/Et pyrrolodine
(S)
N
H
4f
7.94
1.94
0.94
1.09
0.96
1.03
1.09
4.39
1.27
2.26
6.18
6.00
8.0
7.5
7.0
6.5
6.0
5.5
5.0
4.5
4.0
3.5
3.0
2.5
2.0
1.5
1.0
0.5
0.0
f1 (ppm)

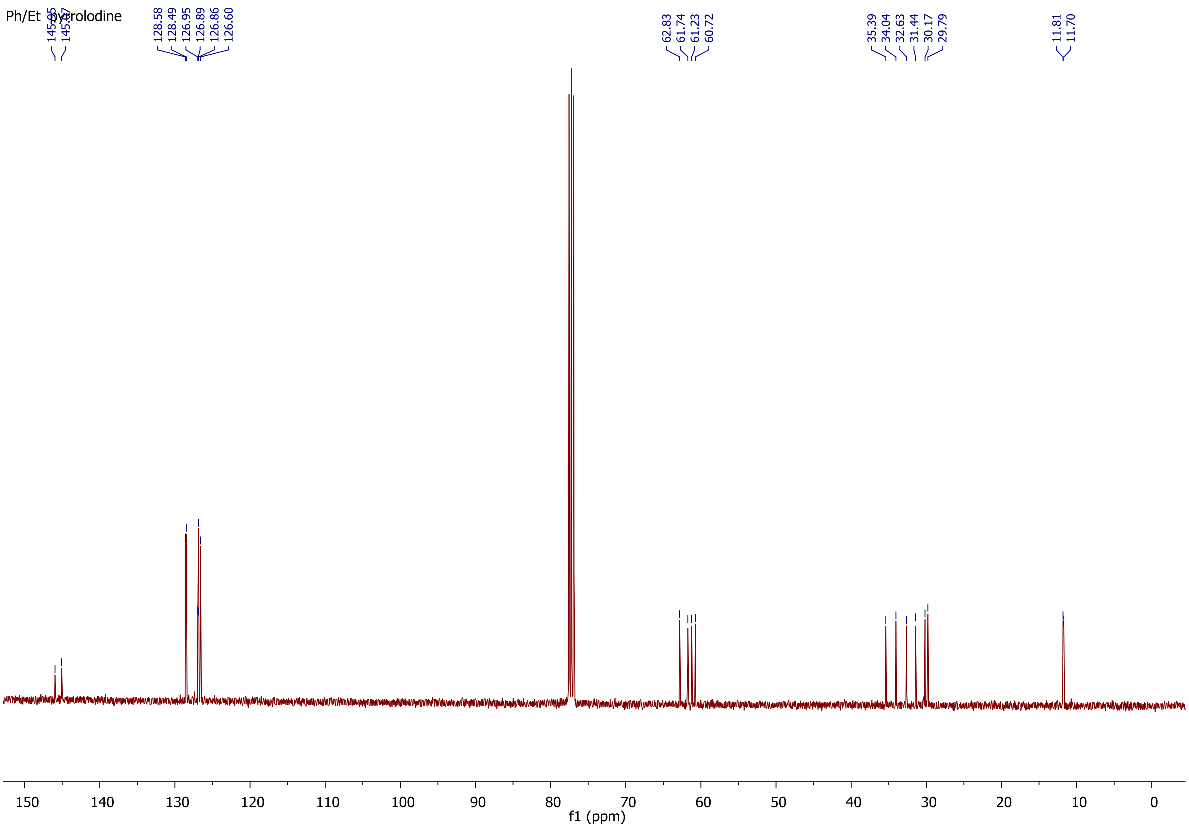
Ph/Et pyrrolodine
128.58
128.49
126.95
126.89
126.86
126.60
62.83
61.74
61.23
60.72
35.39
34.04
32.63
31.44
30.17
29.79
11.81
11.70
150
140
130
120
110
100
90
80
70
60
50
40
30
20
10
0
f1 (ppm)

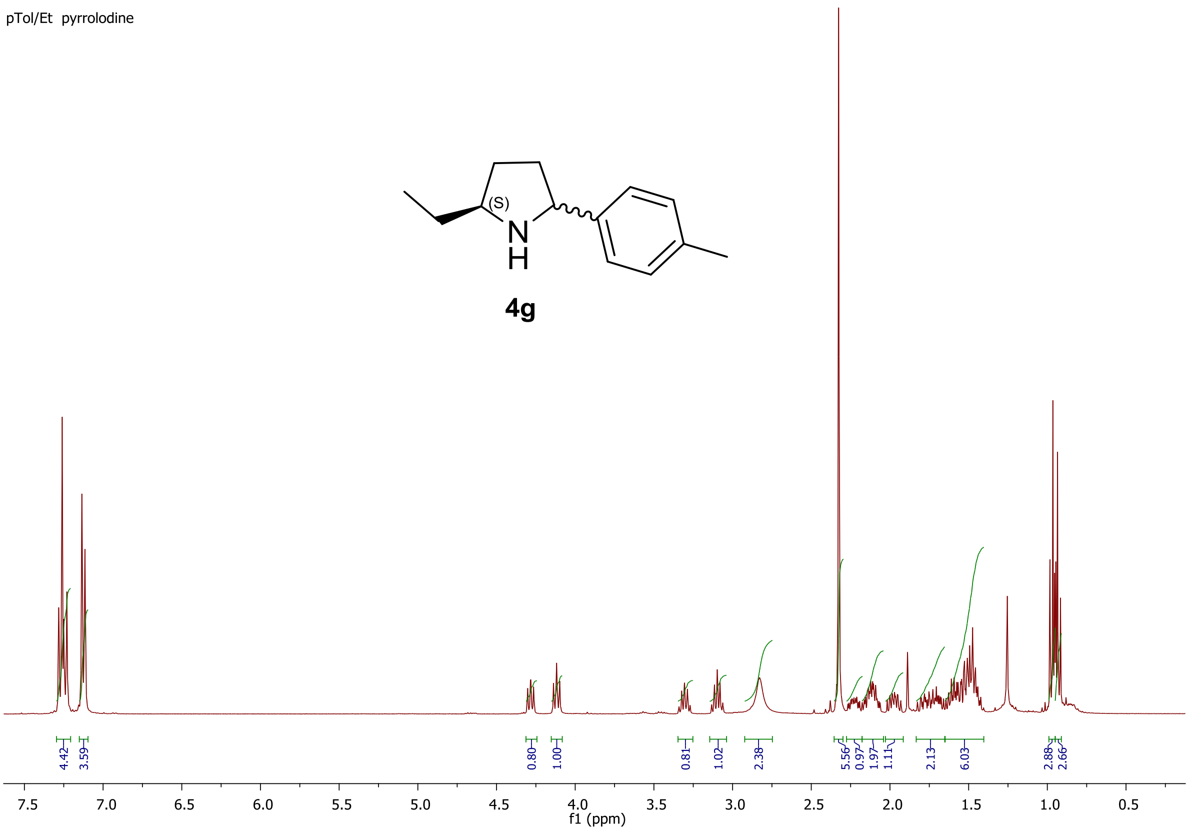
pTol/Et pyrrolodine
(S)
N
H
4g
4.42
3.59
0.80
1.00
0.81
1.02
2.38
5.56
0.97
1.97
1.11
2.13
6.03
2.88
2.66
7.5 7.0 6.5 6.0 5.5 5.0 4.5 4.0 3.5 3.0 2.5 2.0 1.5 1.0 0.5
f1 (ppm)

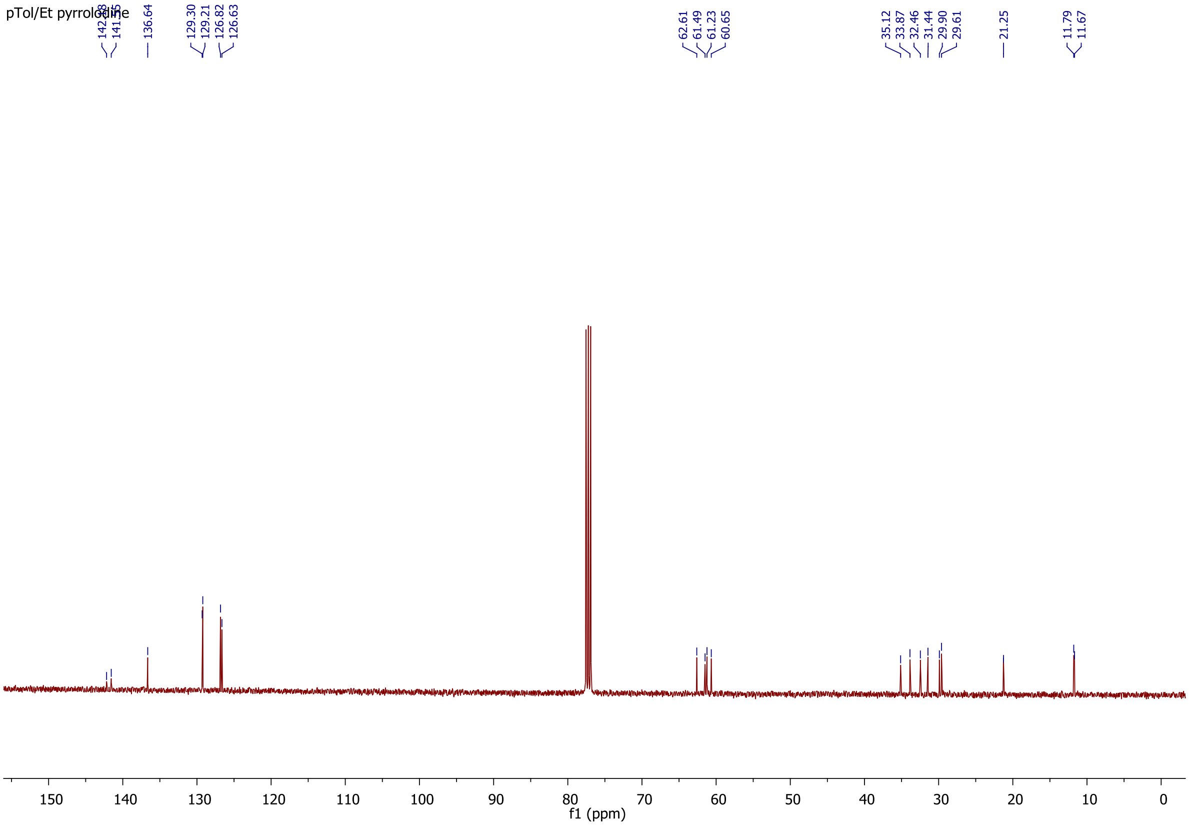
pTol/Et pyrrolodine
141.85
136.64
129.30
129.21
126.82
126.63
62.61
61.49
61.23
60.65
35.12
33.87
32.46
31.44
29.90
29.61
21.25
11.79
11.67
150 140 130 120 110 100 90 80 70 60 50 40 30 20 10 0
f1 (ppm)

NMR Spectra of ATA113/MAO-N D9 One-pot Cascade Product (2*S*,5*R*)-4a

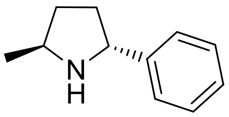

(2*S*,5*R*)-**4a**

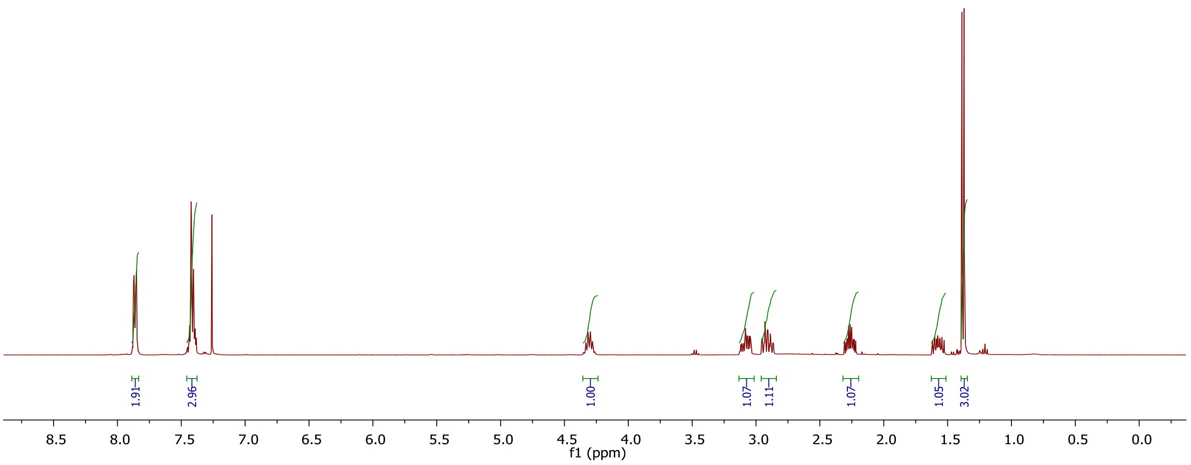

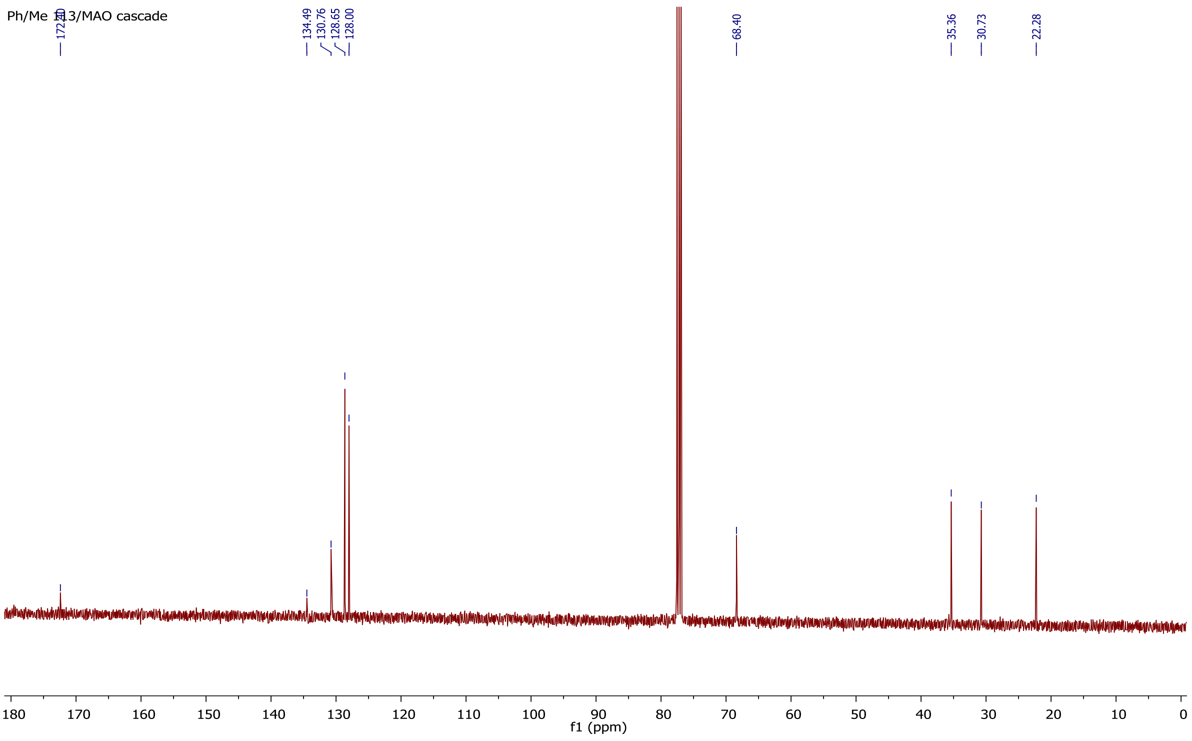

GC/FID Chromatograms

GC/FID Chromatograms of (*S*)-**3a-g** following ATA113 preparative-scale reactions with diketones **1a-g**

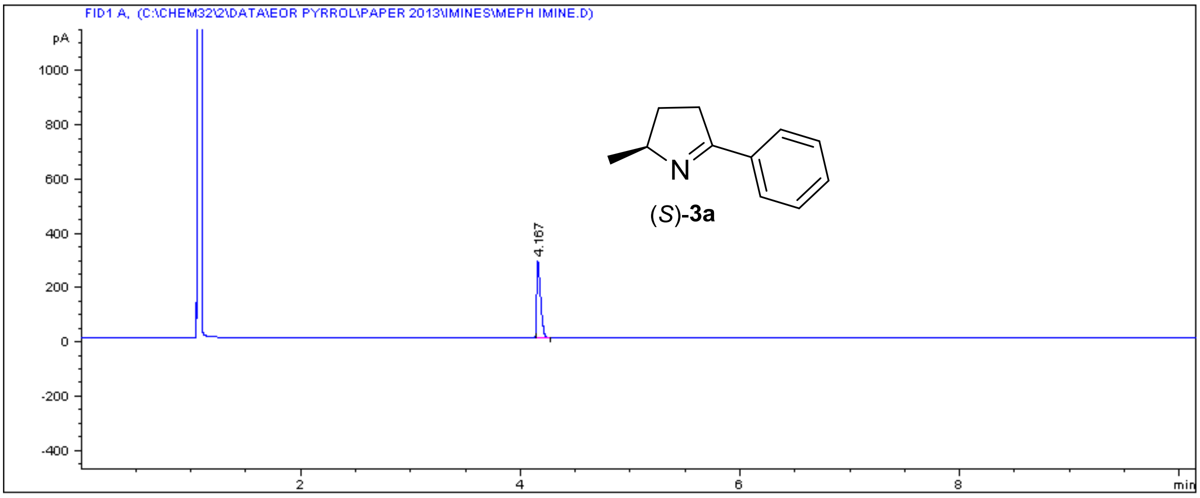

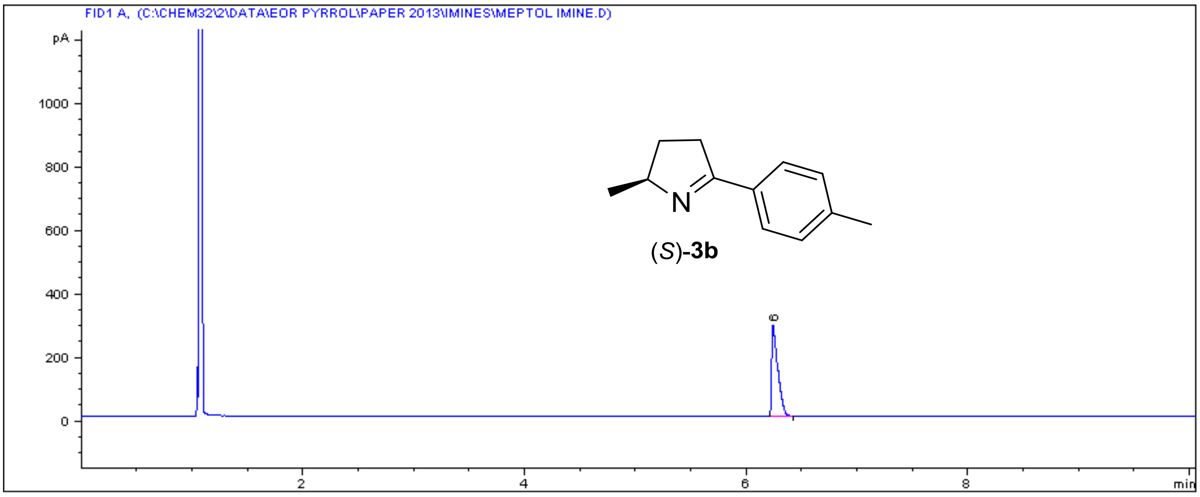

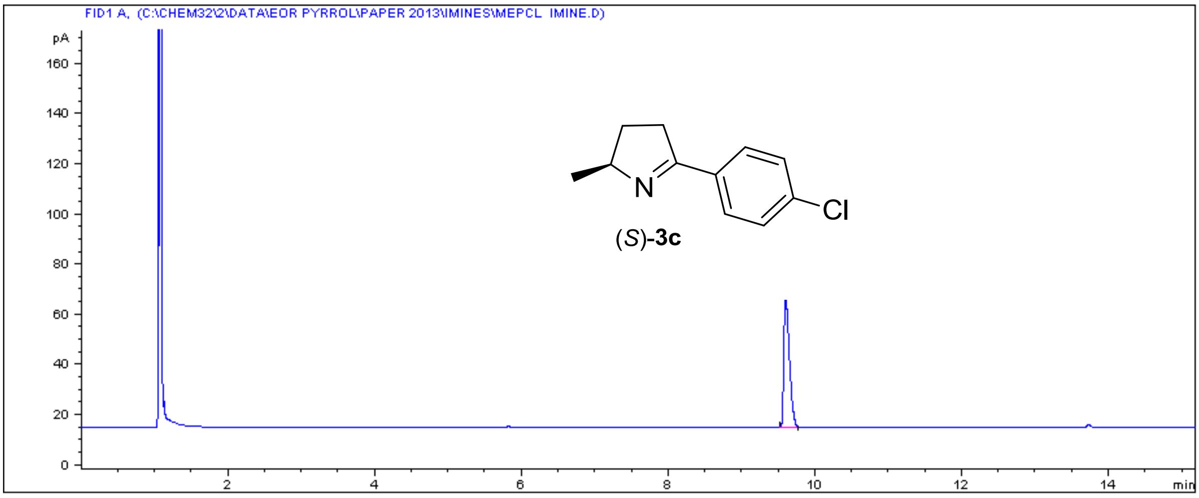

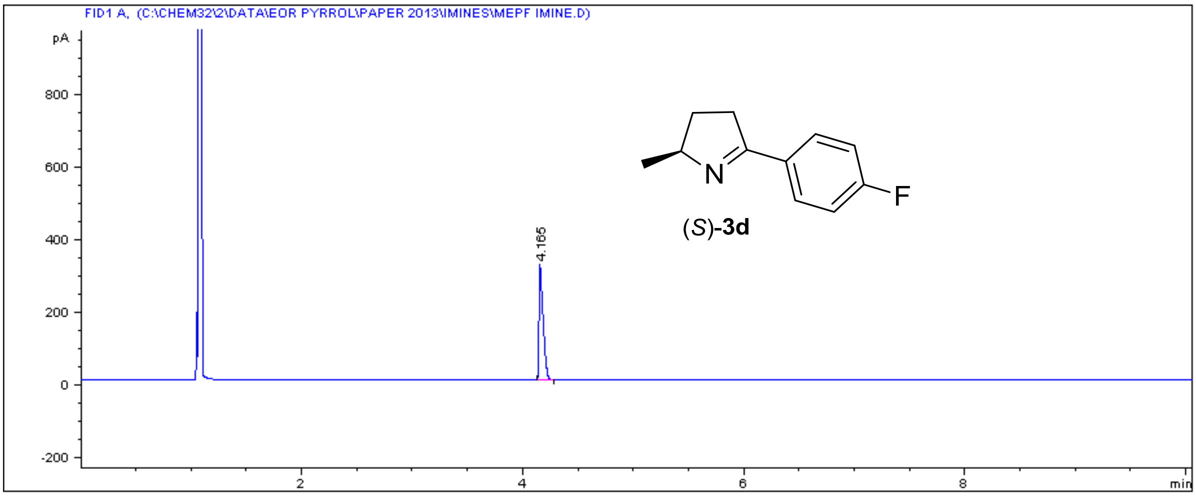
FID1 A, (C:\CHEM32\2\DATA\EOR PYRROL\PAPER 2013\IMINES\MEPF IMINE.D)
pA
4.165
F
N
(S)-3d
min

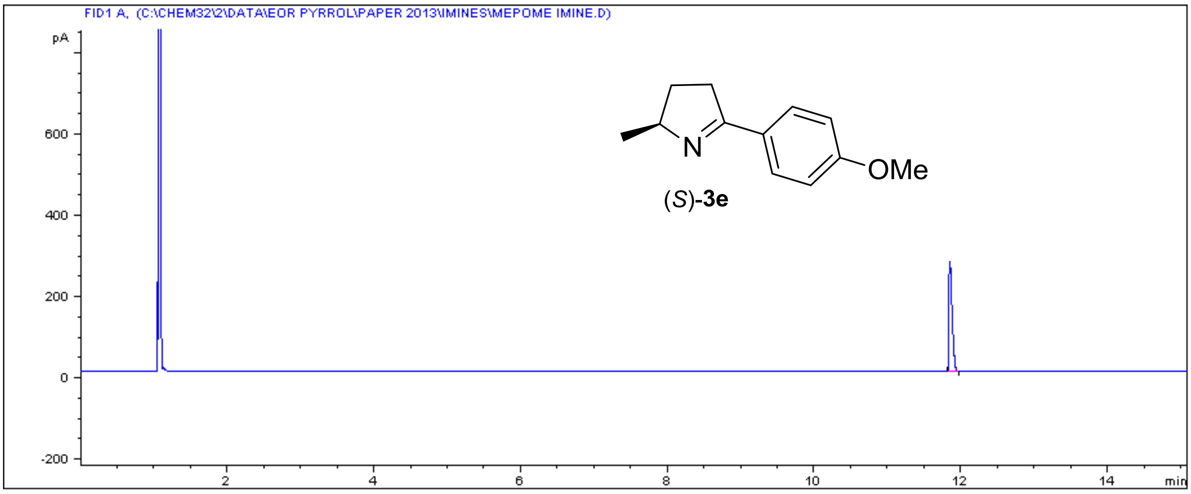
FID1 A, (C:\CHEM32\2\DATA\EOR PYRROL\PAPER 2013\IMINES\MEPOME IMINE.D)
pA
N
OMe
(S)-3e
min

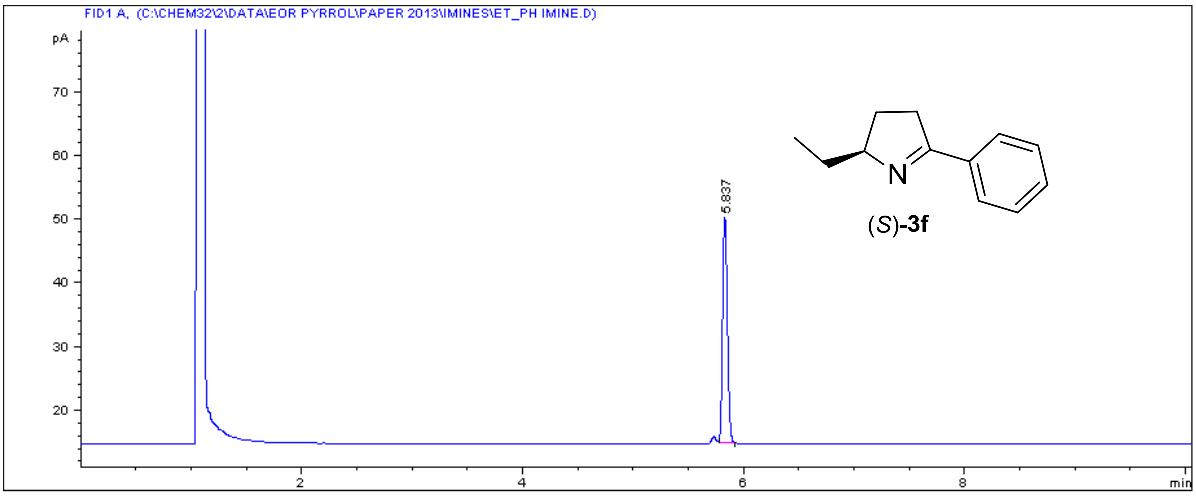
FID1 A, (C:\CHEM32\2\DATA\EOR PYRROL\PAPER 2013\IMINES\ET_PH IMINE.D)
pA
5.837
N
(S)-3f
min

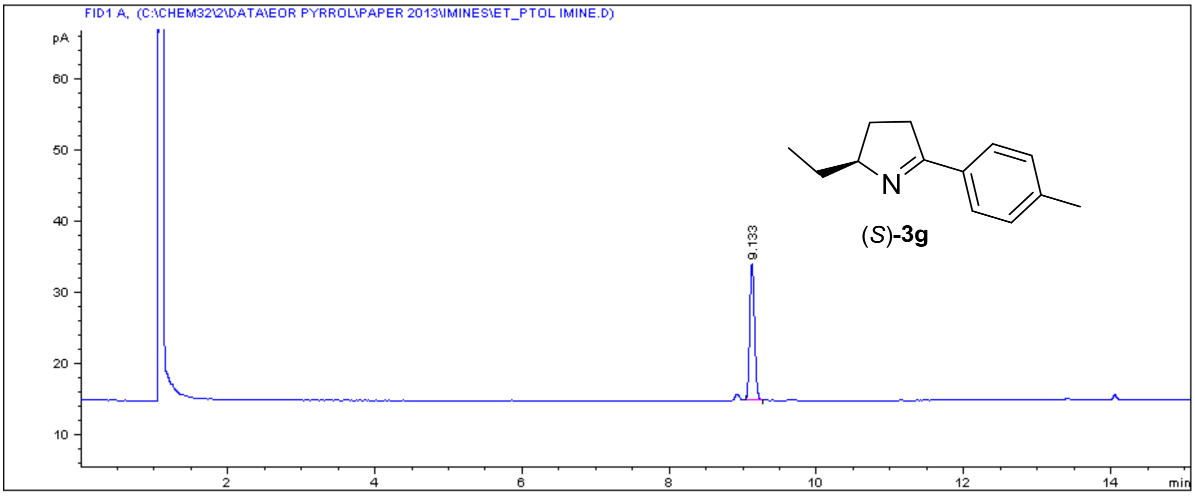

Determination of *Enantiomeric Excess* and Assignment of Absolute Configuration

The absolute configuration of isolated ATA113 products (*S*)-**3a**, (*S*)-**3b**, (*S*)-**3f** and ATA117 product (*R*)-**3a** were determined by comparing the measured optical rotation values to literature values.[3] The absolute configuration of (*S*)-**3c-e** and **3g** were assumed based on this trend. Chiral GC/FID of ATA113, ATA117 and *pf*-ATA products **3a**, **3d** and **3e** were compared to identify the (*S*)- and (*R*)-enantiomers and calculate the *ee*. In the absence of the (*R*)-enantiomers, **3b-c** and **3f** were assigned by comparison.

Table 1.

Entry	Product	ω-TA	*ee* [%]	Assignment of *ee*
1	**3a**	ATA113	> 99 (*S*)	Optical rotation[3]/Chiral GC
2	**3a**	ATA117	> 99 (*R*)	Optical rotation[3]/Chiral GC
3	**3a**	*P. ferrooxidans*	75 (*S*)	Chiral GC
4	**3a**	*C. violaceum*	60 (*S*)	Chiral GC
5	**3b**	ATA113	> 99 (*S*)	Optical rotation[3]/Chiral GC
6	**3b**	*P. ferrooxidans*	> 78 (*S*)	Chiral GC
7	**3c**	ATA113	> 99 (*S*)	Chiral GC
8	**3c**	*P. ferrooxidans*	68 (*S*)	Chiral GC
9	**3d**	ATA113	> 99 (*S*)	Chiral GC
10	**3d**	ATA117	> 99 (*R*)	Chiral GC
11	**3d**	*P. ferrooxidans*	76 (*S*)	Chiral GC
12	**3d**	*C. violaceum*	73 (*S*)	Chiral GC
13	**3e**	ATA113	> 99 (*S*)	Chiral GC
14	**3e**	ATA117	> 99 (*R*)	Chiral GC
15	**3e**	*P. ferrooxidans*	78 (*S*)	Chiral GC
16	**3e**	*C. violaceum*	80 (*S*)	Chiral GC
17	**3f**	ATA113	96 (*S*)	Optical rotation[3]/Chiral GC
18	**3f**	*P. ferrooxidans*	76 (*R*)	Chiral GC
19	**3g**	ATA113	94 (*S*)	Chiral GC
20	**3g**	*P. ferrooxidans*	46 (*R*)	Chiral GC

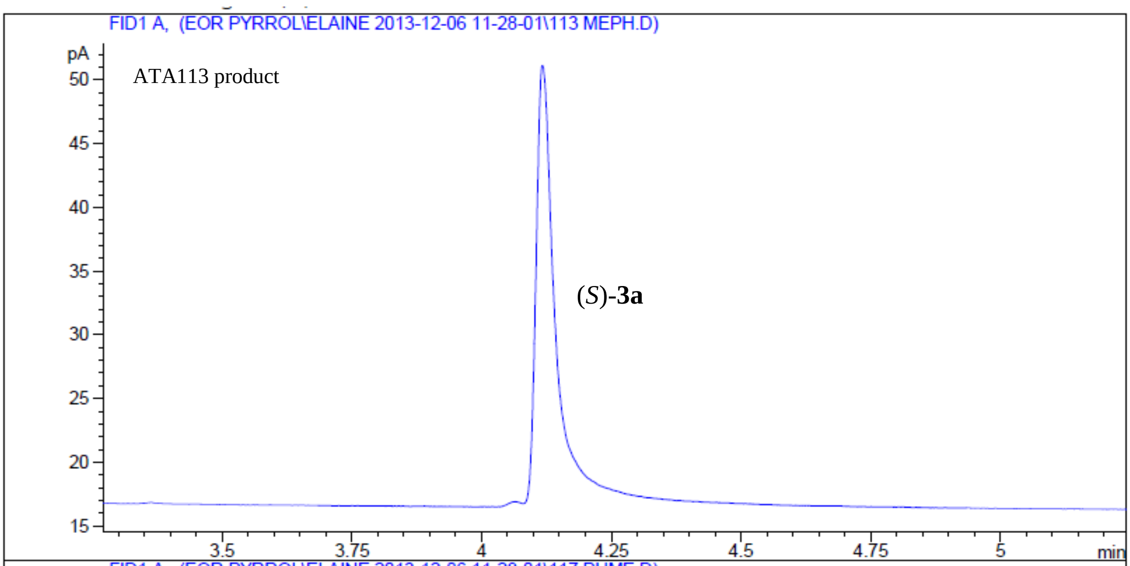
FID1 A, (EOR PYRROL\ELAINE 2013-12-06 11-28-01\113 MEPH.D)
pA
50
45
40
35
30
25
20
15
ATA113 product
(S)-3a
3.5
3.75
4
4.25
4.5
4.75
5
min

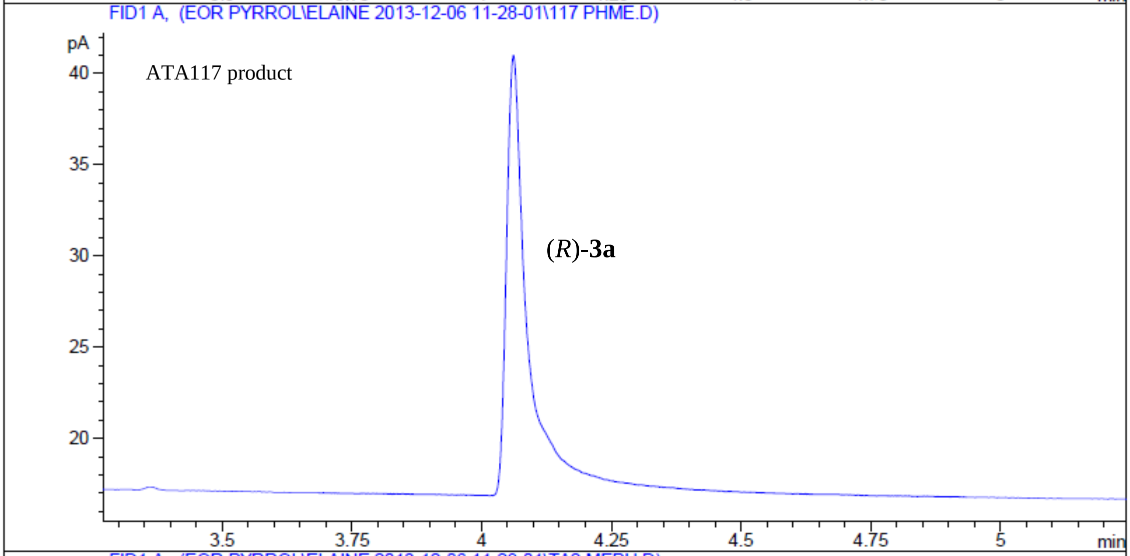
FID1 A, (EOR PYRROL\ELAINE 2013-12-06 11-28-01\117 PHME.D)
pA
40
35
30
25
20
ATA117 product
(R)-3a
3.5
3.75
4
4.25
4.5
4.75
5
min

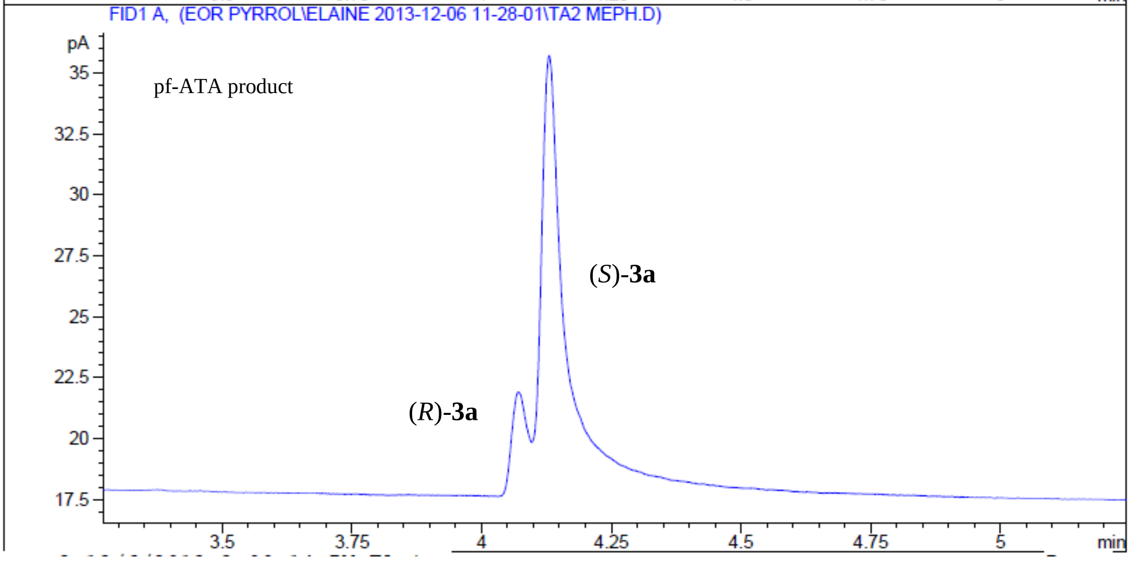
FID1 A, (EOR PYRROL\ELAINE 2013-12-06 11-28-01\TA2 MEPH.D)
pA
35
32.5
30
27.5
25
22.5
20
17.5
pf-ATA product
(S)-3a
(R)-3a
3.5
3.75
4
4.25
4.5
4.75
5
min

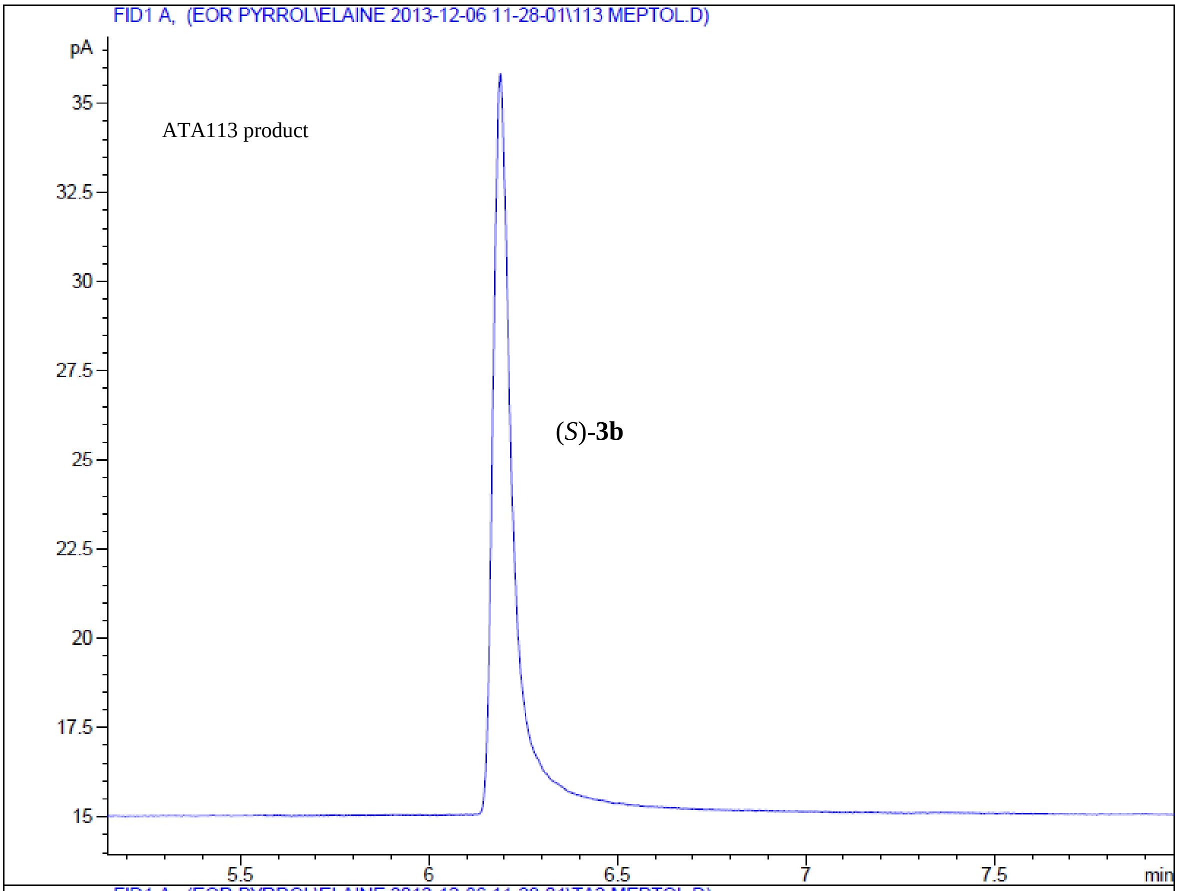
FID1 A, (EOR PYRROL\ELAINE 2013-12-06 11-28-01\113 MEPTOL.D)
pA
ATA113 product
(*S*)-**3b**
min

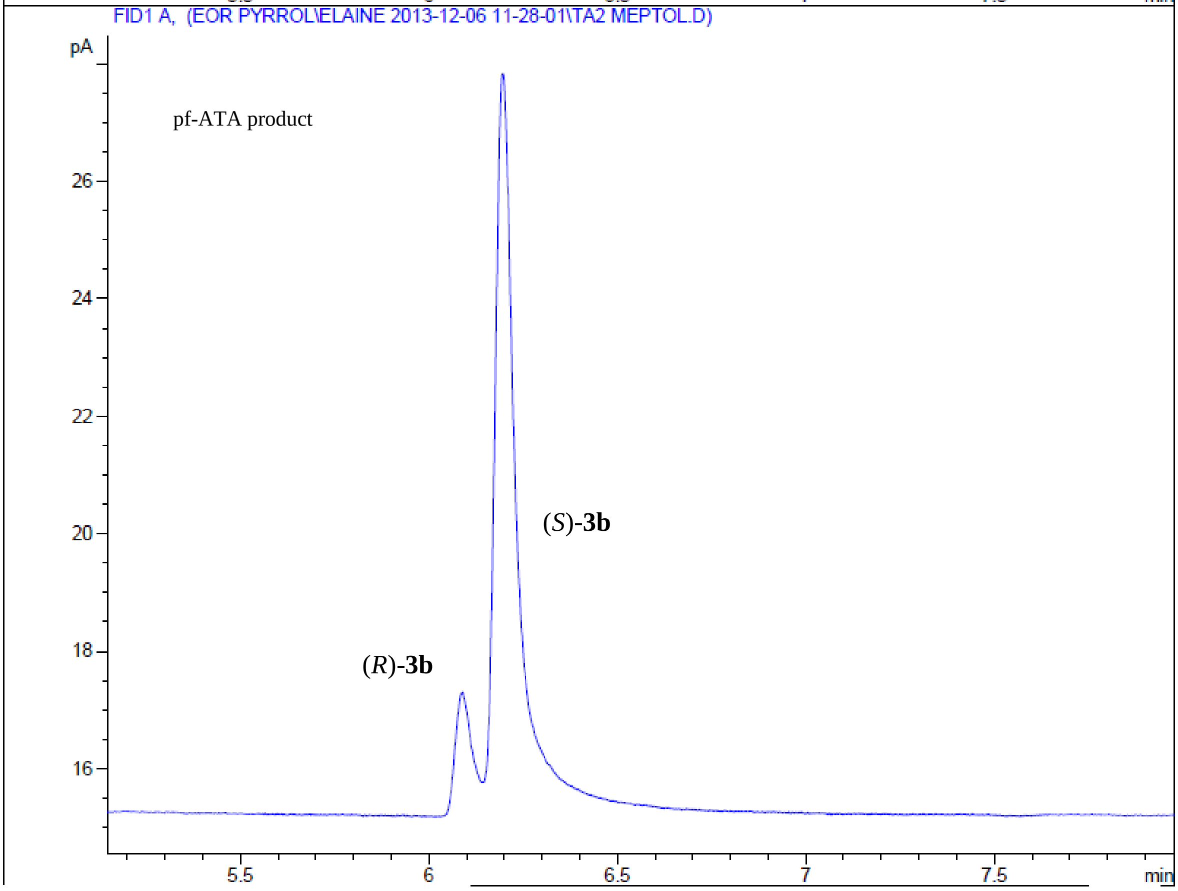
FID1 A, (EOR PYRROL\ELAINE 2013-12-06 11-28-01\TA2 MEPTOL.D)
pA
pf-ATA product
(*S*)-**3b**
(*R*)-**3b**
min

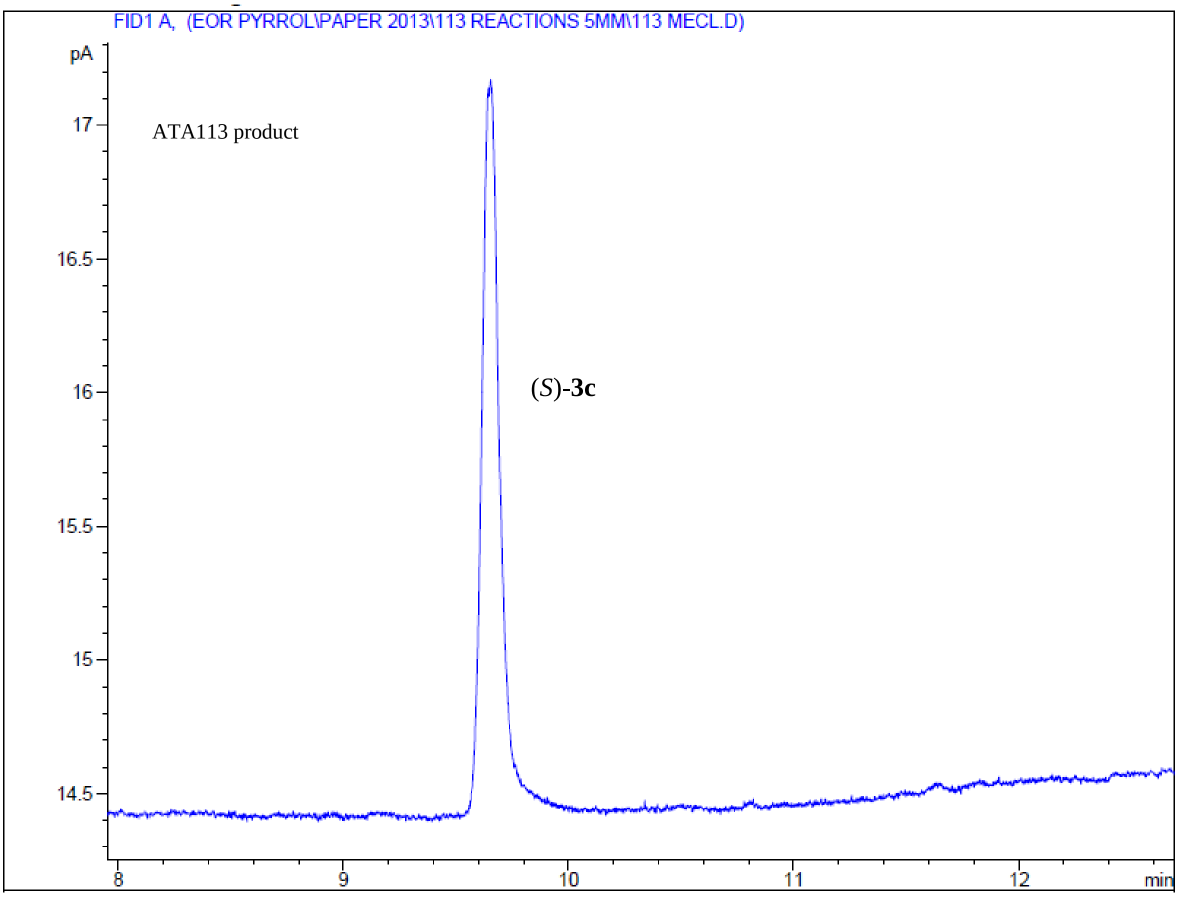
FID1 A, (EOR PYRROL\PAPER 2013\113 REACTIONS 5MM\113 MECL.D)
pA
ATA113 product
(S)-3c
min

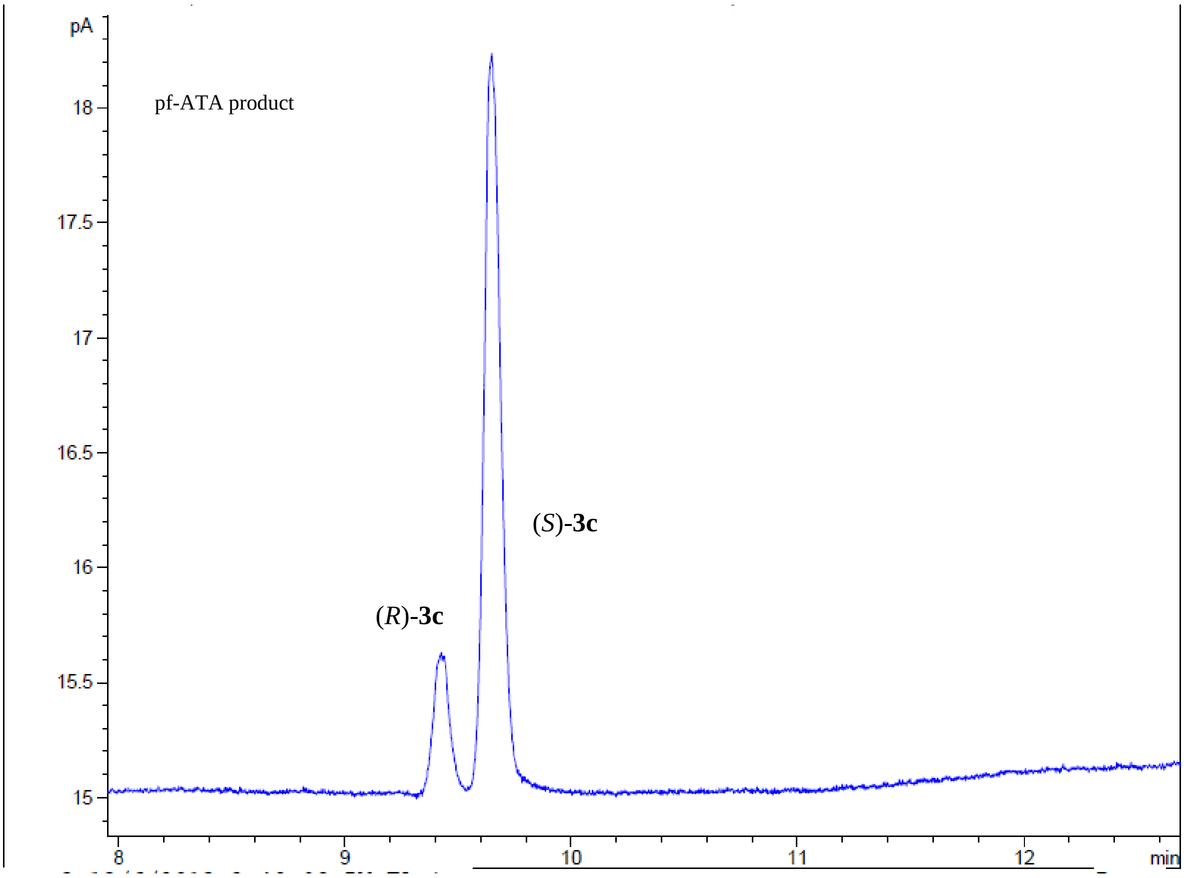
pA
pf-ATA product
(S)-3c
(R)-3c
min

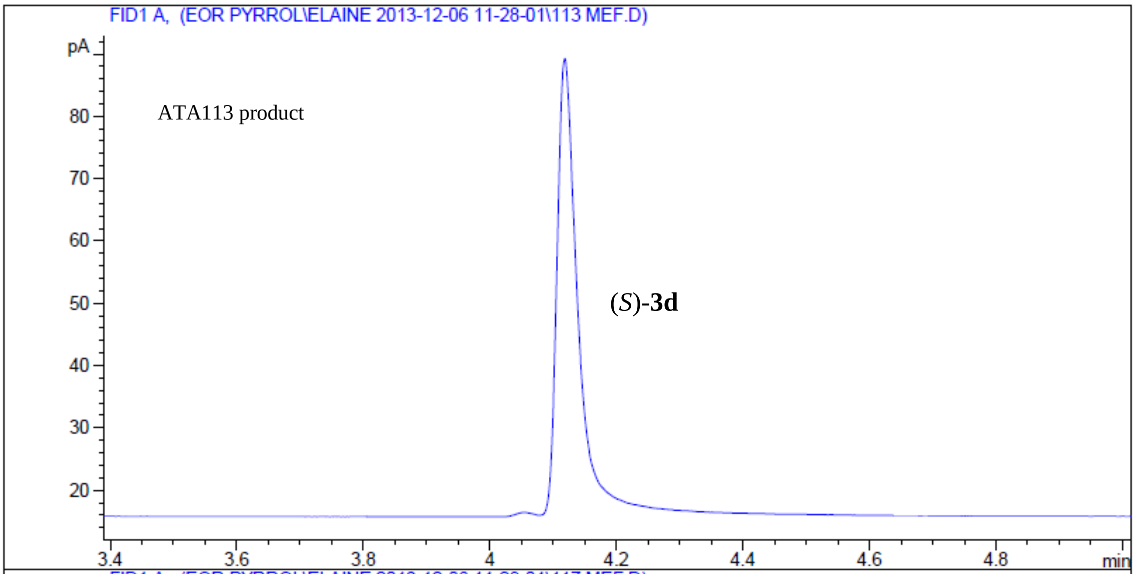
FID1 A, (EOR PYRROL\ELAINE 2013-12-06 11-28-01\113 MEF.D)
pA
ATA113 product
(*S*)-**3d**
min

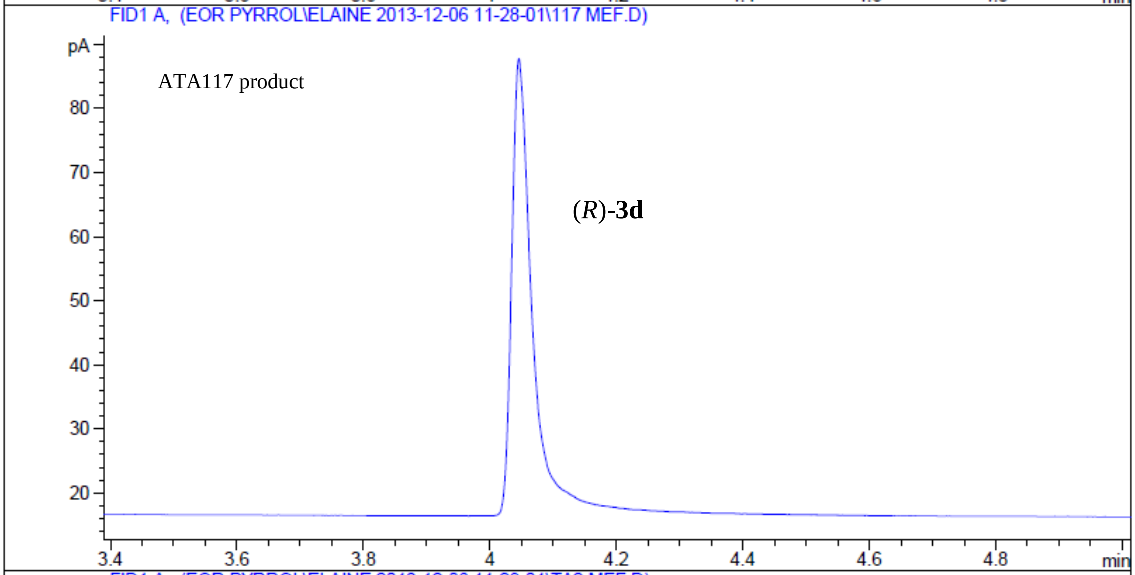
FID1 A, (EOR PYRROL\ELAINE 2013-12-06 11-28-01\117 MEF.D)
pA
ATA117 product
(*R*)-**3d**
min

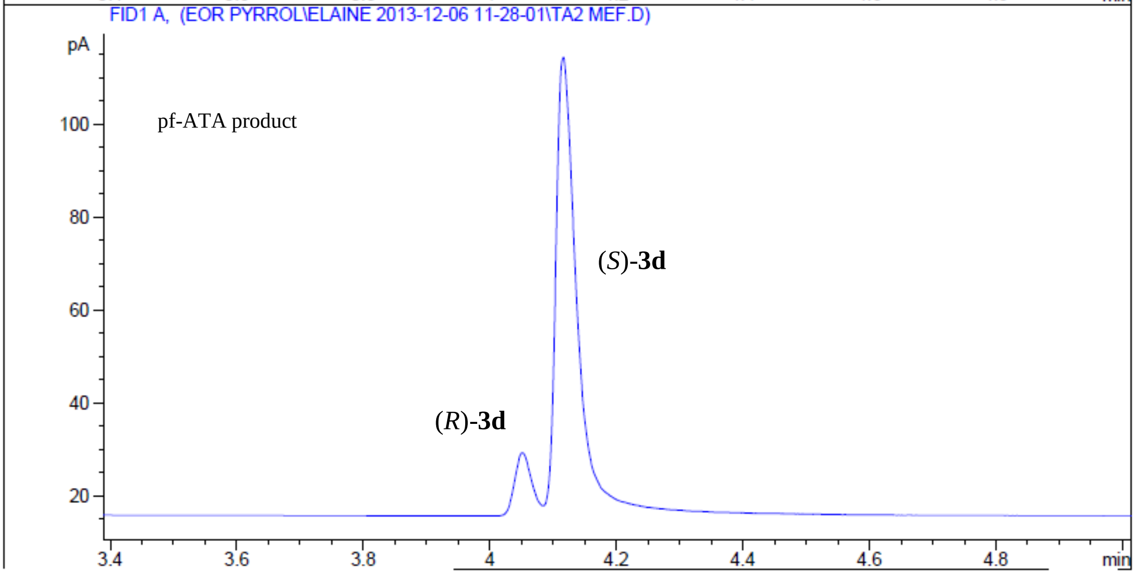
FID1 A, (EOR PYRROL\ELAINE 2013-12-06 11-28-01\TA2 MEF.D)
pA
pf-ATA product
(*S*)-**3d**
(*R*)-**3d**
min

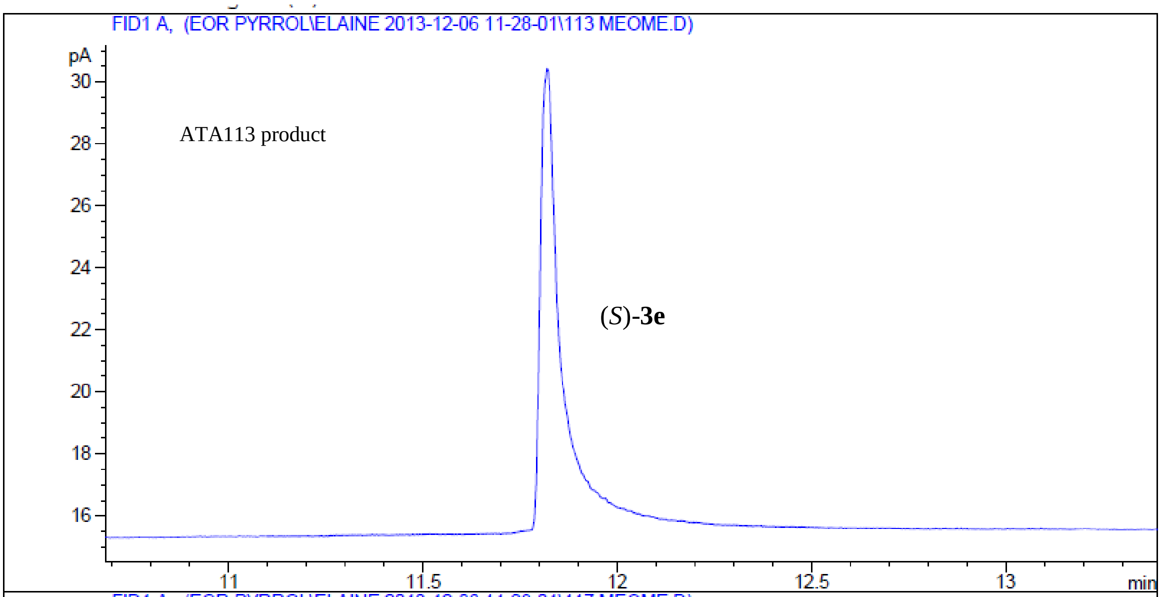
FID1 A, (EOR PYRROL\ELAINE 2013-12-06 11-28-01\113 MEOME.D)
pA
30
28
26
24
22
20
18
16
ATA113 product
(*S*)-**3e**
11
11.5
12
12.5
13
min

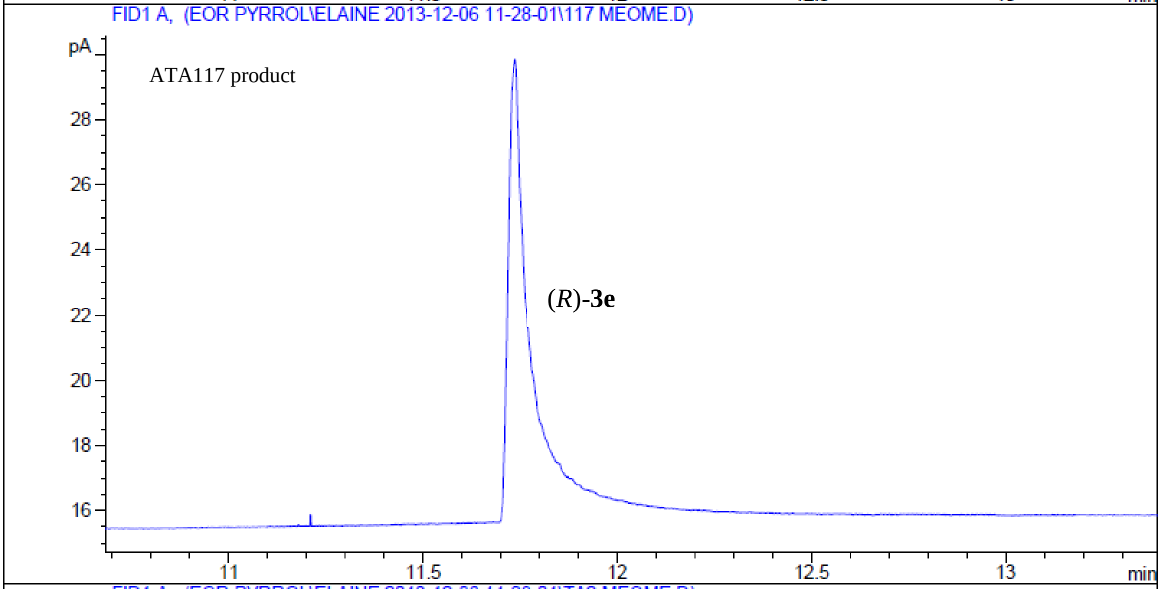
FID1 A, (EOR PYRROL\ELAINE 2013-12-06 11-28-01\117 MEOME.D)
pA
28
26
24
22
20
18
16
ATA117 product
(*R*)-**3e**
11
11.5
12
12.5
13
min

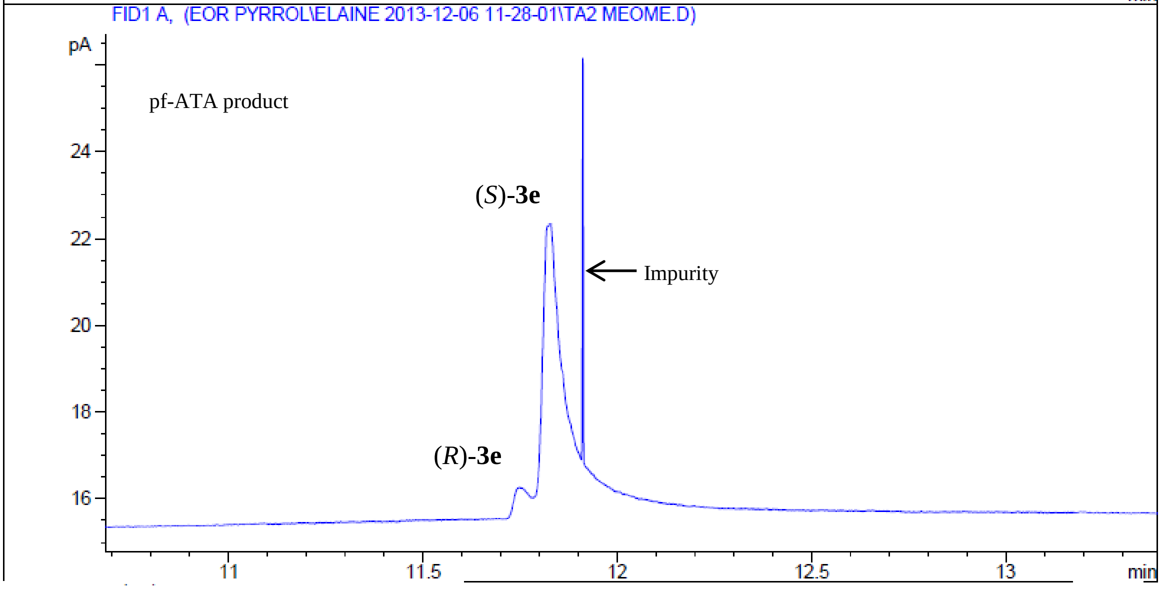
FID1 A, (EOR PYRROL\ELAINE 2013-12-06 11-28-01\TA2 MEOME.D)
pA
24
22
20
18
16
pf-ATA product
(*S*)-**3e**
Impurity
(*R*)-**3e**
11
11.5
12
12.5
13
min

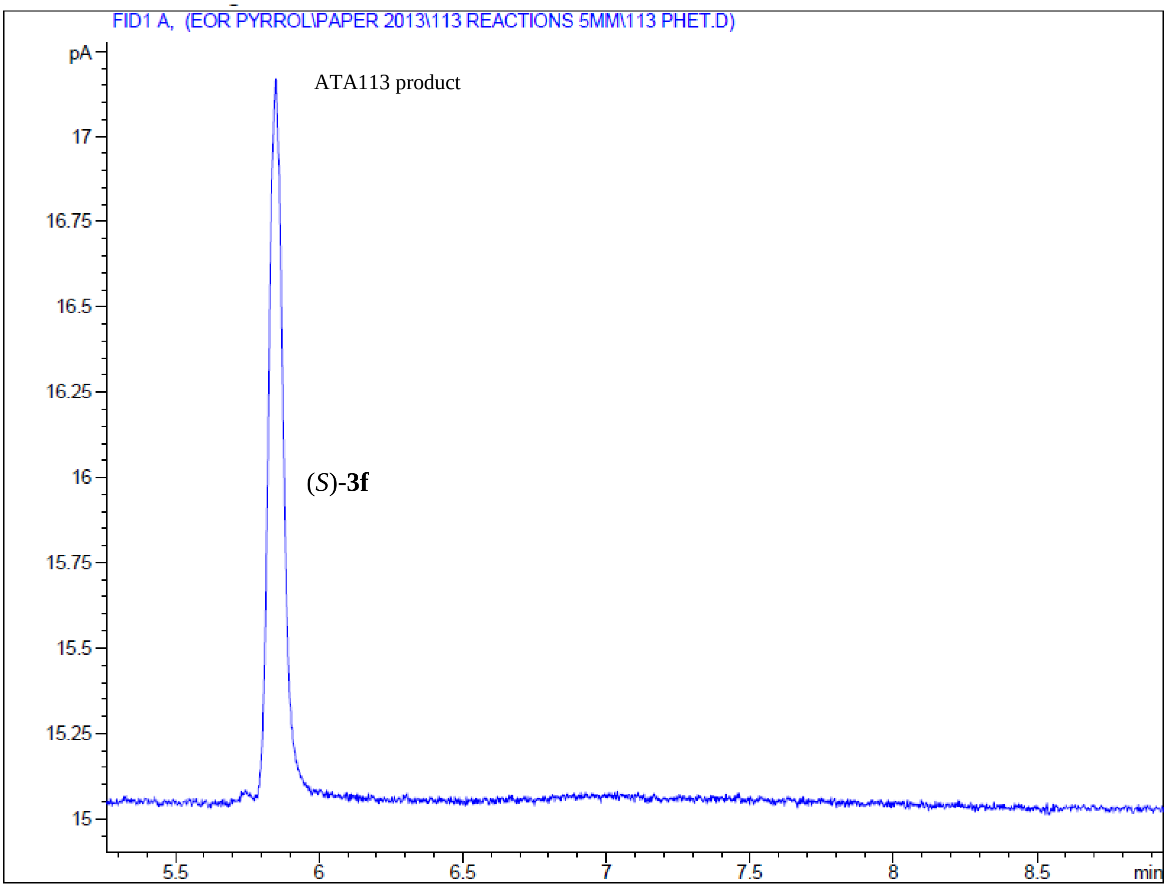
FID1 A, (EOR PYRROL\PAPER 2013\113 REACTIONS 5MM\113 PHET.D)
pA
ATA113 product
(*S*)-**3f**
min

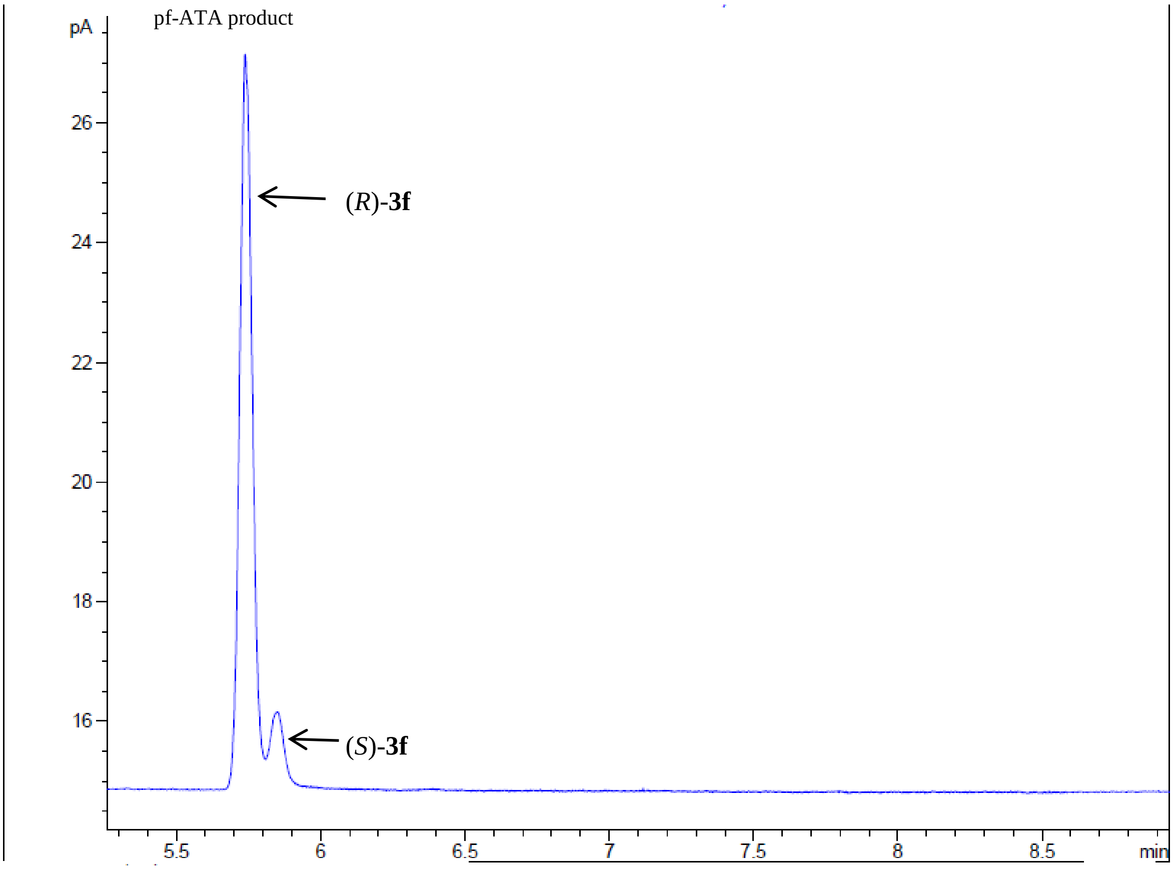
pA
pf-ATA product
(*R*)-**3f**
(*S*)-**3f**
min

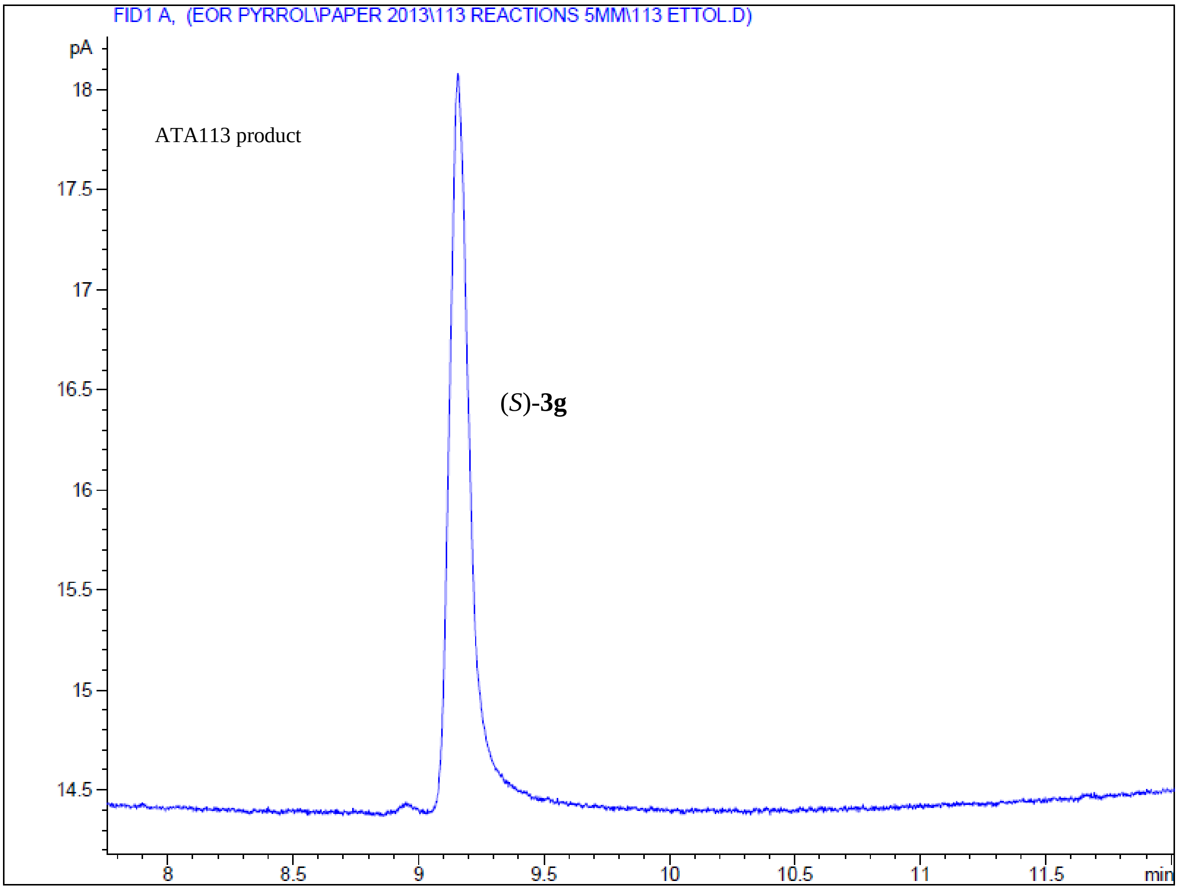
FID1 A, (EOR PYRROL\PAPER 2013\113 REACTIONS 5MM\113 ETTOL.D)
pA
18
17.5
17
16.5
16
15.5
15
14.5
ATA113 product
(*S*)-**3g**
8
8.5
9
9.5
10
10.5
11
11.5
min

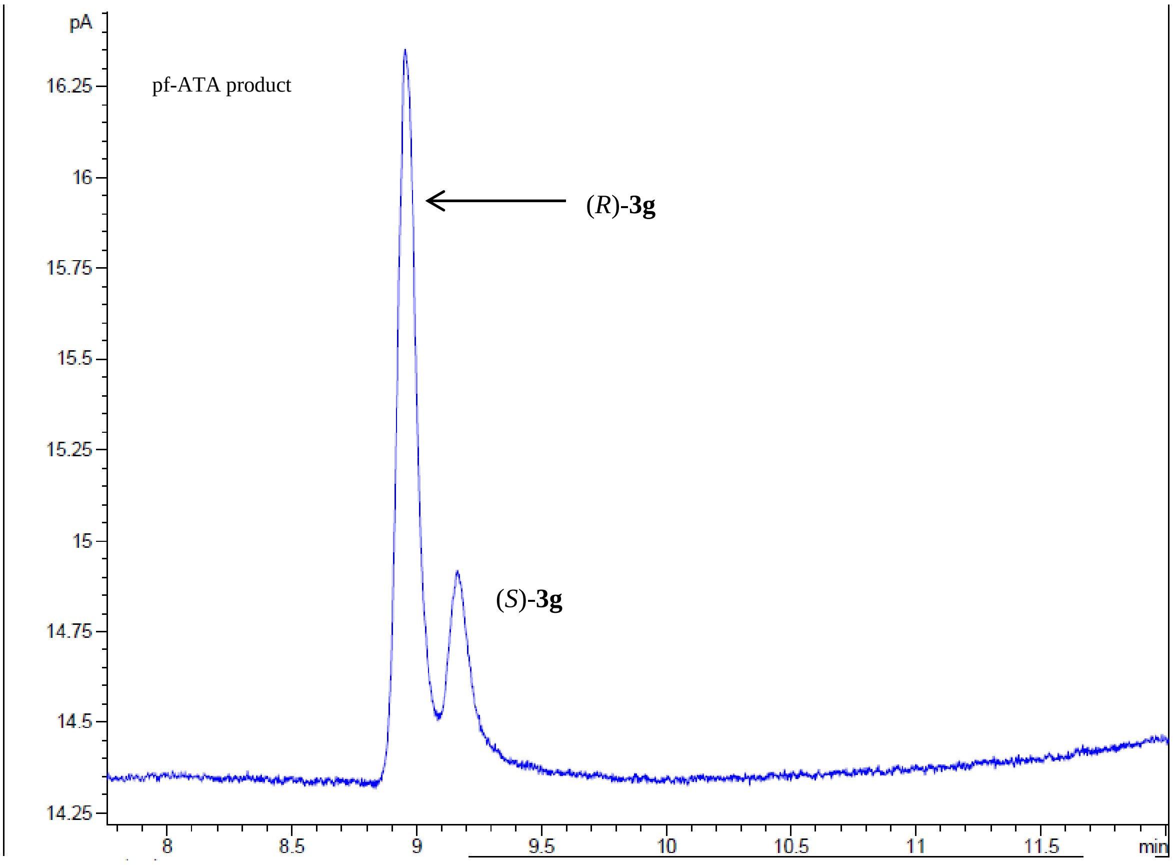
pA
16.25
16
15.75
15.5
15.25
15
14.75
14.5
14.25
pf-ATA product
(*R*)-**3g**
(*S*)-**3g**
8
8.5
9
9.5
10
10.5
11
11.5
min

Determination of *Diastereomeric Excess* of 2,5-disubstituted Pyrrolidines 4a-g After MAO-N Mediated Reaction

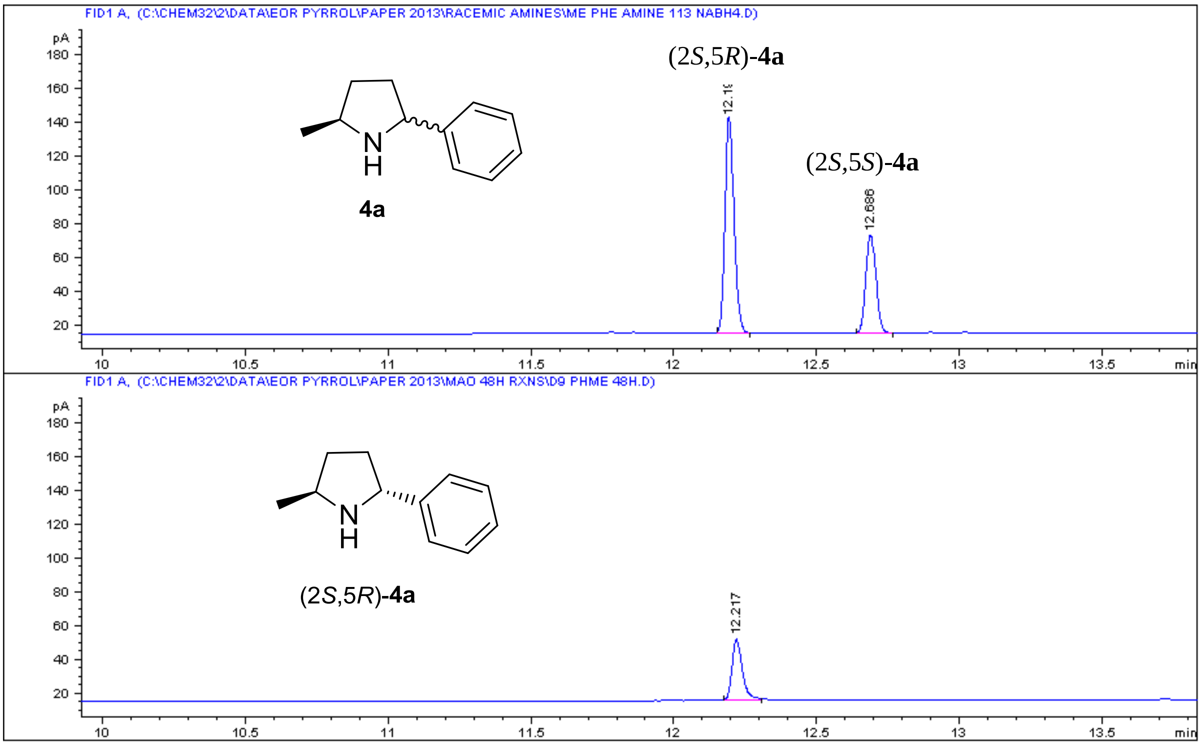

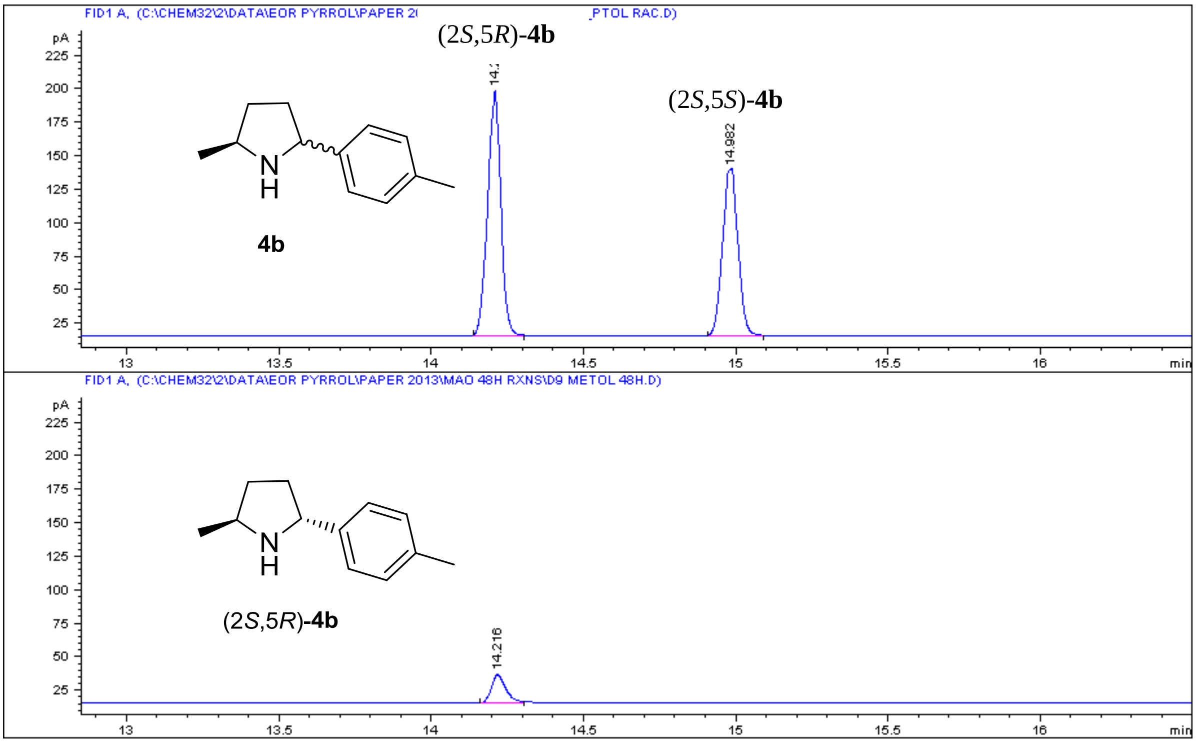

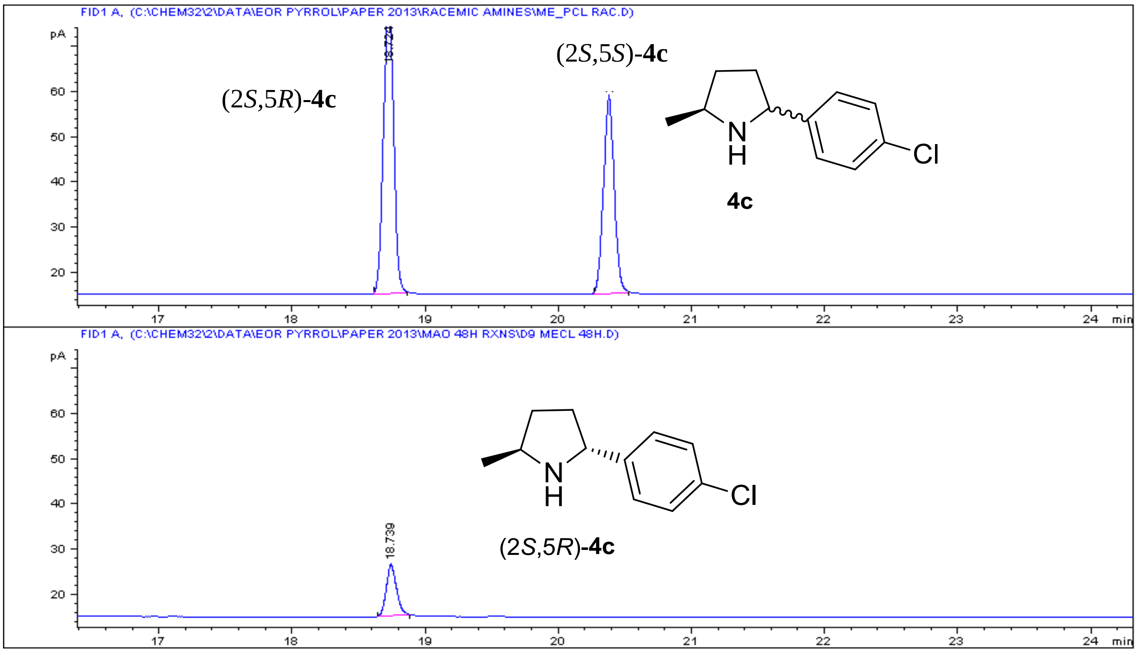
FID1 A, (C:\CHEM32\2\DATA\EOR PYRROL\PAPER 2013\RACEMIC AMINES\ME_PCL RAC.D)
(2S,5R)-4c
(2S,5S)-4c
4c
FID1 A, (C:\CHEM32\2\DATA\EOR PYRROL\PAPER 2013\MAO 48H RXNS\D9 MECL 48H.D)
18.739
(2S,5R)-4c
pA
min

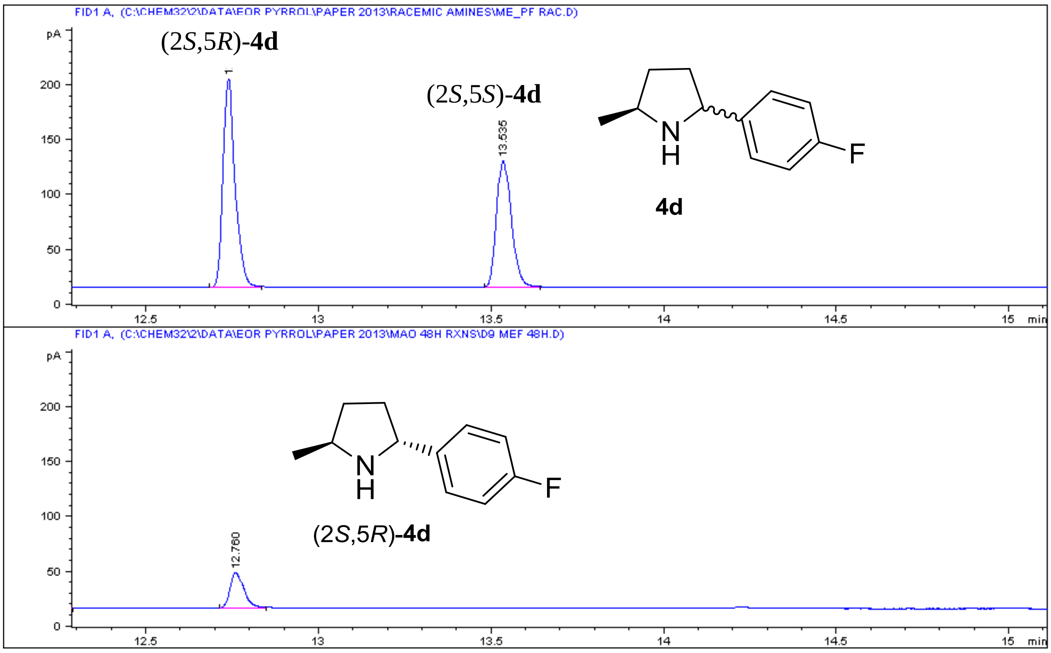
FID1 A, (C:\CHEM32\2\DATA\EOR PYRROL\PAPER 2013\RACEMIC AMINES\ME_PF RAC.D)
(2S,5R)-4d
(2S,5S)-4d
13.535
4d
FID1 A, (C:\CHEM32\2\DATA\EOR PYRROL\PAPER 2013\MAO 48H RXNS\D9 MEF 48H.D)
12.760
(2S,5R)-4d
pA
min

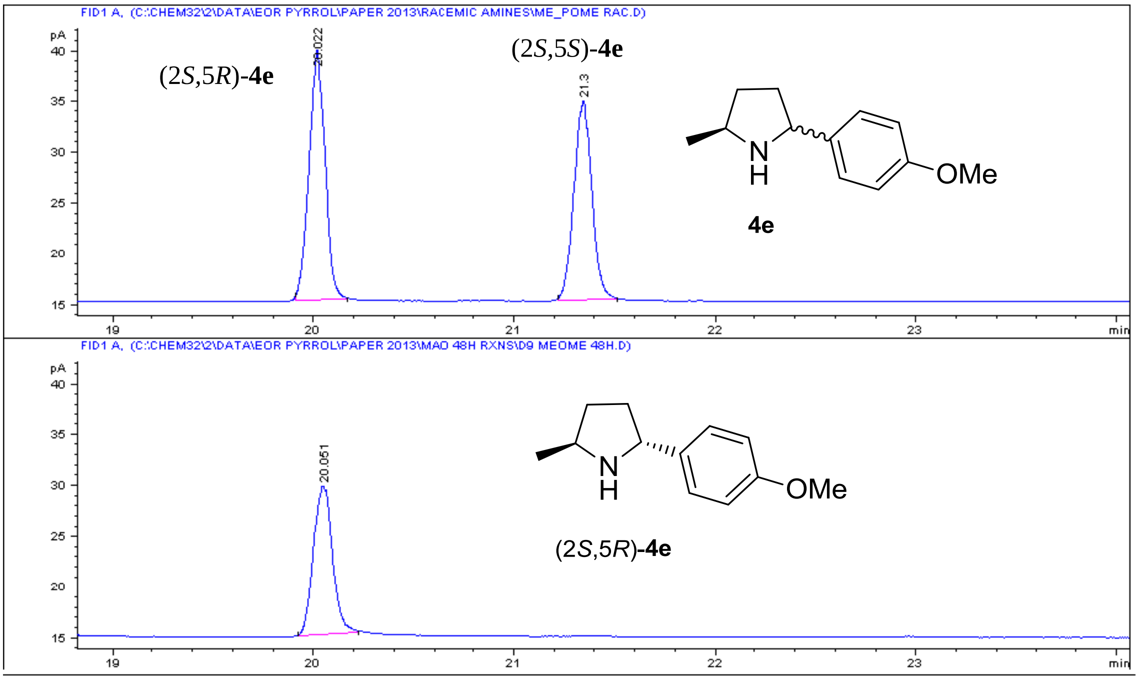
FID1 A, (C:\CHEM32\2\DATA\EOR PYRROL\PAPER 2013\RACEMIC AMINES\ME_POME RAC.D)
pA
20.022
21.3
(2S,5R)-4e
(2S,5S)-4e
N
H
OMe
4e
min
FID1 A, (C:\CHEM32\2\DATA\EOR PYRROL\PAPER 2013\MAO 48H RXNS\D9 MEOME 48H.D)
20.051
OMe
(2S,5R)-4e

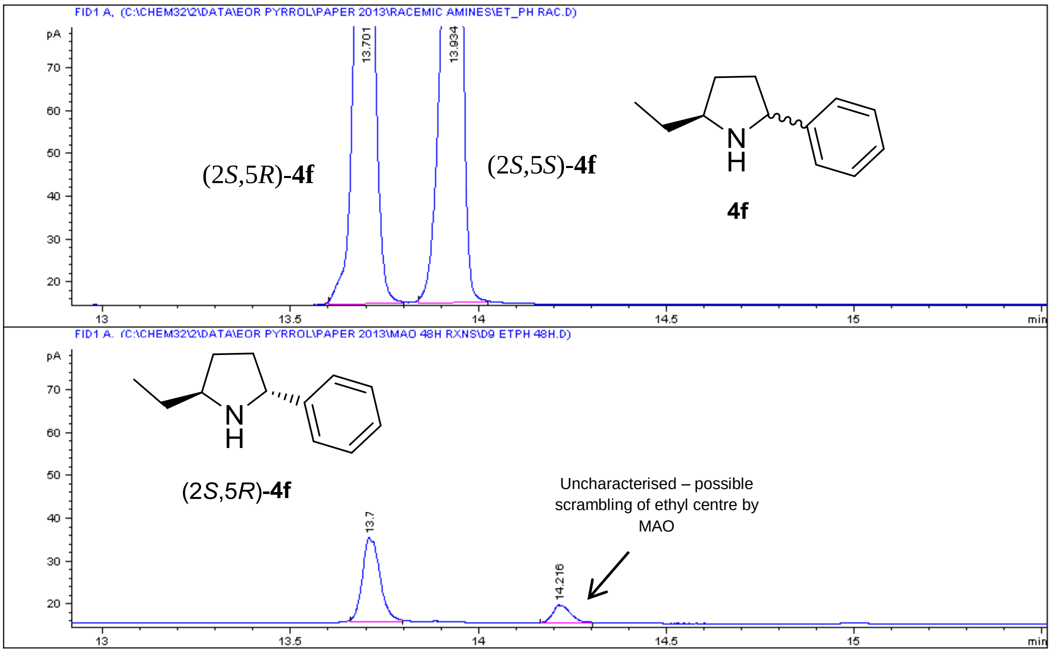
FID1 A, (C:\CHEM32\2\DATA\EOR PYRROL\PAPER 2013\RACEMIC AMINES\ET_PH RAC.D)
pA
13.701
13.934
(2S,5R)-4f
(2S,5S)-4f
N
H
4f
min
FID1 A, (C:\CHEM32\2\DATA\EOR PYRROL\PAPER 2013\MAO 48H RXNS\D9 ETPH 48H.D)
(2S,5R)-4f
13.7
14.216
Uncharacterised – possible scrambling of ethyl centre by MAO

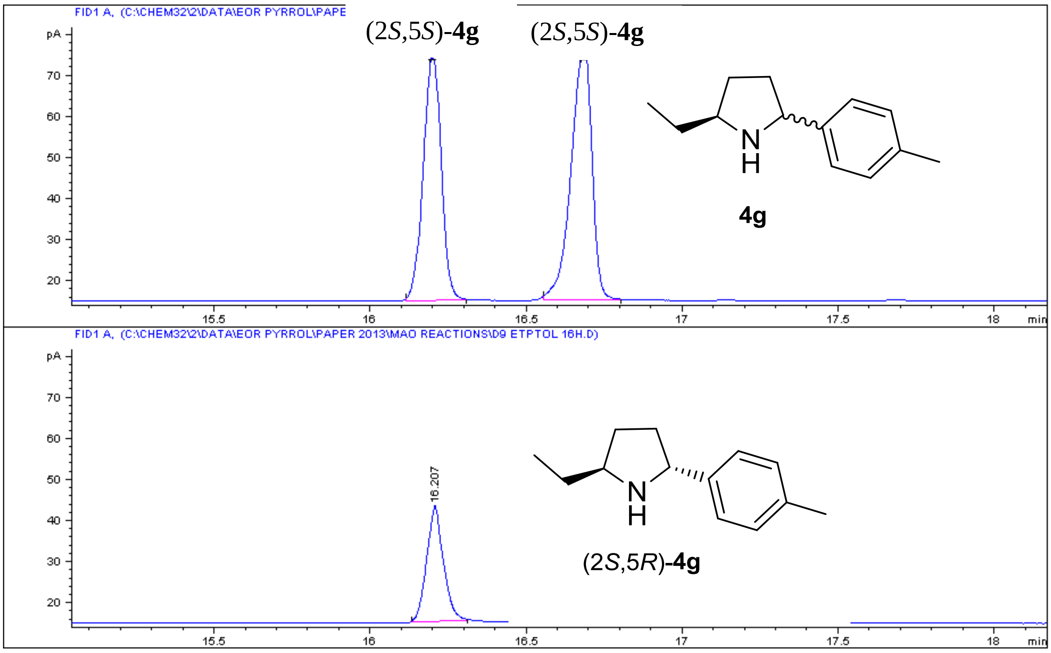

References

S1. D. Koszelewski, M. Göritzer, D. Clay, B. Seisser, W. Kroutil, *ChemCatChem*, **2010**, *2*, 73-77.

S2. Z.-L. Shen, K. K. K. Goh, H.-L. Cheong, C. H. A. Wong, Y.-C. Lai, Y.-S. Yang, T.-P. Loh, *J. Am. Chem. Soc.* **2010**, *132*, 15852-15855.

S3. D.-S. Wang, Z.-S. Ye, Q.-A. Chen, Y.-G. Zhou, C.-B. Yu, H.-J. Fan, Y. Duan, *J. Am. Chem. Soc*. **2011**, *133*, 8866–8869.

S4. D. Ghislieri, A. P. Green, M. Pontini, S. C. Willies, I. Rowles, A. Frank, G. Grogan, N. J. Turner, *J. Am. Chem. Soc.* **2013**, *135*, 10863-10869.
